# Effects of a school-based intervention to reduce cardiovascular disease risk factors among secondary school students: A cluster-randomized, controlled trial

**DOI:** 10.1371/journal.pone.0259581

**Published:** 2021-11-11

**Authors:** John Amoah, Salmiah Said, Lekhraj Rampal, Rosliza Manaf, Normala Ibrahim, Seth Owusu-Agyei, Kwaku Poku Asante

**Affiliations:** 1 Kintampo Health Research Centre, Kintampo, Bono-East, Ghana; 2 Department of Community Health, Faculty of Medicine and Health Sciences, University Putra Malaysia, Serdang, Selangor, Malaysia; 3 Department of Psychiatry, Faculty of Medicine and Health Sciences, University Putra Malaysia, Serdang, Selangor, Malaysia; 4 Department of Public Health, University of Health and Allied Science, Ho, Volta, Ghana; Prince Sattam Bin Abdulaziz University, College of Applied Medical Sciences, SAUDI ARABIA

## Abstract

**Background:**

Cardiovascular diseases (CVDs) are the number cause of death worldwide. In Ghana CVD has been the leading cause of death since 2001. The prevalence of CVD risk factors among adolescents in Ghana has been increasing. This study seeks to develop, implement and evaluate the effects of a behavioral modification intervention program to reduce CVD risk factors among secondary school students in Brong Ahafo, Ghana.

**Methods:**

A cluster-randomized controlled trial was conducted with schools as clusters over a period of six-months with pre and post intervention evaluations. Participants were public secondary school students (14–19 years) from four schools in Brong Ahafo, Ghana. Students in the intervention group were trained by the researchers whereas those of the control group received no intervention. The intervention included health education and physical activity modules. Follow-up data using same questionnaire were collected within two weeks after the intervention was completed. Intention-to-treat analysis was performed after replacing missing values using the multiple imputation method. The generalized linear mixed model (GLMM) was used to assess the effects of the intervention study.

**Results:**

The GLMM analyses showed the intervention was effective in attaining 0.77(p<0.001), 0.72(p<0.001), 0.47(p<0.001), 0.56(p<0.001), and 0.39(p = 0.045) higher total physical activity, fruits, vegetables, seafood, and water scores respectively for the intervention group over the control group. The intervention was also significant in reducing -0.15(p<0.001),-0.23(p<0.001),-0.50(p<0.001),-0.32(p<0.001),-0.90(p<0.001),-0.87(p<0.001),-0.38(p<0.001), -0.63(p<0.001), -1.63(p<0.001), 0.61(p<0.001), and -1.53(p = 0.005) carbohydrates, fats and oils, fried eggs, fried chicken, carbonated drinks, sugar, sweet snacks, salted fish, weight, BMI, and diastolic BP. The odds of quitting alcohol use in the intervention group were 1.06 times more than the control group. There was no significant effect on reducing smoking and systolic BP.

**Conclusion:**

There is an urgent need for the intervention program to be integrated into the existing curriculum structure of secondary school schools. Implementing the intervention will allow for longer and more consistent impact on the reduction of CVD risk factors among secondary school students.

## Introduction

Cardiovascular diseases are the number cause of death worldwide [1–3]. Globally, an estimated 17.9 million people died in 2016 due to CVDs, representing 31% of all global deaths [4]. According to the Ghana Health Service [5], CVDs are the leading causes of NCD deaths with an estimated 35,000 deaths per year. The increasing epidemic is due to lifestyle changes such as physical inactivity, unhealthy diet, smoking and alcohol use.

Though children do not get CVDs, but risk factors that cause the disease begin during the adolescent age [6, 7]. Again, studies have shown that risk factors among adolescent have been increasing. For instance, a nationwide school-based survey in Ghana found the proportion of secondary school students who reported being physically active during the past seven days were only 18.7% [8]. In another study in Ghana among secondary school students revealed the consumption of sweet snacks, sodas, and energy dense foods were high [9]. A research among students (11–17 years) in Ghana revealed smoking prevalence among males and females to be 2.4% and 1.4% respectively [10]. In another research among 1,311 adolescents students in Ghana found prevalence of alcohol use among students was 42.3% [11]. Again, a nationwide study among secondary school students in Ghana reported that 8% of the students were either overweight or obese [8]. Another research in Ghana among 201 youth from three communities found 32.3% pre-hypertensive and 4% hypertensive [12].

Despite CVD preventive measures in Ghana, risk factor of the disease among adolescents and adults have shown increasing trends over the years [8, 13] and therefore the disease keeps on rising and has reached epidemic levels. Also, because risk factors for the development of CVDs begin in childhood, there is therefore an urgent need to educate students on risk factors and prevention of CVDs. Again, the disease can be prevented if interventions that promote reduction of risk factors are implemented among the populace [14] and that addressing a single modifiable risk factor still leaves one at a higher risk of developing CVDs because of failure in tackling the other coexistent risk factors. Also merely educating students on healthy lifestyles without practical interventions may not be enough. Therefore, to reduce this health and economic burden of the disease in Ghana, prevalence of the disease and its risk factors among adolescents must be reduced drastically. In order to achieve this, preventive measures must start as soon as possible among students in the country. This is because school health intervention programs have shown consistent improvement on the general health status of students. Since behavioral change still remains a driving force for reducing CVDs, there is therefore an urgent need for a behavioral change prevention intervention among secondary school students. The aim of this study was to develop, implement, and evaluate the effects of a school-based intervention program on CVD risk factors among public secondary schools in Brong Ahafo Region of Ghana.

## Methods

This study adhered to the Consolidated Statement of Reporting Trials (CONSORT) for cluster randomized controlled trials. The full protocol of this study is published elsewhere [15].

### Research design

The study was a two-arm single blind, parallel cluster randomized controlled trial involving four public secondary school students that were recruited into the study from two selected districts in the Brong Ahafo region. Equal number of schools were allocated to both intervention and control groups, and students in the intervention schools were trained in health education and physical activity modules.

### Study settings and participants

The study was conducted in two districts of the Brong Ahafo region. The region has a population of one million with a large proportion of the region’s population under 15 years while a smaller number is 60 years and above [16]. The two districts were Kintampo-North and Nkoranza-North districts. The study consisted of four public secondary schools. Students who were 14–19 years old in public secondary schools in two districts were included in the study and followed for outcomes for six months. Students who were suffering from serious medical conditions such as heart diseases, asthma, or respiratory conditions or was advised by a medical professional to restrict physical activities/exercise were excluded from the study. The recruitment of students started from December 2017 to October 2018. Recruitment of participants was done after obtaining ethical approvals, which included a local IRB approval (Kintampo Health Research Centre), registration of the study and permissions from the Ministry of Education (Ghana Education Service). These are discussed in detail at the Ethical approvals section. The minimum sample size required for the study was estimated using sample size formula for randomized controlled trials [17] where the total CVD risk factor scores from a previous intervention study were substituted [18]. This gave a total sample size of 281 for where an additional 20% and 1.085 were added for anticipated attrition and design effect for cluster randomized control trials respectively to give a final sample size of 732 participants. After selection of participants, baseline data were collected by trained health staff using a structured questionnaire, before randomly allocating schools to either intervention or control group.

### Trial interventions

The CVD risk factor reduction intervention module was basically divided into two sections. These were the Health Education and the Physical Activity Modules. The interactive health education sections, the researchers did education on CVDs, its risk factors, causes, development and prevention among others in the intervention schools. The intervention schools were visited three times a week for a period of six months. Each section of the health education lasted for about an hour with a break followed by questions, answers and discussions. The physical activity module which consisted of aerobic and anaerobic exercises were delivered by a physical education health instructor. The instructor taught students on the various physical activities and then took them to the school field to undertake the activities. Same exercises were carried out in the intervention schools. Each physical activity session in the intervention group lasted for about 25–30 minutes in Table 1.

**Table 1:** Summary of intervention module.

MODULE	COMPONENTS	DELIVERY	TIME
**Health education module**	Module introduction CVD introduction CVD risk factor introduction Harmful effects of smoking Quitting smoking Barriers to quitting smoking Health benefits of quitting smoking Physical activity introduction Simple physical activities/exercises Fruits and vegetables intake/selection Introduction to types of fats Outcomes of high fat intake Harmful effects of obesity Harmful effects of excessive sugar intake Promotion of frequent water intake Harmful effects of high salt intake Harmful effects of alcohol Quitting alcohol use Barriers to quitting alcohol use Health benefits of quitting/not initiating alcohol	Lectures Discussions	One (1) hour per session
**Physical activity module**	Aerobics **Anaerobic:** Lunge and twist Jumping jacks Abdominal crunch Hamstring stretches Wall sit Side arm and leg raise Push ups Sit and reach Sit ups Knee to chest Leg raises Squat	Hands on (field exercise)	25–30 minutes per session

Training was organized for health staff on anthropometric and blood pressure measurements. The training of health staff was, among other things, to equip them to avoid inter and intra observer measurement biases. Prior to implementation of the trial, a pilot study was conducted before the main study began. The intervention module was reviewed by a panel of experts in public health, behavioral intervention, non-communicable diseases, and health education and promotion.

### Study outcomes and measurements

Post intervention data were collected from participants two weeks immediately after six months with the same questionnaire in the English language. The outcome variables in the study were total physical activity, dietary intake, smoking, alcohol, weight, body mass index, and blood pressure. The questionnaire was divided into eight sessions. The first was participants socio-demographic characteristics; second was physical activity questionnaire (PAQ-A) for secondary school students [19] to measure total levels of physical activity. Each item was scored on a five-point scale. The value from one to five for each of the items used in the physical activity composite score, then the mean of the items which resulted in the final physical activity summary score was used for the participant. The third was a seven-day dietary recall instrument which are foods items mostly consumed in Ghana, was utilized to assess levels of food consumption [20, 21]. The frequency of consumption of foods was scored based on one for never dietary consumption, two for 2–3 times per week, three for 4–5 times per week, four for 6 times per week, and five for daily intake of a particular food item. The fourth and fifth sections was the Global Youth Tobacco Survey (GYTS) developed by WHO and Centre for Disease Control and Prevention [22] was used to assess smoking and alcohol statuses of students. They were classified as never, ever, or current smokers and alcohol users. Sixth was weight measurement using a digital bathroom scale, TANITA Model HD 309 [23, 24] to the nearest 0.1kg. The student was requested to remove slippers or shoe and anything in the pocket. Participant was then asked to stand upright on the scale and the measurement was taken. Seventh was height, measured using SECA Body Meter Model 208 [23, 24] to the nearest 0.1cm. The participant was asked to stand and look straight, barefooted with heels resting together. Height measurement which appeared in the read-off area was recorded. Measuring instruments were standardized daily before they were used. The eighth session was blood pressure measurements. Two BP readings were taken with an Omron HBP-1100 automated BP monitor. This was measured by qualified health staff with the student comfortably seated for five minutes with the legs not crossed and the back and arm supported before measurements were taken. A minute’s rest interval was allowed after the first reading before the second measurement was taken [25]. The average of the two was used to classify students.

### Selection of schools and randomization

There were two public secondary schools in each of the two districts and all were enrolled into the study. Randomization technique in the ratio 1:1 for intervention and control groups respectively were carried out. Secondary schools (clusters) were the unit of randomization. Allocation concealment was achieved with sequentially numbered opaque sealed envelopes by an education officer containing treatment allocation cards. The envelopes were then serially numbered from the outside. Schools were randomly assigned to either the intervention or control group of the study on the same day participants were selected for this study. It was however, performed after baseline data had been collected.

### Sequence generation and blinding

A biostatistician who was not involved in the study generated the allocation sequence. Using a block randomization of two-digit blocks A and B (each containing one intervention and control, to ensure equal distribution in the two groups), two schools each were allocated to intervention and control groups. The study was single blinded. Health staff who took anthropometric and blood pressure measurements were blinded to the groups. Participants were asked to give true responses of what pertained to them in completing the questionnaires and data analyst was also blinded to participant groupings. Researchers and health gym instructor who facilitated the health education and physical activity modules were not blinded. Contamination of the study was minimized since there were intervention and control schools and that the schools were far apart each other.

### Ethical approval

University Putra Malaysia (UPM/TNCPIMC/1.4.18.22) and the Kintampo Health Research Centre (KHRCIEC/2017-16) Ethics Committees approved the study. The study was also registered with trial registration number PACTR201709002540178. Written informed consent was obtained from all students and their corresponding parents or guardians because they were minors. These documents are stored in a locked cabinet at the Kintampo Health Research Centre’s archives office. Permissions were sought from the Ministry of Education, headmasters and teachers.

### Statistical analyses

The data collected was analyzed using Statistical Package for Social Sciences (SPSS) version 22. With the exception of smoking and alcohol variables that were categorical, normality tests were performed on total physical activity, dietary intake, weight, BMI, and blood pressure using kurtosis and histogram plots that showed there were no substantial non-normality and therefore the data was handled as normal data. Chi-square tests were performed to compare baseline characteristics between intervention and control group. The independent and paired t-tests were performed to determine the between and within groups respectively differences of total physical activity, dietary intake, weight, BMI and blood pressure. The Chi-square and Mc-Nemar tests were performed on smoking and alcohol to determine the between and within group differences. Intention-to-treat (ITT) analysis was the method that was used to handle missing data using the multiple imputation method, following which the Generalized Linear Mixed Model (GLMM) analysis was performed to determine the overall effects of the intervention study.

## Results

### Response rate

**Fig 1:**
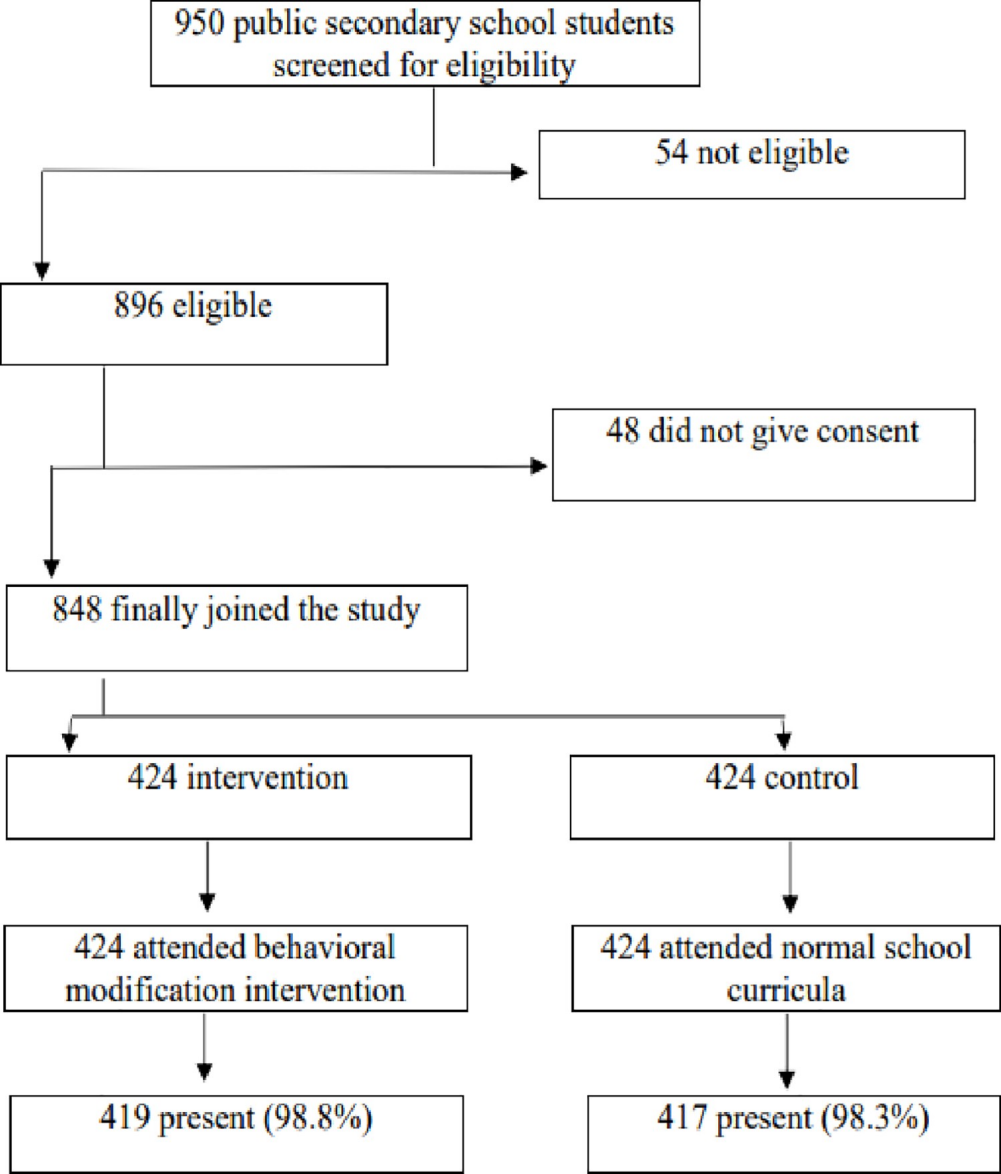
Flow chart of recruitment of participants.

The flow chart for students’ recruitment is presented in Fig 1. Nine hundred and fifty students (950) were assessed from four public secondary schools for possible inclusion into the study where 54 did not meet the inclusion criteria and 48 declined to participate. Then, 848 were randomly assigned to either the intervention (424) or control groups (424). At the end of the study at six months, five (1.2%) had dropped out of the study from the intervention group while the control group was seven (1.7%).

### Baseline characteristics of respondents in intervention and control groups

Table 2 compares socio-demographic characteristics and CVD risk factors between intervention and control groups. There were no significant differences between the two groups at baseline. The ages of respondents ranged from 14–19 years, with mean (SD) age of 16.99 (1.4). Majority of respondents were females (51.3%). A total of 20.9% and 23.5% reported family histories of obesity and hypertension respectively. Mean (SD) physical activity was 2.23 (0.65) in the intervention group and 2.26 (0.60) in the control group. In both groups, mean (SD) consumption of fruits 1.43 (0.48) and fish 1.88 (0.68) were generally low while sugar 2.77 (1.41) and energy drinks 2.04 (1.13) were high. Out of the 40 ever smokers in the intervention group, 34 (85.0%) were current smokers, while 26 ever smokers in the control group, 19 (73.1%) were current smokers. In both groups, majority of students tried their first smoke at age 14 years with age range 11–17 years. Of the 129 ever consumers of alcohol, 98 (76.0%) were current consumers of alcohol and out of 108 ever consumers of alcohol, 90 (83.3%) were current alcohol consumers in the intervention and control groups respectively. In all, 25.5% tried their first alcohol at age 13 years with age range 10–17 years. The mean (SD) weight was 59.19 (8.49) and 59.59 (7.93) in the intervention and control respectively. Mean (SD) BMI in the intervention group was 21.75 (2.85) while the control group was 21.86 (2.47). In both intervention and control groups, 93 (11.0%) were classified as overweight, while 19 (2.2%) were found to be obese. Mean (SD) diastolic BP was 67.66 (7.88) and 68.28 (8.11) and systolic BP was 111.64 (10.14) and 112.30 (9.48) in the intervention and control respectively.

**Table 2:** Baseline comparison between intervention and control groups.

	Intervention		Control		
Baseline variables	Frequency (%) /Mean (SD)/Chi- Square (n = 424)		Frequency (%) /Mean (SD)/Chi- Square (n = 424)		*p value*
**Age (years)**					0.310
Mean (SD)	17.05	(1.4)	16.94	(1.4)	
(Range)		(14–19)		(14–19)	
**Gender**					0.731
Male	208	(49.1)	203	(47.9)	
Female	216	(50.9)	221	(52.1)	
**Family history of obesity**					0.076
Yes	99	(23.3)	78	(18.4)	
No	325	(76.7)	346	(81.6)	
**Family history of hypertension**					0.685
Yes	102	(24.1)	97	(22.9)	
No	322	(75.9)	327	(77.1)	
**Primary outcomes**					
Physical activity	2.32	(0.65)	2.26	(0.60)	0.180
**Diet**					
Fruits	1.41	(0.50)	1.44	(0.46)	0.297
Vegetables	2.27	(0.65)	2.27	(0.66)	0.949
Seafood	1.91	(0.64)	1.84	(1.71)	0.162
Plain water	3.93	(1.21)	4.01	(1.19)	0.289
Carbohydrates	1.65	(0.60)	1.70	(0.57)	0.192
Fats and oils	1.49	(0.48)	1.49	(0.50)	0.888
Fried eggs	1.64	(1.00)	1.65	(0.98)	0.835
Fried chicken	1.90	(1.01)	1.88	(1.02)	0.709
Carbonated drinks	1.90	(1.01)	1.88	(1.02)	0.708
Palin sugar	2.81	(1.40)	2.73	(1.41)	0.367
Sweet snack	1.69	(0.60)	1.63	(0.52)	0.123
Salted fish	1.62	(0.98)	1.17	(0.99)	0.198
**Current smoker**	(n = 40)		(n = 26)		0.234
Yes	34	(85.0)	19	(73.1)	
No	6	(15.0)	7	(26.9)	
**Current alcohol use**	(n = 129)		(n = 108)		0.163
Yes	98	(76.0)	90	(83.3)	
No	31	(24.0)	18	(16.7)	
**Secondary outcomes**					
Weight (Kg)	59.19	(8.49)	59.59	(7.93)	0.478
BMI (kg/m^2^)	21.75	(2.85)	21.86	(2.47)	0.540
Diastolic (mmHg)	67.66	(7.88)	68.28	(8.11)	0.256
Systolic (mmHg)	111.64	(10.14)	112.30	(9.48)	0.347

### Follow up comparison within and between groups at six months post intervention

Table 3 presents the differences in the outcome variables at six months follow-up between the intervention and control groups. The intervention group had significantly higher mean difference in total physical activity than the control group 0.81 (0.74–0.89). On the other hand, consumption of fruits, vegetables, seafood and water increased significantly while the consumption of carbohydrates, fried eggs, fried chicken, carbonated drinks, sugar, sweet snacks and salted fish decreased significantly in the intervention group when compared to the control group. Further, there were significant decrease in the intervention arm of the study compared to the control arm for BMI -0.74 (-1.10, -0.37), diastolic -1.78 (-2.86, -0.71) and systolic BP -1.69 (-3.00, -0.37).

**Table 3:** Changes from baseline to six months and comparison between groups post intervention.

	Intervention group			Control group			Between group follow up	
Outcome variables	n	Mean (SD)	Mean change from baseline (95% CI)	n	Mean (SD)	Mean change from baseline (95% CI)	Mean difference (95% CI)	*p-value*
Physical activity	419	3.10 (0.52)	0.79 (0.73–0.84)	417	2.29 (0.58)	0.03 (0.02–0.07)	0.81 (0.74–0.89)	<0.001
**Diet**								
Fruits	419	2.17 (0.49)	0.76 (0.71–0.80)	417	1.48 (0.42)	0.03 (0.01–0.05)	0.69 (0.63–0.75)	0.001
Vegetables	419	2.62 (0.63)	0.36 (0.32–0.39)	417	2.17 (0.59)	-0.10 (-0.13, -0.08)	0.45 (0.37–0.54)	<0.001
Seafood	419	2.42 (0.86)	0.52 (0.44–0.59)	417	1.82 (0.62)	-0.03 (-0.07, -0.02)	0.60 (0.50–0.70)	<0.001
Plain water	419	4.40 (0.90)	0.48 (0.35–0.60)	417	4.06 (1.24)	0.03 (0.14–0.20)	0.35 (0.19–0.49)	<0.001
Carbohydrate	419	1.58 (0.42)	-0.07 (-0.12, -0.02)	417	1.68 (0.57)	0.16 (0.13–0.19)	-0.28 (-0.35, -0.21)	<0.001
Fats and oils	419	1.26 (0.32)	-0.23 (-0.27, -0.20)	417	1.72 (0.51)	0.24 (-0.21, 0.27)	-0.47 (-0.52, -0.41)	<0.001
Fried eggs	419	1.42 (0.80)	-0.22 (-0.29, -0.15)	417	1.95 (1.19)	0.29 (-0.37, 0.21)	-0.53 (-0.67, -0.39)	<0.001
Fried chicken	419	1.49 (0.66)	-0.40 (-0.49, -0.39)	417	1.81 (1.21)	0.06 (-0.06, 0.19)	-0.31 (0.44, -0.18)	<0.001
Carbonated drinks	419	1.30 (0.55)	-0.59 (-0.69, -0.49)	417	2.18 (1.42)	0.31 (0.15–0.48)	-0.88 (-1.02, -0.73)	<0.001
Plain sugar	419	2.04 (1.18)	-0.77 (-0.91, -0.63)	417	2.83 (1.41)	0.09 (0.02–0.17)	-0.78 (-0.96, -0.61)	<0.001
Sweet snack	419	1.44 (0.38)	-0.25 (-0.28, -0.21)	417	1.76 (0.53)	0.14 (0.11–0.16)	-0.33 (-0.39, -0.26)	<0.001
Salted fish	419	1.27 (0.58)	-0.36 (-0.44, -0.27)	417	1.98 (1.14)	0.09 (0.19–0.34)	-0.71 (-0.83, -0.59)	<0.001
Weight (Kg)	419	58.47 (8.49)	-0.72 (0.85, -0.59)	417	60.55 (7.83)	0.92 (0.80–1.04)	-2.08 (-3.19, -0.97)	<0.001
BMI (Kg/m^2^)	419	21.47 (2.85)	-0.27 (-0.32, 0.22)	417	22.21 (2.48)	0.34 (0.30–0.39)	-0.74 (-1.10, -0.38)	<0.001
Diastolic BP (mmHg)	419	66.80 (7.67)	-0.84 (-1.10, -0.59)	417	68.51 (8.12)	0.26 (0.18–0.35)	-1.78 (-2.86, -0.17)	0.001
Systolic BP (mmHg)	419	111.13 (9.87)	-0.52 (-0.65, 0.39)	417	112.82 (9.56)	0.34 (0.65–0.56)	-1.69 (-3.00, -0.37)	0.012

### Follow-up comparison between and within groups smoking and alcohol use at six months

Table 4 compares smoking and alcohol use at six months between the two groups. There was a statistically significant difference of current and ex-smokers between the two groups at six months. There were more quitters of smokers in the intervention group while the control group remained the same. Similar trend was also seen in alcohol use. There was a statistically significant difference of current alcohol and ex-alcohol users between the two groups at six months post intervention study. There were more ex-alcohol users in the intervention group than the control group at six months and was significant.

**Table 4:** Comparison of smoking and alcohol statuses of intervention and control groups at six months post intervention.

		Groups			
Variable	Intervention		Control		*p-value*
	Frequency (%)		Frequency (%)		
**Smoking status**	(n = 34)		(n = 19)		<0.001
Current smoker	1	(2.9)	19	(100)	
Ex-smoker	33	(97.1)	0	(0)	
**Alcohol status**	(n = 98)		(n = 90)		<0.001
Current use	5	(5.1)	87	(96.7)	
Ex-alcohol use	93	(94.9)	3	(3.3)	

Table 5 illustrates the McNemar test to assess smoking and alcohol use from baseline to six months post intervention. There was a significant reduction in the proportion of current smokers at baseline (n = 34) to six months post intervention 1 (2.9%) among participants in the intervention group. Smoking status in the control group however, remained the same from baseline to six months post intervention study. Similarly at six months, current alcohol use had reduced significantly from baseline (n = 98) to posttest 5 (5.1%) among students in the intervention group. In the control group, there was no significant reduction in the proportion of current alcohol use from baseline (n = 90) to posttest 87 (96.7%) at six months.

**Table 5:** Change in frequency of smoking and alcohol statuses from baseline to post intervention.

Groups		Frequency (%)		*p-value*
**Smoking status**				
**Intervention**				<0.001
**Baseline**		**Post intervention**		
Current smoker	Ex-smoker	Current smoker	Ex-smoker	
34 (85)	6 (15)	1 (2.9)	33 (97.1)	
**Control**				1.000
**Baseline**		**Post intervention**		
Current smoker	Ex-smoker	Current smoker	Ex-smoker	
19 (73.1)	7 (26.9)	19 (100)	0 (0)	
**Alcohol status**				
**Intervention**				<0.001
**Baseline**		**Post intervention**		
Current use	Ex-alcohol use	Current use	Ex-alcohol use	
98 (76.0)	31 (24.0)	5 (5.1)	93 (94.9)	
**Control**				0.250
**Baseline**		**Post intervention**		
Current use	Ex-alcohol use	Current use	Ex-alcohol u	
90 (83.3)	18 (16.7)	87 (96.7)	3 (3.3)	

### Main effects of the intervention

In the GLMM, the ITT was the basis for analysis where missing values were replaced using multiple imputation method. Analysis of missing values showed 1.18% and 1.65% in intervention and control groups respectively and therefore little’s missing completely at random (MCAR) test was not significant for both groups = 146.262, *df* = 185, *p* = 0.984. Students’ socio-demographic factors were controlled for, with combination of variables gave the best model fit and had the lowest Akaike Corrected Information Criterion (ACIC) and Bayesian Information Criterion (BIC).

### Magnitude of intervention effect

Table 6 presents the fixed coefficients of the outcome variables that were studied. The results showed that a student in the intervention arm of the study was expected to lead to an increase in physical activity by 0.77 times and to decrease weight and BMI by -1.63 and -0.61 respectively as compared to an individual in the control arm of the study. Further, an expected decrease of -1.53 diastolic BP was expected for individuals in the intervention group as compared to individuals in the control arm. However, no significant decrease in systolic BP was expected between the two groups. On dietary habits, a student in the intervention arm was also expected to increase consumption of fruits (0.72 times), vegetables (0.47), seafood (0.56 times), and water (0.39 times) as compared to an individual in the control group. Furthermore, an intervention student was expected to decrease consumption of carbohydrates (-0.15 times), fats and oils (-0.23 times), fried eggs (-0.50 times), fried chicken (-0.32 times), carbonated drinks (-0.90 times), plain sugar (-0.87 times), sweet snack (-0.38 times), and salted fish (-0.63) when compared to an individual student in the control arm. There was also a significant effect between groups (F (1, 438) = 13.48, *p<*0.001) on alcohol consumption. The odds of quitting alcohol use in the intervention were 1.06 times (t = -5.176, *p<*0.001) more than the odds of quitting alcohol in the control group after the intervention when compared to the control. There was however, no significant difference between the two groups on smoking.

**Table 6:** Fixed coefficient of outcome variables.

Variable	Coefficient	Std error	Sig./OR		95% CI	
					Lower	Upper
**Physical activity**						
Intervention	0.77	0.130	<0.001		0.51	0.82
Control	1					
**Fruits**						
Intervention	0.72	0.092	<0.001		0.54	0.90
Control	1					
**Vegetables**						
Intervention	0.47	0.015	<0.001		0.44	0.49
Control	1					
**Seafood**						
Intervention	0.56	0.041	<0.001		0.48	0.64
Control	1					
**Plain water**						
Intervention	0.39	0.195	0.045		0.21	0.78
Control	1					
**Carbohydrate**						
Intervention	-0.15	0.018	<0.001		-0.19	-0.12
Control	1					
**Fats and oils**						
Intervention	-0.23	0.015	<0.001		-0.26	-0.21
Control	1					
**Fried eggs**						
Intervention	-0.50	0.060	<0.001		-0.61	-0.38
Control	1					
**Fried chicken**						
Intervention	-0.32	0.051	<0.001		-0.42	-0.22
Control	1					
**Carbonated drinks**						
Intervention	-0.90	0.093	<0.001		-1.09	-0.72
Control	1					
**Plain sugar**						
Intervention	-0.87	0.199	<0.001		-1.26	-0.48
Control	1					
**Sweet snack**						
Intervention	-0.38	0.015	<0.001		-0.41	-0.35
Control	1					
**Salted fish**						
Intervention	-0.63	0.048	<0.001		-0.73	-0.54
Control	1					
**Smoking**						
Intervention	1.81	0.805	0.078	0.34	0.28	1.27
Control	1					
**Alcohol**						
Intervention	-1.57	0.304	<0.001	1.06	0.59	1.92
Control	1					
**Weight**						
Intervention	-1.63	0.376	<0.001		-2.36	-0.89
Control	1					
**BMI**						
Intervention	-0.61	0.145	<0.001		-0.90	-0.33
Control	1					
**Diastolic BP**						
Intervention	-1.53	0.547	0.005		-2.60	-0.45
Control	1					
**Systolic BP**						
Intervention	-0.96	0.075	0.880		-1.11	-0.82
Control	1					

OR, odd**s ratio**

## Discussions

The study was a cluster randomized controlled trial among public secondary school students in Ghana, evaluating the effects of an intervention to reduce CVD risk factors. The intervention group of the study was effective in improving total physical activity levels, fruits, vegetables seafood and water consumption. The intervention was also effective in reducing the consumption of carbohydrates, fats and oils, sweet snacks, carbonated drinks, fried eggs, fried chicken, salted fish, alcohol use and diastolic BP when compared to the control group. The intervention was however, not effective in reducing smoking and systolic BP. This present study was unique in that the intervention was directed at secondary school students to improve healthy lifestyle behaviors. This is among a few controlled trials that examined the effects of school-based health education intervention for CVD risk factor reduction in Ghana.

The significant improvement of physical activity levels in the intervention schools is attributed to the physical activity module that was implemented among students in the intervention group of the study. All students in the intervention schools participated in the same physical activities to ensure uniformity. Students were taken through the various forms of physical activities by the health education instructor before they were taken to the school field to do the various forms of exercise. The physical activity module of the intervention consisted of aerobic and anaerobic exercises and students took keen interest during the sessions and participated fully. This is consistent with a school-based intervention study that showed significant improvements in physical activity in the intervention group when compared to the control arm of the study [26].

The intervention was also effective in improving both consumption of healthy foods and the reduction of unhealthy diets. This improvement in particular was simply as a result of the knowledge transferred to students during the intervention group. In addition, reduction of food items such as fried eggs, fats and oils and carbonated drinks among others meant that the module on dietary habits was effective when compared to the control schools. Also, because students may have transferred the knowledge gained home, could benefit the family in general. This is consistent with studies that showed significant improvements among students in the intervention arm of the study [27–29].

Similarly, there was significant reduction of alcohol use among students in the intervention schools of the study. This meant that the odds of quitting alcohol use were higher in the intervention compared to the control arm. There were however, no effects of smoking between the two groups. The impact on reduction of alcohol use was due to alcohol reduction module that was carried out within the intervention schools. During the intervention students were informed and encouraged to stop or not initiate alcohol consumption. This reduction is important in preventing hypertension and CVDs. This is similar to studies that showed reduction of alcohol use among young people [30].

On the other hand, the intervention was significant in reducing body weight and BMI. The significant reduction in weight and BMI that were seen among students in the intervention group were as a result of the dietary modification and the physical activity modules that were delivered in the intervention schools. The improvement in physical activities and the healthy dietary intake with corresponding reduction in energy dense foods, sweet snacks, fats and oils, and carbonated drink resulted in both weight and BMI reductions. This is consistent with a study that showed significant reduction of weight and BMI at the end of the study [31].

There was significant reduction of diastolic BP among students in the intervention group when compared to the control arm of the study. The significant reduction in diastolic BP was due to improved physical activity levels and healthy eating habits among the participants in the intervention group as a result of the implemented modules. This is because unhealthy diet and physical inactivity are known to cause hypertension. This is consistent with school-based studies which showed significant reduction in systolic and diastolic [32, 33] in the interventions group at the end of the study compared to this study which findings showed only significant reduction of diastolic BP but not systolic BP.

### Strength and limitation

The present study has several strengths. First, the cluster randomized controlled trial design used in this study prevented the possibility of cross contamination between the intervention and control groups. Secondly, the use of standard measurement equipment and single blinding in this study prevented measurement bias. The third strength was that the attrition rate was minimal because of the longer hours’ students spent in school and the different diversity, making it an ideal place for health intervention programs. High response rate in this study at six months helped prevented follow-up bias. The study also had some limitations. First, with the questionnaire as a tool of data collection, a lot depended on the truthfulness of respondents. Secondly, a longer period follow-up may be more suitable in determining behavior change.

### Implication

Our results could be beneficially applied to secondary schools in Ghana to reduce risk factors of CVDs to subsequently reduce CVDs and generally non-communicable diseases among the populace. The knowledge gained could be transferred to peers and the home.

## Conclusion

A six-month school-based CVD risk factor reduction intervention was effective in improving physical activity, fruits, vegetables, seafood, plain water, and reduction of alcohol consumption, carbohydrates, fats and oils, fried eggs, fried chicken, carbonated drinks, sweet snacks, salted fish, weight, BMI, and diastolic BP of secondary school students. There were no significant effects of the intervention in reducing smoking and systolic BP. It is recommended that the intervention be adapted and integrated into the curriculum structure of secondary schools in Brong Ahafo region. Implementing the intervention in schools will allow for longer and more consistent impact on reduction of CVD risk factors among students.

## Supporting information

S1 Checklist CONSORT 2010 checklist of information to include when reporting a randomised trial*. (DOC)

S1 File Study protocol. (DOCX)

S2 File Working dataset. (SAV)
