# Supplementary material for: Effects of a school-based intervention to reduce cardiovascular disease risk factors among secondary school students: A cluster-randomized, controlled trial
Source: PLoS One. 2021 Nov 11;16(11):e0259581. doi: 10.1371/journal.pone.0259581 (PMC8584749; doi:10.1371/journal.pone.0259581)
Supplement: S1 File — (DOCX) [file pone.0259581.s002.docx]

**EFFECTIVENESS OF BEHAVIORAL MODIFICATION INTERVENTION TO REDUCE CARDIOVASCULAR DISEASE RISK FACTORS AMONG PUBLIC SECONDARY SCHOOL STUDENTS**

**IN BRONG AHAFO, GHANA**

**STUDY PROTOCOL**

By

**JOHN AMOAH**

**ABSTRACT**

**EFFECTIVENESS OF BEHAVIORAL MODIFICATION INTERVENTION TO REDUCE CARDIOVASCULAR DISEASE RISK FACTORS AMONG PUBLIC SECONDARY SCHOOL STUDENTS**

**IN BRONG AHAFO, GHANA**

By

**JOHN AMOAH**

Globally, cardiovascular disease (CVD) was responsible for 17.5 million deaths, accounting for 46.2% of non-communicable diseases (NCDs) deaths. In Ghana CVDs has been the leading cause of death since 2001. The prevalence of CVD risk factors among adolescents and adults in Ghana has been increasing. The main aim of this study is to develop, implement and evaluate the effectiveness of a behavioral modification intervention program to reduce cardiovascular disease risk factors among secondary school students in Brong Ahafo, Ghana. The study will be a single blind cluster randomized controlled trial. Baseline data will be collected from 848 students after which schools will evenly be randomized using block randomization (1:1 ratio) of two digit blocks (A and B). School-based intervention will be implemented using the Information- Motivation-Behavioral skills (IMB) model to reduce CVD risk factors over a period of six months with pre and post intervention evaluations. The intervention module will be a health education and physical activity modules in the intervention schools. The control schools will go on with their normal school curriculum and will be waitlisted for similar intervention after the study. Follow-up data using same questionnaire will be collected within two weeks after the intervention is completed. The primary outcomes are knowledge, motivation, behavioral skills, physical activity, smoking, alcohol, dietary intake, while weight, body mass index, and blood pressure were secondary outcomes. Weight, height and blood pressure will be measured using standardized equipment by trained health staff. Intention-to-treat analysis will be performed after replacing missing values using multiple imputation method. The generalized linear mixed model (GLMM) will be used to test the effect of group, time and group-time interactions after controlling for potential confounders.

The study is expected to improve knowledge, motivation, behavioral skills, physical activity, and healthy eating habits among students in the intervention group. The study is also expected to reduce weight, BMI, smoking and alcohol habits of the intervention group.

i

**TABLE OF CONTENTS**

**Page**

**ABSTRACT** i **LIST OF TABLES** v **LIST OF FIGURES** vi **LIST OF ABBREVIATIONS**  vii

**CHAPTER**

**1 INTRODUCTION** 1

| 1.1 | Background | 1 |
| --- | --- | --- |
| 1.2 | Problem Statement | 5 |
| 1.3 | Significance of Study | 6 |
| 1.4 | Research Question | 6 |
| 1.5 | Objectives of the Study | 7 |
|  | 1.5.1 General Objective | 7 |
|  | 1.5.2 Specific Objectives | 7 |
| 1.6 | Research Hypotheses | 7 |

**2 LITERATURE REVIEW** 9

2.1 Background Information on Literature Search Strategy 9

2.2 Education System in Ghana 9

2.3 Cardiovascular Diseases Definition and Types 10

2.4 Epidemiology of Cardiovascular Diseases 10

2.5 Risk Factors of Cardiovascular Diseases 12

2.6 Previous School Based Intervention Studies on Cardiovascular

Disease Risk Factors 12

2.6.1 Physical Inactivity 12

2.6.2 Smoking 16

2.6.3 Alcohol Consumption 18

2.6.3.1 Culture of Alcohol Consumption in Ghana 18

2.6.3.2 Previous School Based Study on Alcohol

Consumption 18

2.6.4 Unhealthy Diet 20

2.6.5 Obesity 22

2.6.6 Hypertension 25

2.7 Factors Associated with CVD Risk Factors 27

2.7.1 Age 27

2.7.2 Gender 27

2.7.3 Ethnicity/Race 28

2.7.4 Family History of Cardiovascular Diseases 28

2.8 School-Based Health Intervention Programmes 29

ii

2.9 The Use of the IMB as the Theoretical Framework to Develop

the Intervention 29

2.9.1 Assumptions 31

2.9.2 Information 32

2.9.3 Motivation 32

2.9.4 Behavioral skills 32

2.10 Sustainability of the School-Based Intervention Program in

Reducing Cardiovascular Risk Factors 33

2.11 Conceptual Framework of the Study 34

| **METHODOLOGY** | | | 36 |
| --- | --- | --- | --- |
| 3.1 | Study Location |  | 36 |
| 3.2 | Study Design |  | 37 |
| 3.3 | Study Duration |  | 38 |
| 3.4 | Sampling |  | 38 |
|  | 3.4.1 Study Population |  | 38 |
|  | 3.4.2 Sampling Population |  | 38 |
|  | 3.4.2.1 Inclusion Criteria |  | 38 |
|  | 3.4.2.2 Exclusion Criteria |  | 38 |
| 3.5 | Sampling Technique |  | 39 |

**3**

|  | | |  |
| --- | --- | --- | --- |
| 3.13.1 | | Validity of Questionnaire | 48 |
| 3.13.2 | | Face Validity | 49 |
| 3.13.3 | | Content Validity | 49 |
| 3.13.4 | | Validity of the Anthropometric Measurement | 49 |
| 3.13.5 | | Reliability of Questionnaire | 49 |
| 3.14 | Implementation of Intervention | | 50 |
| 3.15 | Variables | | 52 |
| 3.15.1 | | Independent Variable | 52 |
| 3.15.2 | | Dependent Variables | 52 |
| 3.15.3 | | Confounders | 52 |

|  | | |  |
| --- | --- | --- | --- |
|  | 3.5.1 Sampling Frame |  | 39 |
|  | 3.5.2 Sampling Method |  | 39 |
| 3.6 | Sample Size Estimation |  | 39 |
| 3.7 | Randomization |  | 40 |
|  | 3.7.1 Sequence Generation |  | 41 |
|  | 3.7.2 Allocation Concealment Mechanism |  | 41 |
|  | 3.7.3 Implementation |  | 41 |
| 3.8 | Blinding |  | 41 |
| 3.9 | Development of the School CVD Risk Factor | Reduction |  |
|  | Intervention Module for Secondary School Students |  | 41 |
|  | 3.9.1 Validation of the Module |  | 45 |
| 3.10 | Training |  | 45 |
| 3.11 | Intervention |  | 45 |
| 3.12 | Data Collection |  | 46 |
|  | 3.12.1 Study Instruments |  | 46 |
|  | 3.12.2 Questionnaire |  | 46 |
|  | 3.12.3 Anthropometry Measurements |  | 48 |
|  | 3.12.4 Blood Pressure Measurements |  | 48 |
| 3.13 | Quality Control of Study Instruments |  | 48 |

| 3.16 Operational Definition of Terms | | | | 52 |
| --- | --- | --- | --- | --- |
| 3.16.1 | | Body Mass Index |  | 52 |
| 3.16.2 | | Hypertension |  | 52 |
| 3.16.3 | | Physical Activity |  | 53 |
| 3.16.4 | | Smoking |  | 53 |
| 3.16.5 | | Alcohol |  | 53 |
| 3.16.6 | | Diet Practices |  | 53 |
| 3.16.7 | | CVD Risk Factor Knowledge |  | 53 |
| 3.16.8 | | Motivation |  | 53 |
| 3.16.9 | | Behavioral Skills |  | 54 |
| 3.17 Data An | | nalysis |  | 54 |
| 3.17.1 | | Normality Tests |  | 54 |
| 3.17.2 | | Descriptive Statistics |  | 54 |
| 3.17.3 | | Inferential Statistics |  | 54 |
| 3.17.4 | | Effect Size |  | 55 |
| 3.17.5 | | Sensitivity Analysis |  | 55 |
| 3.18 | Ethical Consideration | |  | 55 |

| **REFERENCES** | 56-79 |
| --- | --- |
| **APPENDICES** |  |
|  |  |
|  |  |
|  |  |
|  |  |

**LIST OF TABLES**

**Table Page**

2.1 Leading causes of inpatients deaths in districts and regional hospitals

in Ghana, 2003 and 2008 12

2.2 Prevalence of overweight and obesity among students 23

2.3 Prevalence of hypertension among students 26

3.1 Application of the Information-Motivation-Behavioral Skills Model

for school based CVD risk factor reduction intervention module 44

3.4 Summary of intervention module 51

**LIST OF FIGURES**

| **Figure**  2.1 | The Information-Motivation-Behavioral Skills Model | **Page**  31 |
| --- | --- | --- |
| 2.2 | Conceptual Framework | 35 |
| 3.2 | Steps Involved in the Development of the Intervention Module | 43 |
|  |  |  |

vi

**LIST OF ABBREVIATIONS**

BMI Body Mass Index

CDC Centre for Disease Control and Prevention

CHD Coronary Heart Disease

CONSORT Consolidated Standards of Reporting Trials

CVD Cardiovascular Disease

DALY Disability Adjusted Life Years

DBP Diastolic Blood Pressure

GDHS Ghana Demographic and Health Survey

GHS

GLMM

Ghana Health Service

Generalized Linear Mixed Model

GSS Ghana Statistical Service

IMB

IOM

Information-Motivation-Behavioral Skills

Institute of Medicine

ITT

MCAR

Intention-To-Treat

Missing Completely At Random

MOE Ministry of Education

MOH Ministry of Health

MVPA Moderate to Vigorous Physical Activity

NCD Non Communicable Disease

PA Physical Activity

PACTR

PAQ-A

Pan African Clinical Trials Registry

Physical Activity Questionnaire for Adolescents

PTA RCT

Parent Teacher Association

Randomized Controlled field Trial

SBP Systolic Blood Pressure WHF World Heart Federation WHO World Health Organization

viii

**CHAPTER 1**

**INTRODUCTION**

**1.1 Background**

Non communicable diseases (NCDs) are the number one public health challenge of the 21st century. There were 56 million worldwide deaths in 2012 where 38 million deaths representing 68% were caused by NCDs. More than 40% (16 million) of NCD deaths occur prematurely, affecting people below 70 years. Almost three quarters of all NCD deaths accounting for 28 million and majority of premature deaths, 82%, occurred in developing countries where most of the world’s population lives (WHO,

2011a; 2014). By 2020, NCD deaths are projected to almost equal the combined deaths from communicable, perinatal, maternal, and nutritional diseases in the African continent and to exceed the combined deaths by 2030 (WHO, 2011a). The NCDs are mainly cardiovascular diseases (CVDs), cancers, chronic respiratory diseases and diabetes (Hunter & Reddy, 2013; WHO, 2015a). Cardiovascular deaths accounts for almost half (46.2%) of all NCD deaths, followed by cancer deaths, 21.7%, with respiratory diseases causing 10.7%, and diabetes with 4% deaths (WHO*,* 2014).

Cardiovascular diseases caused more deaths annually than any other causes (Mozaffarian et al., 2015; WHO, 2015b; World Heart Federation, 2015; Smith et al., 2012). Globally, an estimated 17.5 million people died in 2012 due to CVDs, representing 31% of all global deaths (WHO, 2015b) and affected a third of adult population in the world making it the largest epidemic ever known to mankind (Yusuf, Wood, Ralston, & Reddy, 2015). In Ghana, cardiovascular diseases have been the

leading causes of all NCDs and hospital deaths in 2008 accounting for 14.5% of total deaths (Bosu, 2013). According to the Ghana Health Service (GHS, 2015), CVDs are

the leading causes of NCD deaths with an estimated 35,000 deaths per year. In a five-year review of autopsy cases (19,289) from 2006 to 2010 in one of the teaching hospitals in Ghana (Korle-Bu Teaching Hospital, KBTH) found out that more than one-fifth (22.2%) of the deaths were due to CVDs (Sanuade, Anarfi, Aikins, & Koram,

2014). Also CVDs rose from the seventh and tenth causes of death in the capital

(Accra) in 1953 and 1966 respectively to the number one cause of death in 1991 and

2001 and has continued as one of the major causes of death since then (Agyei-Mensah

& de-Graft Aikins, 2010). In 2014, stroke and coronary heart disease (CHD) were ranked as the 2nd and 4th leading causes of death in Ghana accounting for 9.75% and

6.48% of all deaths respectively (World Health Rankings, 2014). The WHO (2015c) has reported that NCDs accounted for 34% of total deaths and 31% disability adjusted life years in Ghana with CVDs being the leading cause of NCD deaths.

Risk factors of CVDs are of two types which are modifiable and non-modifiable factors. Non-modifiable risk factors include advancing age, male gender, black race– ethnic background, prior stroke/transient ischemic attack or history of coronary heart disease and family history of stroke. On the other hand, modifiable risk factors of

CVDs include physical inactivity, smoking, alcohol consumption, unhealthy diet, obesity, hypertension, and diabetes mellitus (World Heart Federation, 2017).

Physical inactivity is the fourth leading risk factor for mortality (WHO, 2010). An estimated 3.2 million deaths occur each year due to physical inactivity. In 2010, prevalence levels of physical inactivity in the Eastern Mediterranean region was the highest (88%) followed by both Africa (85%) and Western Pacific regions (85%). In

2010 globally, 23% of adults aged 18years were physically inactive as explained by doing less than 150 moderate-intensity physical activity per week or its equivalent (WHO, 2014). On the other hand, 81% adolescents between the ages of 11-17 years were physically inactive in 2010 as explained by doing less than 60 minutes of moderate to vigorous daily physical activity, as recommended by the WHO (WHO,

2014). People who are physically inactive have a greater risk for all-cause mortality as compared to those who do at least 30 minutes moderate physical activity on most days in the week. Engaging in 150 minutes of moderate physical activity each week reduces the risk of coronary heart diseases, stroke, hypertension, diabetes, depression, and cancers. Physical activity is a major determinant of energy expenditure and therefore fundamental to balance and weight control (WHO, 2010). A nationwide school based survey among adolescents in Ghana found out that proportion of secondary school students who reported being physically active all days for a total of at least 60 minutes per day during the past seven days were only 18.7% (MOH, 2012).

Tobacco use is one of the main causes of preventable deaths worldwide. Tobacco use is associated with cardiovascular diseases. There are currently about one billion smokers in the world. An estimated six trillion cigarettes are smoked annually by current smokers. Six million people die each year from tobacco use and exposure (WHO, 2011a). The GHS (2017a) reported that the prevalence of tobacco use in Ghana in 2014 was 5.1% among males and 0.4% for females. A research conducted among students of 11-17 years in Ghana revealed that prevalence of smoking among boys was 2.4% while girl smokers was 1.4% (Mamudu & Veeranki, 2013).

Alcohol consumption is associated with the risk of hypertension and hemorrhagic stroke. Globally, an estimated 3.3 million people died (5.9% of all worldwide deaths) in 2012 as a result of alcohol consumption. More than half of these deaths resulted in NCDs of which CVDs and diabetes accounted for 33% of these deaths. Globally, the levels of alcohol consumption in 2010 was estimated at 6.2 liters of pure alcohol per person ≥15 years of age (WHO, 2014). In Ghana, the levels of alcohol consumption in 2016 per person ≥15 years of age was estimated to be 2.7 liters (WHO, 2018a). In a research among 1,311 adolescents school students in Ghana found that prevalence of alcohol use among students was 42.3% (Hormenu, Hagan, & Schack, 2018).

Unhealthy diet accounts for 1.7 million deaths and 16 million DALYs worldwide as a result of low consumption of fruits and vegetables (WHO, 2014). Adequate fruits and vegetables intake reduces the risk of CVDs (Boeing et al., 2012). Consumption of high energy foods including processed foods like fats and sugars results in obesity (Mendonca et al., 2016). Also, high salt intake increases the risk of hypertension and

CVDs. Globally, the WHO has estimated that people consume between 9-12 g/day of salt which is far above the recommended intake of less than 5g/day. The WHO further indicated that saturated fat predisposes an individual to the risk of coronary heart diseases whereas monounsaturated and polyunsaturated reduces the risk (WHO, 2011a) while the consumption of fish is very low. A study in Ghana among secondary school students revealed the consumption of sweet snacks, sodas, and energy dense foods were high (Amoh & Appiah-Brempong, 2017).

Obesity has been a major problem in recent times. Globally, the prevalence of this risk factor has more than doubled since 1980 and 2014. There were 11% males and 15% females aged 18 years and older who were obese globally in 2014 (WHO, 2014). Thus half a billion adults worldwide are obese. Obesity is a major risk factor of diabetes, hypertension, coronary heart disease and stroke. Overweight and obesity are defined as BMI ≥25kg/m2 and ≥30kg/m2 respectively accounted for 3.4 million deaths in 2010 and 93.6 million Disability Adjusted Life Years (DALYs) (Cawley, Meyerhoefer, Biener, Hammer, & Wintfeld, 2015; WHO, 2014). The prevalence of childhood obesity is becoming a major challenge especially in low and middle income countries. This is as a result of overindulgence in consumption of high caloric foods, activities such as watching television, playing video games and the internet and other forms of physical inactivity (WHO, 2014). A nationwide study among secondary school students in Ghana reported that 8% of the students were either overweight or obese (MOH, 2012).

Hypertension is a major CVD risk factor (Chiolero, Bovet, & Paradis, 2013; WHO,

2014). The global prevalence of hypertension which is defined as systolic and/or diastolic blood pressure of ≥140/90 mmHg in adults aged ≥ 18 years was around 22% in 2014. The highest prevalence of hypertension across the WHO regions is in Africa, at 30% with the lowest prevalence in the region of the Americas at 18% (WHO, 2014). An estimate of 9.4 million people died worldwide in 2010 as a result of hypertension. The prevalence of hypertension in Ghana is about 48% as reported by the GHS (2017b) and has consistently ranked among the top ten causes of outpatient morbidity, admission, and death. The research was carried out in Ghana among 201 youth from three communities between the ages of 12-24 years found that 32.3% and 4% were pre-hypertensive and hypertensive respectively (Afrifa-Anane, Agyemang, Codjoe, Ogedegbe, & de-Graft Aikins, 2015).

The prevalence of diabetes has been increasing globally especially in low and middle income countries. This increase is largely due to modifiable risk factors such as physical inactivity, overweight, and obesity. The global prevalence of diabetes (defined as a fasting plasma glucose value ≥ 7.0mmol/L (126 mg/dl) or being on medication for raised blood sugar was estimated to be 9% in 2014. In 2012, diabetes killed 1.5 million people and 89 million DALYs (WHO*,* 2014). The prevalence of adult diabetes in Ghana is about 9% (GHS, 2017b).

Studies have shown that the risk factors for coronary heart disease and stroke begin in childhood that develops in adulthood (Juonala et al., 2010). This means that modifying risks factors and changing behavioral lifestyle for CVD should begin at the youthful age. Therefore, prevention of CVDs should be tackled right from an early age. Unfortunately, many people are not aware of CVD and its risk factors (Lao, Chan, Tong, & Chang, 2015) and because of this the disease burden keeps on rising. Also, merely educating the general public on CVD and its risk factors seems not to have achieved much. Therefore, adding intervention programmes to education especially for the youth on an ongoing process will equip them in protecting themselves from developing CVD disease in adulthood. Educating those who have already developed the disease should be ongoing so that they take the necessary steps in keeping the disease at its lowest levels and educate their families on the dangers of CVDs and its risk factors especially the youth.

School health interventions programmes have been shown to give consistent improvement on the general health status of students (Adab et al., 2015; He et al.,

2015) and that they are ideal places for health programmes (Khambalia, Dickinson, Hardey, Gill, & Baur, 2012; Ploeg, Maximova, McGavock, Davis, & Veugelers,

2014). The WHO (2011b) estimated that these modifiable risk factors cause 80% of

CVDs mainly physical inactivity, smoking, alcohol use, and unhealthy diet. Although cardiovascular diseases occur in middle and adult ages, the risk factors that cause the disease are mainly lifestyle behaviors that are learned during childhood and carried into adulthood.

Some behavioral studies have shown to result in some improvement in reducing the risk factors of CVDs. A school-based intervention to improve physical activity among secondary school students showed a statistically significant increase among the intervention group than the control group (Wang & Wang, 2018). A randomized control trial study was conducted to lower the intake of salt in students. The study found out that salt intake had decreased significantly in the intervention group and increased in the control group. The mean effect for intervention compared to the control group was -1.9 g/day (95% CI, -2.6 to -1.3 g/day; *p* < 0.001). Further, the systolic blood pressure showed a mean effect of -0.8 mmHg (He et al., 2015). Another lifestyle intervention study reported a significant reduction in BMI of students in the intervention group (-1.76kg/m2) whiles students in the control group showed an increase (1.13kg/m2) (Khumros, Vorayingyong, Suppapitiporn, Rattananupong, & Lohsoonthorn, 2019). A study showed significant reduction of smoking in the intervention arm as compared to the control arm (Gabrhelik et al., 2012). Another study reported that participants in the intervention group significantly increased consumption of fruits and vegetables when compared to the control group (Drapeau, Savard, Gallant, Nadeau, & Gagnon, 2016).

The main aim of this study is to evaluate the effectiveness of a school-based intervention programme on CVD risk factors which will be based on the Information- Motivation-Behavioral skills (IMB) model. Physical inactivity, smoking, alcohol, unhealthy diet, obesity, and hypertension will be the modifiable risk factors that will

be targeted for change among public secondary schools in the Brong Ahafo Region of Ghana.

**1.2 Problem Statement**

Cardiovascular disease (CVD) is the leading cause of death globally. The burden has been increasing in Ghana and has therefore become a major public health problem. In Ghana it is a leading cause of death since 2001 and has continued as one of the major causes of death since then. In 2008, cardiovascular diseases were responsible for

14.5% of all hospital deaths (Ghana MOH, 2012; GHS, 2015).

Despite all the CVD preventive risk factor measures in place in Ghana, the risk factors among adults have shown increasing trends over the years (MOH, 2012; GHS, 2015). Also, because the risk factors for the development of CVDs begin in childhood and are carried into adulthood, there should be an urgent need to educate students on the risk factors of CVDs right from childhood. Again, CVDs can be prevented if interventions that reduces the risk factors of the diseases are made available to people (Mendis et al., 2011) and that addressing a single modifiable risk factor still leaves one at a higher risk of developing CVDs because of failure in tackling the other coexistent risk factors. Also merely educating students on healthy lifestyles without interventions by reducing CVD risk factors may not be enough.

Therefore, to reduce this health and economic burden of the disease in Ghana, the prevalence of the disease and its risk factors among the youth and adults must be reduced drastically. In order to achieve this, preventive measures must start as soon as possible among students in schools in the country. This is because school health programs have shown to result in consistent improvement on the general health status of students. Two districts (Kintampo-North and Nkoranza-North districts) in the region were then selected for this study. This is because the educational levels in the two districts are low and accessibility to health information is poor. In this study, to the best of our knowledge, no behavioral modification intervention studies to reduce CVD risk factors among secondary school students in Ghana has been carried out. Since behavioral change still remains a driving force for reducing CVDs, there is therefore an urgent need for a behavioral change prevention intervention among secondary school students.

The IMB model is one of such psychological conceptualization for understanding and promoting health-related behavior. The IMB conceptualizations was developed (Fisher & Fisher, 1992) to address limitations found in other related theories. This included description of relationship among constructs, predictive validity of key constructs, conceptual parsimony and the inclusion of constructs that are needed for understanding and changing health behaviors. It was also designed to make it easy to be translated into other intervention programs such as addressing obesity-related behaviors, adherence to complex medication regimens, HIV preventive behaviors, and safety gear

utilization behaviors, among others (Fisher & Fisher, 2000; Fisher, Fisher, & Harman, 2003).

**1.3 Significance of Study**

This study will contribute to the body of knowledge on CVD risk factors among the secondary school students in the Brong Ahafo region of Ghana. Again, the intervention module if successful, can be adapted and incorporated by the Ghanaian Ministry of Education (MOE) into the school curricula program. Furthermore, the intervention study will inform, motivate, and give students the necessary skills that will enable them to practice healthy lifestyle behaviors such as doing at least 60 minutes moderate to vigorous physical activities daily, consumption of healthy foods such as fruits, vegetables, and seafood whilst reducing sugar and salt intake, as well as quitting or not initiating smoking and alcohol.

The IMB is a very powerful behavioral change tool that has been used over the years to improve and to sustain healthy lifestyle behaviors (Chang, Choi, Kim, & Song,

2014). The model had the potential in reducing CVD risk factors because is it composed of critical elements that were needed to adopt and maintain healthy behaviors. The constructs are based on social and health psychology theories that was developed to address limitations in social and health psychology theories such as the description of relationship among constructs, predictive validity of constructs, and the inclusion of constructs that are needed for understanding, changing, and sustaining healthy behaviors.

Also on sustainability of the intervention, students will be trained not to only reduce CVD risk factors alone, but to act as peer educators among their friends and in the home. This study will involve the participation of teachers and school authorities. Physical Education (PE) teachers will be trained by the researchers to implement the physical activity module during PE lessons while the other teachers will be trained on the health education module for continuous implementation after the intervention.

**1.4 Research Question**

The research question of this study is: what are the effects of the behavioral modification intervention program in reducing cardiovascular disease risk factors among secondary school students?

**1.5 Objectives of the Study**

**1.5.1 General Objective**

The general objective of this study is to develop, implement and evaluate the effectiveness of a behavioral modification intervention program to reduce cardiovascular disease risk factors among secondary school students in Brong Ahafo, Ghana.

**1.5.2 Specific Objectives**

The specific objectives of this study are:

1. To determine and compare the socio-demographic characteristics among intervention and control groups at baseline.

2. To determine and compare knowledge, motivation, behavioral skills and CVD risk factors (physical inactivity, smoking, alcohol consumption, unhealthy diet, BMI, diastolic and systolic blood pressures) between intervention and control groups at baseline.

3. To develop and implement a behavioral modification intervention program to reduce CVD risk factors among secondary school students in Brong Ahafo, Ghana.

4. To evaluate the effectiveness of behavioral modification intervention program to increase knowledge, motivation and behavioral skill on CVD risk factors between intervention group as compared to control groups at six months post intervention and also within groups from baseline to six months.

5. To evaluate the effectiveness of behavioral modification intervention program to reduce CVD risk factors in intervention group as compared to control group at six months and also within groups from baseline to six months among secondary school students in Brong Ahafo.

**1.6 Research Hypotheses**

The research hypotheses of this study are:

1. There will be no difference in the socio-demographic characteristics between the intervention and control groups at baseline.

2. The behavioral modification intervention program will be effective in improving the knowledge of CVD and its risk factors, motivation, and behavioral skill score among the participants in the intervention group as compared to control group and also within the groups from baseline to six months among secondary school students in Brong Ahafo, Ghana.

3. The behavioral modification intervention program will be effective in promoting physical activity levels of participants in the intervention group as compared to control group and also within the groups from baseline to six months among secondary school students in Brong Ahafo, Ghana.

4. The behavioral modification intervention program will be effective in reducing the prevalence of smoking among participants in the intervention group as compared to control group and also within the groups from baseline to six months among secondary school students in Brong Ahafo, Ghana.

5. The behavioral modification intervention program will be effective in reducing the prevalence of alcohol consumption of participants in the intervention group as compared to control group and also within the groups from baseline to six months among secondary school students in Brong Ahafo, Ghana.

6. The behavioral modification intervention program will be effective in promoting fruits and vegetable intake among participants in the intervention group as compared to control group and also within the groups from baseline to six months among secondary school students in Brong Ahafo, Ghana.

7. The behavioral modification intervention program will be effective in increasing seafood and water intake and reducing carbohydrates, fats and oils, fried eggs, fried chicken, carbonated drinks, plain sugar, sweet snack, and salted fish intake among participants in the intervention group compared to control group and also within the groups from baseline to six months among secondary school students in Brong Ahafo, Ghana.

8. The behavioral modification intervention program will be effective in reducing body weight and BMI of participants in the intervention group as compared to control group and also within the groups from baseline to six months among secondary school students in Brong Ahafo, Ghana.

9. The behavioral modification intervention program will be effective in reducing the mean diastolic and systolic blood pressures among participants in the intervention group as compared to control group and also within the groups from baseline to six months among secondary school students in Brong Ahafo, Ghana.

**CHAPTER 2**

**LITERATURE REVIEW**

**2.1 Background Information on Literature Search Strategy**

To conduct a review of cardiovascular disease risk factors among adolescents’ secondary school students, literature search will be performed for studies that will be relevant to CVD risk factors among students using PubMed, Google scholar, and Cochrane library. Strategy employ will be series of terms that will be used to identify articles of interest using the keyword search. The keywords will include: CVD risk factors among students (physical inactivity, smoking, alcohol, unhealthy diet overweight, obesity, and hypertension), epidemiology of CVDs, and primary prevention. Associations between demographic factors (age, gender, ethnicity/race, and family histories of CVDs) and CVDs will also be explored. The IMB module will be included in the search. With the exception of the IMB model (which work dated back in the early 1990s and 2000s), WHO standards for measurements, statistical formulae and its assumptions, searched articles will be included if they are published in the English language from 2010-2019. Studies will include randomized controlled trials, cohort studies, cross sectional and case control study designs that were conducted in schools and communities.

**2.2 Education System in Ghana**

The Ghanaian Ministry of Education (MOE) is responsible for the administration and the coordination of public education system in Ghana. The Ghana Education Service (GES) is the main agency responsible for the implementation of programs and policies of the MOE. The language of instruction in schools is English. The education system in Ghana is divided into three parts which are:

1. Basic education (kindergarten, primary school, and junior high school),

2. Secondary education (secondary school), and

3. Tertiary education (universities, polytechnics and colleges).

Basic education (age 2–13), is free and compulsory. This is divided into kindergarten (two years), primary school (six years) and junior high school (three years). The secondary school lasts for three years with students’ age range 14-19, which is form one to form three. Students in the secondary schools are either in the boarding school or as day students. Tertiary education is basically divided into university (academic education) and polytechnics (vocational education) (age 20-26). A bachelor's degree lasts for four years and can be followed by a one or two year master's degree. The student is then free to start a doctorate degree, usually completed in three to five years. A Polytechnic curriculum lasts two to three years. Ghana also possesses numerous

colleges of education. New tertiary education graduates have to serve one year within the national service Scheme (MOE, 2019).

**2.3 Cardiovascular Diseases Definition and Types**

The UK National Health Service, NHS, (2015) defines CVD**s** as a general term which describes a condition of the heart and blood vessels. Flow of blood to the brain, heart, or body is reduced as a result of blood clot (thrombosis) or by a buildup of fatty deposits inside the artery that causes it to harden and narrow in a condition called atherosclerosis.

The types of CVDs are:

(i) Coronary heart disease: a disease of the blood vessels supplying the heart muscle,

(ii) Cerebrovascular disease: a disease of the blood vessels supplying the brain,

(iii) Peripheral arterial disease: a disease of the blood vessels supplying the arms and legs,

(iv) Rheumatic heart disease: a damage to the heart muscles and heart valves from rheumatic fever caused by streptococcal bacteria,

(v) Congenital heart disease: a malformation of the heart structure existing at birth, and

(vi) Deep vein thrombosis and pulmonary embolism: blood clots in the leg veins, which can dislodge and move to the heart and lungs.

Heart attacks and strokes are usually acute events and are mainly caused by a blockage that prevents blood from flowing to the heart or brain. The most common reason is a build-up of fatty deposits on the inner walls of the blood vessels. Strokes can be caused by bleeding from a blood vessel in the brain or by blood clots (WHO, 2015d).

**2.4 Epidemiology of Cardiovascular Diseases**

Epidemiological studies play important role in explaining predisposing factors for CVDs and opportunities for prevention (Mahmood, Levy, Vasan, & Wang, 2014). The risk factors for the development of CVDs are not only peculiar to adults but these behaviors begin as early as in childhood and adolescents (Woodgate & Sigurdson,

2015). Cardiovascular diseases are currently the most leading causes of deaths worldwide with risk factors including hyperlipidemia, smoking, diabetes, and gender among others (Huang, Lee, Chang, Pang, & Chang, 2016) and has become epidemic in both developing and developed nations of the world which is deep rooted in majority of societies (Labarthe, 2011). The disease was recognized as common in developed countries in the 1960s and 1970s but have been halved since then as a result of better intervention strategies in both primary (lifestyle changes and risk factor control) and secondary (early detection and treatment) preventive strategies.

In comparison, CVD was considered to be a disease which was not found in developing countries in 1950s and 1960s but increased in the last three decades with more than

80% occurring in these countries (Yusuf et al., 2015). Globally the number of deaths due to CVDs rose by a third between 1990 and 2010 and in 2015, one in three deaths were due to CVDs (Mahmood et al., 2014).

It is also known that 30% of deaths in developing countries are attributable to CVDs. The Institute of Medicine (2010) also indicated similar trends that the disease was perceived to be of developed nations until recent years where epidemiological evidence has shown worsening of CVD health globally and more especially in developing nations. The worsening of CVDs has been associated with behavior factors such as childhood obesity, unhealthy dietary habits, increased smoking, and increased physical inactivity as a result of urbanization, globalization, and industrialization.

Also the epidemiological shift especially in developing countries has been associated with lifestyle changes and diets which is termed as epidemiologic and nutrition transition (Moran, Roth, Narula, & Mensah, 2014; Popkin & Slining, 2013). Consumption of energy dense diets that are high in sodium, fat and sugar coupled with low physical activity, sedentary lifestyle and tobacco smoking are major modifiable risk factors that cause more than 50% of all global cause mortality (Huang, Huang, Tian, Yang, & Gu, 2014; Yang et al., 2014; WHO, 2011a). Aside as being the leading cause of deaths worldwide (He, Pombo-rodrigues, & Macgregor, 2014; Lu et al.,

2014), CVD is also the leading cause of disability in the form of DALYs. In the year 1990, there were total loss of 85 million DALYs and is projected to reach approximately 150 million in 2020 (Perk, Helmut, Graham, Reiner, & Verschuren,

2012).

The epidemic is not different in Ghana as an estimate of 86,200 total deaths occur each year due to NCDs with 55.5% deaths under the age less than 70 years. Cardiovascular diseases are the leading cause of deaths in Ghana with an estimate of 35,000 or 40.6% of NCDs deaths (GHS, 2015; MOH, 2012). In 2003, CVDs were ranked as the 4th commonest hospital deaths, causing 8.9% of the total deaths in the country until 2008 when it was ranked as the number one cause of death or 14.5% of total deaths (MOH,

2012) as shown in Table 2.1. In comparison malaria dropped from number one cause of death (17.1%) to number two (13.4%) within the same period.

**Table 2.1 : Leading causes of inpatients deaths in districts and regional hospitals in Ghana, 2003 and 2008**

**Rank**

**2003**

**Cause of death Proportional mortality**

**Rank**

**2008**

**Cause of death Proportional mortality**

**rate (%) rate (%)**

| 1 | Malaria | 17.1 |  | 2 |  | Malaria |  | 13.4 |
| --- | --- | --- | --- | --- | --- | --- | --- | --- |
| 2 | Anaemia | 9.6 |  |  |  | HIV/AIDS |  | 7.4 |
| 3 | Pneumonia | 7.2 |  |  |  | Anaemia |  | 7.3 |
| 4 | Cardiovascular | 8.9 |  | 1 |  | Cardiovascular |  | 14.5 |
|  | disease |  |  |  |  | disease |  |  |
| 5 | Typhoid fever | 3.5 |  |  |  | Pneumonia |  | 6.2 |
| 6 | Diarrhoeal diseases | 3.5 |  |  |  | Septicaemia |  | 5.1 |
| 7 | Hepatitis | 3.2 |  |  |  | Meningitis |  | 2.3 |
| 8 | Meningitis | 3 |  |  |  | Diarrhoeal diseases |  | 2.3 |
| 9 | Septicaemia | 2.8 |  |  |  | All other causes |  | 41.5 |
|  | All other causes | 41.1 |  |  |  | Total |  | 100 |
|  | Total | 100 |  |  |  |  |  |  |

(Source: Ministry of Health Ghana, 2012)

**2.5 Risk Factors of Cardiovascular Diseases**

Balagopal et al. (2011) defined disease risk factors as a measurable biological characteristics of an individual that precede a well-defined outcome of that disease, predict that outcome, and are directly in the biological causal path. Major risk factors of CVD are physical inactivity, smoking, alcohol, unhealthy diet, overweight, obesity, and hypertension.

**2.6 Previous School Based Intervention Studies on Cardiovascular Disease**

**Risk Factors**

**2.6.1 Physical Inactivity**

Physical inactivity is the fourth leading risk factor for global mortality and a major implication for the prevalence of CVD and diabetes and their risk factors such as blood pressure, raised blood sugar, and overweight. Physical inactivity causes 27% of diabetes and 30% of CHD burden. Studies have shown associations between physical inactivity with cardiovascular diseases (Archer & Blair, 2011; Chomistek et al., 2013; Myers et al., 2015). A meta-analysis of prospective cohort studies have shown that high and moderate levels of physical activities reduces the risk of the incident of CHD and stroke between 20-30% in men and 10-20% in women (Li & Siegrist, 2012). Hills et al. (2015) have indicated in their study that the health benefits of physical activities included prevention of overweight and obesity, and improved cardiovascular health.

Studies have shown that majority of adolescents do not meet the minimum daily 60-minute physical activity (Colley et al., 2011; Hallal et al., 2012). It has been reported in studies that physical activity levels declines during childhood and the decline accelerates around the adolescent age (Dumith, Gigante, Domingues, & Kohl, 2011). A research was carried out across 34 countries in five WHO regions which included Ghana among 72845 school students with ages between 13 and 15 years to compare physical behavior and sedentary lifestyle. It was reported that majority of the students did not meet the physical activity recommendations and that sedentary lifestyle was high (Guthold, Cowan, Autenrieth, Kann, & Riley, 2010). Another study on 400 students between the ages of 6-14 years in Ghana revealed that 32% spent their leisure time playing video games or watching television (Mogre, Aniyire, & Gyamfi, 2013). Still in Ghana, a study among youth between the ages of 15-24 years in three communities found out that 84.1% of the participants were physically inactive (Afrifa- Anane et al., 2015).

It has been reported that school going adolescents who do some form of physical activities at least 60 minutes daily have a better general health status than their inactive counterparts including cardiovascular health, muscular endurance, reduced body fat, depression and anxiety (WHO, 2015a). Regular physical activity reduces the risk of heart diseases, stroke, and diabetes. Also regular physical activity leads to energy balance, weight control and prevention of obesity.

There are however a number of school-based interventions studies on physical activities. Some of the interventions studies were successful whereas some were not. A randomized control trial was conducted to evaluate the effects of a 12 week school based program to foster physical activity lifestyle of secondary school students between the ages of 12-17 years in Germany. A total of 1162 participated in the trial from 29 secondary schools. The main outcomes were out of school sports activities, moderate to vigorous physical activity, active commuting, doing chores and sedentary behavior. At the end of the study the secondary school students in the intervention group showed a significantly higher physical activity levels than students in the control group (Suchert, Isensee, Sargent, Weisser, & Hanewinkel, 2015).

Another randomized control trial was carried out in Australia on primary school students between the ages of 5-7 years to increase their physical activity levels. The intervention consisted of a 13-week playground intervention during break hours. Twelve (12) schools with a total of 221 were randomly allocated to either control (108) or intervention (113) groups. At the end of the study sedentary activity decreased significantly among students in the intervention than in the control group. Further, moderate to vigorous physical activity (MVPA) increased significantly by 12% among students in the intervention group as compared to their counterparts in the control arm of the study (Engelen et al., 2013).

A study was conducted to evaluate an eighth week Fit-4-Fun in an RCT trial. Four primary schools (213) were randomly allocated into control (108) and intervention schools (118). Students in the control group participated in their normal school

curriculum. Participants were assessed at baseline and at six months follow up. The primary outcome was cardio respiratory fitness (CRF) with BMI, muscular fitness, flexibility and physical activity as secondary outcomes. After intervention at six months, there were significant effects for CRF, BMI, flexibility, muscular fitness and flexibility (Eather, Morgan, & Lubans, 2013).

A systematic review and meta-analysis of RCTs to increase MVPA in school physical education lessons conducted by Lonsdale et al. (2013a) indicated that there were enough evidence that participating in MVPA could lead to a variety of health benefits for students. The researchers were of the view that school-based interventions studies could promote MVPA in students. In their analysis they reported that students in intervention group spent 24% more lesson time in MVPA as compared to students in the control groups and it was significant. The researchers concluded that physical education interventions could lead to significant public health benefits.

Another secondary school-based cluster randomized control trial was conducted with the aim to increase students’ physical activity and to motivate students in physical education. A total of 288 students with a mean age of 13.6 years from five secondary schools participated in the trial. Parents, teachers, school principals and physical education teachers provided written consent for the study. Data were collected at baseline and post intervention. Participating students were either assigned to the control arm or one of the three interventions arms namely, explaining relevance, providing choice, and complete free choice. The primary outcomes were however, accelerometer assessed physical activity and students’ motivation during lessons. Perception of teacher support, sedentary behavior, and psychological needs satisfaction accounted for the secondary outcomes. At the end of the study, free choice intervention significantly increased PA (*p* < 0.05) and decreased sedentary behavior (*p* < 0.05) (Lonsdale et al., 2013b).

Melnyk et al. (2013) conducted a study in the United State of America on healthy lifestyle on first and second year secondary school students between the ages of 14 and 17 years. A total of 779 students (female 401, 51% and males 378, 49%) were enrolled into the study from 2010 to 2012. The study was to test the efficacy of an intervention, COPE (creating opportunities for personal empowerment) healthy lifestyle TEEN (thinking, emotion, exercise and nutrition) program with a control programme called Healthy Teens. The intervention involved integration of 15-20 minutes physical activity (walking, dancing, and kick boxing) into a health course. The study hypothesized that students who receive the intervention would have healthy lifestyle behaviors and decreased BMI, including improved mental health, social skills and academic outcomes immediately following and at six months intervention than students in the control arm. Results at post intervention showed that students in the intervention arm had significantly greater number of steps per day (*p* = 0.03) and a lower BMI (*p* = 0.01) as compared to students in the control group and higher average scores on all social skills ratings (*p* < 0.05). Further, there were significant decrease of depression among students in the intervention than in the control group and

significantly higher health course grades of students in the intervention arm than did the control students.

A cluster RCT was undertaken to evaluate the impact of 12 month based multi

component program on students’ physical activity and sedentary behavior drawn from

12 secondary schools. The intervention, NEAT (nutrition and enjoyable activity for teen girls) program included lunch time physical activity sessions, enhanced school sports, interactive seminars, nutrition education, students’ handbooks, pedometers, and text messages as reminders to continue healthy lifestyles. A total of 357 students participated in the trial. Teachers delivered the sports and lunch time physical activity sessions, while dieticians delivered the nutrition sessions and the research team delivered the interactive educational seminars together with text messages. The results showed significant difference between the intervention arm for self-reported recreational computer use (-2.6 min; 95% CI -46.9 to -5.1) and summed sedentary activities (-56.4 min 95% CI -110.1 to -2.7). There was no significant difference between intervention and control on objective sedentary behavior (Dewar et al., 2014).

Brustio et al. (2018) conducted a four-month school-based intervention to explore the effect of motivation on physical activity. The study included a total of 276 students with a mean age of 13 years and were randomized into intervention (138) and control groups (138). The intervention included a motivation and a physical activity phase. The motivational phase was to encourage students to improve physical activity while a one kilometer daily walking every morning was conducted for the physical activity phase. At four months post intervention study, students in the intervention group recorded significant improvements in both motivation and physical activities.

Another secondary school-based intervention was carried out by Rezapour et al. (2016) to improve physical activity whiles decreasing sedentary lifestyles among students in the intervention group. Four schools were used in the study and were divided into intervention and control schools. The intervention schools went through a six-month physical education lessons and exercises which was delivered by school physical education teachers. Students in the intervention group were thought on simple exercises and were equipped with behavioral kills related to physical activities. At three and six months, students in the experimental group had significantly improved physical activities and a reduced sedentary behavior.

Guerra et al. (2013) conducted a meta-analysis of the effects of school based interventions of physical activities on BMI. The researchers used a total of 11 RCTs with a total of 4,273 which were studies that were published between April 2009 and September 2012. The results indicated there may be some beneficial effects from physical activity in the school environment. There were no statistically significant differences between the intervention and the control groups.

**2.6.2 Smoking**

Tobacco use is a well-established and important risk factor for cardiovascular diseases (Filion & Luepker, 2013; Gellert, Schottker, Muller, Holleczek, & Brenner 2013; Katsiki, Papadopoulou, Fachantidou, & Mikhailidis, 2013) and one of the main causes of preventable deaths worldwide (Bauer, Briss, Goodman, & Bowman 2014; Li et al.,

2016; Thomas, Baker, & Thomas, 2016). Tobacco is a leading cause of stroke, heart attack, and peripheral vascular disease. It has also been reported that mortality among smokers is two to three times higher than those who never smoked (Carter et al., 2015). A cross sectional survey of students (1805; 49% males, and 51% females) from some selected secondary schools in China revealed that 9% (162) of the students were smokers. However, a large number of the students 81% (1462) had never been taught about smoking and tobacco prevention in school (Xu et al., 2015a). The rate of tobacco smoking among 13-15 year olds is increasing and many more children are beginning to smoke at the age of 10 years (WHO, 2010).

Globally, smoking is five times higher in men (37%) than in women (7%) and cigarette accounts for 80% of all current smoking (WHO, 2014). The prevalence of tobacco smoke in Ghana was 10% in 2011. In 2010 in Ghana, tobacco smoke caused 2.7% and

1% male and female deaths respectively (Tobacco Atlas Country Report, 2015). A Global School-based Student Health Survey (GSHS) conducted across 75 Ghanaian secondary schools with a total of 7134 students reported that of the number that smoked, 45.4% tried their first cigarette smoking at age 13 years or younger (MOH,

2012). A research done in Ghana on 4289 people of 18 years and above reported that

15.6% and 5.4% men and women respectively were smokers (Thapa, Martinez, & Clausen, 2014).

Furthermore, some school-based RCT intervention studies on smoking have been successful whereas some had not. An RCT by Hiemstra et al. (2014) was conducted among elementary school children between the ages of 9-11 years. A total of 1478 students from 418 schools participated in the trial alongside their parents. An independent statistician randomized schools into intervention (728) and control (750) arms of the study and were followed for three years. The intervention, smoke free kids, concentrated on the prevention of smoking activities in families in order to prevent kids from smoking. The intervention module consisted of educational materials on smoking prevention that were sent to mothers via email. At the end of the study,

10.8% of the children in the intervention started smoking as compared to 12% in the control and there was no significance difference between the intervention and the control groups (odds ratio = 0.90, 95% CI, 0.63-1.27).

A randomized controlled trial was carried out to reduce the risk of smoking and alcohol use in a school setting among sixth graders. The intervention called ‘’unplugged’’ was delivered by trained teachers which focused on knowledge, attitudes, interpersonal skills, and intrapersonal skills over 45 minutes for a period of 12 months. The primary outcome was cigarette smoking patterns. Baseline and follow up data were taken at 1, 3, 12, 15, and 24 months with a total of 1874 students, and at

the final follow up students’ numbers dropped to 1753. The control on the other hand did not receive the intervention. However, there was a statistically significant effect for the final follow up for smoking and alcohol between the intervention and the control arm (Gabrhelik et al., 2012).

A school based intervention program Isensee et al. (2014) was carried out for 5th and

6th graders with the aim to prevent substance use. The intervention was delivered through life skills and that of specific skills which was tailored through substance abuse. This included coping with stress, problems, emotions, pressures to smoke and take alcohol. A total of 3444 from 45 schools took the baseline survey and 2513 (73%) took part in the post-intervention analysis. The prevention program was delivered in

90 minutes per session including two workshops. The control group went on with their normal school curriculum and did not receive any intervention. A baseline assessment was taken before the intervention and at post-tests intervention at six months follow

up. The results showed a smaller significant effect on lifetime smoking (d= 0.26) and smoking incidence (d = 0.23). There was however, no significant effect on current smoking.

Another study Guo et al. (2015) was conducted to prevent illicit drug, smoking and alcohol use among adolescents school children. At six and twelve months, there was a statistically significant reduction of illicit drug use among the intervention than the control group. A systematic review on tobacco use prevention and cessation was conducted on children and adolescents. In all, 19 trials were used for the analysis and the results from the random effects meta-analysis was 19% relative reduction with a risk ratio of 0.81 (95% CI; 0.70-0.93). The absolute risk difference was -0.02 (CI: -

0.03-0.00) in smoking initiation of adolescents in favor of the lifestyle behavior interventions compared with those in the control group (Patnode et al., 2013).

Gorini et al. (2014) conducted a randomized control trial to evaluate the effectiveness of an intervention to prevent smoking in secondary school children between the ages of 14-15 years. A total of 1646 students from 13 secondary schools participated in the baseline survey in the study that had randomized students into intervention (832) and control (814) groups. The prevention education program (intervention) consisted of out of school workshops, lessons on smoking prevention including anti-policies on smoking, and some peer led interventions. The primary outcome was daily and frequent smoking of cigarette. In the final analysis at 18 month follow-up, there was a statistically significant reduction (46%) of daily smoking in the intervention group as opposed to the control group.

**2.6.3 Alcohol Consumption**

**2.6.3.1 Culture of Alcohol Consumption in Ghana**

In Ghanaian societies, alcohol is mainly consumed by adults, while the youth are not allowed to take alcohol. Excess drinking and intoxication is generally viewed with extreme disapproval. The patterns of alcohol consumption are however reported to have changed due to sociocultural changes and the growth of alcohol industries and the youth have taken to drinking of alcohol. The type of alcoholic beverage consumed in many of the societies in Ghana include locally produced home brewed beverages with very high alcohol content and increased health and social consequences such as “akpeteshie” (WHO, 2018b; GSS, 2012).

Cultural background of the students may also play a role as they may have been exposed to alcohol consumption from their cultural backgrounds. Most cultures in Ghana use alcohol frequently for many functions (such as naming ceremonies) and students are exposed to these practices and tend to carry it on as a practice of their own. Advertisement of alcoholic drinks is also seen to play a major role in the consumption of alcohol by students. The alcohol producing companies win customers through attractive branding, persuasive advertisement and promotion. The alcoholic beverages are made to suit the demands of customers and these are also mentioned in advertisements to reinforce the interest of consumers (WHO, 2018b; Oppong Asante, Meyer-Weitz, & Petersen, 2014; Bosu, 2010).

Although alcohol are not supposed to be sold to children in Ghana like many other countries, but more often than not they get sold to children regardless of the policies in place. The reasons associated to alcohol use by children is mainly due to peer pressure (Elisaus et al., 2015) and the lack of knowledge of the harmful effects of alcohol amongst others. Therefore, one of the surest ways used in this study was to do primary prevention with the use of the IMB model to educate the intervention group on the dangers associated with alcohol and to encourage, and give some behavioral skills to stop or not to initiate alcohol.

**2.6.3.2 Previous School Based Study on Alcohol Consumption**

Harmful intake of alcohol alone caused 2.3 million deaths worldwide in 2003 which accounted for 3.8% deaths worldwide (WHO, 2011b) and the deaths rose to 3.3 million in 2012 (WHO, 2014). There is a direct relationship between high alcohol consumption and cardiovascular diseases (Holmes et al., 2014; Shakeshaft et al., 2014). According to Movva and Figueredo (2013), consumption of alcohol caused cardiac arrhythmias, cardiomyopathy, and hemorrhagic stroke. Ikehara et al. (2013) reported in their prospective study of 47,000 Japanese women that alcohol drinking was associated with high risk of stroke. Also, use of alcohol increases risk of hypertension (Briasoulis, Agarwal, & Messerli, 2012) which is a single risk factor for CVD.

In Ghana, total alcohol consumption per capita in 2010 was 4.8 liters; with males consuming 7.8 liters and 1.9 liters for females (WHO, 2015c). Further, 5.1% DALYs of the global burden of disease is attributed to alcohol consumption. A study conducted among 227 Ghanaians of age 8 years and older reported that 12% of the children take alcohol daily (Oppong Asante et al., 2014). In another survey conducted in Ghana among school going age adolescents revealed that the prevalence of current alcohol use was 12.6% (Oppong Asante & Kugbey, 2019).

School-based randomized control trials have been undertaken to reduce alcohol intake among students. An RCT trial was carried out to evaluate the effectiveness of a school- based alcohol harm prevention intervention among students between the ages of 13-

14 years for a period of nine months. A total of 21 schools (1746) participated in the trial which was made up of 14 schools (1161) in the intervention with 7 schools (585) in the control arm. Students in the intervention group received comprehensive focused drug minimization education which was conducted by teachers while the control group carried on with their normal drug education programme taught by schools. The main outcome was general knowledge of drug and alcohol consumption. There was a statistically significant increase in knowledge about drugs and alcohol (*p* < 0.001) among students in the intervention group as compared to the control group. There was no significant differences between the two groups in terms of alcohol consumption (Midford et al., 2014).

A meta-analysis of randomized control trials on effectiveness of school-based preventive interventions on students alcohol use was carried out by Strøm et al. (2014) of articles published from January 1990 to August 2015. A total of 28 (39289) randomized control trials studies were used in the meta-analysis. The researchers reported in their findings that the overall effect size was small but was positive among studies with the frequency and quantity of alcohol. There was however no effect found among studies that measured proportion of alcohol use.

Another cluster randomized control trial with two arms was conducted to prevent alcohol use among secondary school students. Students from 15 schools with ages from 13-15 years participated in the trial where seven schools with 343 students and eight schools with 356 students were randomly allocated into the intervention and control arms of the study respectively. The intervention consisted of a two 90 minute sessions for two weeks during school hours which consisted mainly on education in avoiding behaviors that led to alcohol consumption. The intervention education was carried out by three qualified counsellors and two facilitators. Students in the control arm received no intervention and went on with their normal school lessons. The main outcome measurement of the study was consumption of large quantities of alcohol at

12 months. The findings showed no statistically significant difference between rates of consumption in the intervention, 42.9%, and control, 49.2%, groups at 12 months follow up (Lammers et al., 2015).

Tripodi et al. (2010) conducted a meta-analytic review to assess the effectiveness of substance abuse interventions among students between the ages of 12-19 years to reduce alcohol intake. A total of 16 studies were used in the analysis. Frequency of alcohol use, quantity of alcohol use and abstinence were measured after post intervention between one month and a year in the study. The findings showed that interventions significantly reduced alcohol intake among the adolescents with a pooled effect of −0.61 (95% CI, −0.83 to −0.40 *p <* 0.001).

**2.6.4 Unhealthy Diet**

There is an association between dietary habits and cardiovascular risk factors such as overweight, obesity, diabetes, serum cholesterol, and blood pressure (Verschuren,

2012). Childhood obesity is a risk factor of CVD in adulthood, metabolic syndrome and development of arteriosclerosis. Cardiovascular risk factors and obesity affects vascular structure and function, and increased arterial stiffness (Herouvi, Karanasios, Karayianni, & Karavanaki 2013). Eating behaviors are central because the youth’s physical development, health, and identity are determined by factors such as knowledge, attitudes, sociodemographic characteristics, behavioral, and lifestyle factors and that behavioral patterns formed in adolescents are likely to influence long term behaviors (Abraham, Noriega, & Shin, 2018; Scaglioni et al., 2018; Sultana,

2017).

A study among adolescents reported high intake of sweet snacks and soft drinks while the consumption of fruits, vegetables, and fish was low (Gebremariam, Henjum, Terragni, & Torheim, 2016). A review among adolescents on dietary intake and sedentary behavior also found high consumption of fried foods including energy dense snacks and beverages with lower consumption of fruits and vegetables (Pearson & Biddle, 2011). In a study by Zaborskis et al. (2012) reported of high intake of sugar, carbonated drinks, pastries, chips and fast foods among school-aged adolescents. In another study, 33.8% of the students indicated they preferred carbonated drinks for snack (Buxton, 2014). Further, another study reported that consumption of fried and sugary foods among students were high (Alangea, Aryeetey, Gray, Laar, & Adamu,

2018). In a study on dietary salt intake reported that consumption of salt among participants was high (Menyanu et al., 2017). Furthermore, water intake among Ghanaian youth is inadequate (Doegah & Amoateng, 2018). Ministry of Health, Ghana (MOH, 2012) has indicted that adolescents have been consuming unhealthy foods that could lead to the onset of diseases during adulthood. This is because most students do not have knowledge on food and nutrition and are not aware of the repercussions of consuming unhealthy diets.

Healthy eating has shown to modify the risk of CVD. For example an overview of systematic reviews to examine diet behavior outcomes among populations with or at risk of NCDs was carried out. Fifteen (15) articles were used for the reviews. The results indicated that dietary behavior changes due to nutrition interventions were statistically significant in more than half of the interventions studies. Further, the results showed significant reductions in dietary fats while there were significant

increases in fruits and vegetables among the intervention group when compared to the control arm of the studies. There were also significant improvements in knowledge on nutrition in the intervention studies (Browne, Minozzi, Bellisario, Sweeney, & Susta,

2019).

A 12-month counselling intervention study was conducted to change the lifestyle of

53 people (age 40 years) who were at risk of cardiovascular disease. The intervention (counselling) was conducted through Skype or face to face interaction in a smaller group of about six study participants. The main outcome of the study was waist circumference, weight, BMI, blood pressure, blood glucose, cholesterol, reported and self-assessed lifestyle, and adherence to lifestyle changes. At 12 months, participants reported of improved and healthier diets. Overall the cardiovascular health had improved but it was not significant (Ylimaki, Kanste, Heikkinen, Bloigu, & Kyngas,

2015).

A number of school-based randomized controlled trials was done to improve healthy eating habits among students to decrease their cardiovascular risk. Cunha et al. (2013) conducted a school-based randomized control trial to prevent excessive weight gain among fifth graders with a mean age of 11 years old. The trial, code named PPPAS, meaning ‘’parents, students, and teachers for healthy eating’’. Twenty classes (ten each) from twenty schools were randomized into intervention and control groups. The intervention class received education relating to intake of fruits, water, beans and rice. Others included games, writing and drawing contest, reduction in the consumption of sugar sweetened beverages, cookies, and savory snacks in a nine nutrition education lessons given by trained nutritionist. The control group went on with their normal class activities. The main outcome of the study was BMI and body fat composition. There was no significant difference at the end of the study with regards to BMI (β = 0.003; *p*= 0.75) between the two groups. Students in the intervention group significantly increased consumption of fruits and decreased the intake of cookies and sweet snacks.

Another school-based RCT study trial called the CLICK-Obesity, which was a lifestyle and behavior intervention in addition to a routine health education practice was conducted to improve healthy eating habits among 4th grade primary school students. The intervention comprised of classroom activities including healthy eating and physical activities, school environment support, family involvement which included health classes and fun games. Written informed consent were obtained from parents/guardians and the schools. A total of 1182 (93.7% completed the study) students from eight schools were randomly allocated to either receive the intervention (4 schools) or to the control arm (4 schools). The control arm only received routine health education practice but not the intervention program. The main outcomes were BMI, behavior and lifestyle patterns and obesity related-knowledge. The students were followed for a year. There was a statistically significant reduction in the consumption of red meat, and decrease in the frequency of TV/computer use with an increase in jogging/running for students in the intervention arm than that of the control arm (Xu et al., 2015b).

An RCT trial was conducted to assess the impact of fruit and vegetable intake among middle school students. The sample size for the student population that participated in the trial was 1129 with 51.5% being females. Ten middle schools (5 each) were randomized into control and intervention groups. The intervention consisted mainly of health benefits of consuming recommended servings of fruits and vegetables. The results showed at the end of the study a statistically significant increase in the consumption of fruits and vegetable in the intervention group as compared to the control group (Baer, Jones, Mcclish, Westerberg, & Danish, 2012).

A two-year multi-component quasi-experimental design called FIT was utilized to improve dietary behavior and physical activity for 3rd, 4th and 5th graders. The school’s nutrition component included teachers being trained on nutrition to support the school’s nutrition education, healthy eating, and taste testing of healthy foods. At year one, the frequency of whole grain consumption in the intervention and control group doubled. In the second year, frequency of whole grain bread consumption in the intervention remained the same whereas those in the control group decreased from

0.77 times/day to 0.55 times/day and the difference between the two groups was statistically significant (Alaimo et al., 2015).

A non-randomized controlled trial done by Morano et al. (2016) among 18 overweight and obese students to reduce their BMI and to adapt a healthy eating habits for a period of six months. The intervention education consisted of nutrition education (including reduction in salt, sugar, and fat), a fun time physical activity, and other exercises. There was a significant reduction in general caloric intake (*p*<0.001), lower fat consumption (*p*< 0.001) and higher protein intake (*p*<0.001) in favor of the intervention group.

Another randomized control trial was conducted among 2348, 6th and 7th graders drawn from 40 middle schools to test the effectiveness of a multi-component school intervention for the prevention of non-communicable diseases. The intervention consisted of education and policies on healthy diets, physical activity, stop smoking and reaching out to the community/family. The intervention study lasted for eight months. The main outcome was self-reported measures on knowledge and behavioral changes on diet, smoking, and physical activities. Compared with the control arm at the end of the study, there was a statistically significance increase (10%; *p* < 0.01) in the proportion of students in the intervention group with regards to consumption of fruits (Saraf et al., 2015).

**2.6.5 Obesity**

The prevalence of obesity has reached epidemic levels and has become a major concern because it starts as early as in childhood (Bastien, Poirier, Lemieux, & Després, 2014) and is a major risk factor of CVD (Chrostowska, Szyndler, Hoffmann,

& Narkiewicz, 2013; Nakamura, Fuster, & Walsh, 2014; Schutter, Lavie, & Milani,

2014), diabetes (Cawley et al., 2015) high cholesterol and high blood pressure (Brady,

2016). Overweight children have a higher risk of diseases and premature mortality in adulthood (Black, Park, Gregson, Falconer, & White, 2015). Severe obese children are more prone to adverse cardio metabolic risk factor and shows early signs of vascular dysfunction and arteriosclerosis. A high BMI in childhood is associated with risk of CVD, diabetes and premature death (Kelly et al., 2013). Childhood obesity increases the risk of poor mental and physical health which leads to poor physiological and psychological health risk in adulthood (Robertson, Murphy, & Johnson, 2016). Studies have shown that adults who are obese have a 50% to 70% risk of developing cardiovascular diseases (DeBoer, 2013). There is evidence to suggests that lifestyle interventions on modifiable risk factors may prevent the onset of diabetes and future risk of obesity (Obirikorang et al., 2016).

Body mass index, which is weight in kg divided by height in meter squared (Kg/m2) is a measure of overweight and obesity (WHO, 2011b). Studies conducted on prevalence of overweight and obesity is shown in Table 2.2.

**Table 2.2 : Prevalence of overweight and obesity among students**

**Prevalence (%)**

| **Obesity** | **Overweight** | **Year of** | | **Place** |  | **Author** |
| --- | --- | --- | --- | --- | --- | --- |
| **publication** | | | | | | |
| 4.4 | 10.3 | 2017 |  | Ghana |  | Aryeetey et al. |
| 0.8 | 12.2 | 2015 |  | Ghana |  | Kumah et al. |
| 2.9 | 11.7 | 2013 |  | Ghana |  | Nyawornota et al. |

A number of school-based randomized control studies have been conducted. Some of which were: A meta-analysis of school-based randomized control trial was carried out to find the impact of long term physical activity interventions on BMI. A total of 18 papers which included 22381 primary school students of work published between

1990 and 2015 were used in the analysis. The findings from the study compared to the control showed a reduction in BMI (-2.23 kg/m) which was statistically significant (*p*

< 0.05) (Mei et al., 2016).

Lavelle et al. (2012) conducted a meta-analysis and systematic review on school-based interventions that sought to reduce BMI of students who were below 18 years. A total of 43 published studies were used for the review. Physical activity comprised 26% (11) of the interventions studies while education alone was 7% (3) and nutrition with a combination of the two were 67% (29). The results of the review and meta-analysis showed statistically significant difference of a reduction in BMI 0.17 (95% CI: 0.08 –

0.26. *p* < 0.001). This showed that intervention with two or more intervention strategies may be effective in addressing cardiovascular disease prevention.

A school-based randomized control trial was conducted to evaluate the effect of a 20 week exercise and healthy eating interventions on BMI in overweight students of age

7-10 years. Inclusion criteria were children with BMI above 90% for overweight and

those above 99% percentiles for obesity. The primary measurement was reduction in BMI with insulin sensitivity, body fat content, and metabolic syndrome as the secondary outcomes. Exclusion criteria included children below the ages of six and above ten years, who may be physically or mentally handicapped and may be on medication within the previous three months and somatic illness as cause for obesity. A total number of 37 students were then randomly allocated to the intervention (18) and the control (19) arms. The intervention consisted (i) weekly group training sessions which lasted for 60 minutes (ii) a 90 minute training sessions together with parents and siblings (iii) training of students and parents on healthy diet and nutrition, and (iv) cooking and dinning with parents. The results from the study showed a statistically significance reduction in the intervention group on BMI, -2.0 kg/m2 (95% CI: -2.5; -1.5, *p* < 0.001), fat mass -3.3 kg (95% CI: -4.2; -2.7, *p* < 0.001) and total body mass, -4.0 kg (95% CI: -4.9; -3.0, *p* < 0.001) when compared to the control group of the study (Harder-lauridsen, Birk, Ried-larsen, Juul, & Andersen, 2014).

Kelley et al. (2015) conducted a systematic review and trial sequential meta-analysis on school-based randomized control trials to determine the effects of exercise on BMI among overweight and obese students of ages 2 to 18 years. The primary outcome of the study was changes in BMI. The researchers selected a total of 5436 of published articles from 1990 up to 2014 and subsequently screened up to 20 articles which consisted of 971 students and 42 groups (20 control, 22 exercise). The pooled results showed a statistically significant decrease in BMI by 3.6% (-1.08; 95% CI -0.52 to -

1.64).

Another meta-analysis by Hung et al. (2015) was conducted to evaluate the effectiveness of school-based obesity prevention programs with a total of 26114 students of 6-18 years from 27 RCT studies. The intervention studies were based on physical activities and education. Schools that reported outcomes measures of BMI or skin fold thickness (which was the primary outcome of the study) and were of school- based intervention prevention studies were included in the analysis while cross sectional studies were excluded from the study. The results from the study showed a significant small effect size of 0.003 (95% CI; -0.013 to 0.092).

A CrossFit Teens randomized control trial intervention study by Eather, Morgan, & Lubans (2016) was carried out in a secondary school among grade ten students between the ages of 15 and 16 years in Australia. A total of 96 students were randomized into control (45) and intervention (51) groups with randomization occurring at the class level. The CrossFit intervention programme was delivered by two CrossFit instructors. The intervention constituted group exercises including push- ups, deadlift, and squat jumps within their limit and delivered twice a week over an 8- week period during school’s normal physical education period. The control group on the other hand participated in a 60 minute sport lessons like ice skating, tennis or sports and their usual physical education lessons delivered by their physical education tutor. The main outcome measurements were BMI and waist circumference. There was a statistically significant reduction in BMI (-1.38 kg/m2, *p* < 0.001) and waist circumference (- 3.1cm, *p* < 0.001) in the intervention when compared to the control.

A cluster randomized control trial was carried out in a school among 1st and 3rd grade students between the ages of six and eight years to evaluate the effectiveness of a 12- month multicomponent obesity prevention. A sample size of 1474 students from nine primary schools were randomly allocated into five intervention schools (651) and four control (823) schools. Primary outcome measures were obesity prevalence and BMI z score while students’ knowledge on healthy eating habits, and food types were measured. Intervention activities included improving sedentary life, nutrition and physical education delivered by trained teachers. The results from the study showed no significant difference between the intervention and control groups in terms of BMI z score and obesity prevalence (Kain, Concha, Moreno, & Leyton, 2014).

**2.6.6 Hypertension**

Hypertension is a major risk factor of CVDs (Gooding, Mcginty, Richmond, Gillman,

& Field, 2014; Kuciene & Dulskiene, 2014) and controlling hypertension reduces morbidity and mortality of CVDs (Falaschetti, Mindell, Knot, & Poulter, 2014). Risk of CVD doubles for each increase of 20/10 mmHg of blood pressure as low as 115/75 mmHg (WHO, 2011b). It is recommended that blood pressure should be kept below

140/90 mmHg. At 30 years for people with high blood pressure, the lifetime risk of developing CVDs is 63.3% compared to 46.1% people with normal blood pressure and the disease is developed at five years earlier (Rapsomaniki et al., 2014). Hypertension prevalence is expected to grow to more than 500 million by 2025 (Boateng, Luginaah, & Taabazuing, 2015).

Hypertension is a well-established and a leading risk factor for stroke and coronary heart disease (Okubo, Sairenchi, Irie, & Yamagishi, 2014; Ato et al., 2013) and one of the most important contributors of morbidity and mortality in the world (Fortuna et al., 2015). A study by National Health and Nutrition Examination Survey (NHANES) found out that 63.7% and 42.6% overweight and obese individuals respectively were hypertensive (Gaal & Maggioni, 2013). Consumption of too much salt is associated with hypertension and a major risk factor for CVDs (Brown et al., 2013). Studies have identified raised blood level as the best measure of CVDs especially stroke. Whelton (2015) indicated in his study that non-pharmacologic treatment of raised blood pressure had been reduction in dietary sodium.

Prevalence of hypertension has been increasing among children and that it persists into adulthood (Spagnolo et al., 2013; Thompson, Dana, Bougatsos, Blazina, & Norris,

2013). The number of reported new cases in Ghana between 1988 and 2007 rose by more than 1000% (Ofori-Asenso & Garcia, 2015). Some studies have reported prevalence of hypertension among students and adolescents. Table 2.3 shows prevalence of hypertension among students in Ghana and elsewhere.

**Table 2.3 : Prevalence of hypertension among students**

| **Prevalence (%)** | **Year of publication** | **Place** | **Author** |
| --- | --- | --- | --- |
| 13 | 2018 | Ghana | Sanuade et al. |
| 3.2 | 2015 | Ghana | Ghana Health Service |
| 28.3 | 2014 | Ghana | Awuah et al. |
| 16.6 | 2014 | UAE | Abdulle et al. |
| 8.5 | 2013 | Turkey | Demirci et al. |
| 5.0 | 2013 | USA | Thompson et al. |

A number of studies have been conducted to confirm the growing evidence that hypertension has its roots from childhood into adulthood. Also research conducted have found out that lifestyle behaviors like unhealthy diet, physical inactivity, sedentary life among adolescents significantly contributes to hypertension and CVDs in adulthood (Shrestha & Copenhaver, 2015). Therefore, it is important to prevent the various risk factors that predisposes children and adolescents to hypertension. A cohort study was conducted to identify different systolic blood pressure from childhood, their correlated risk factors, and early mid-life of developing cardiovascular outcomes of 975 participants. Blood pressure was taken at ages 7, 11,

18, 26, 32, and 38. It was found out that 21.8% had normal blood pressure, 43.3% were high-normal, 31.6% were pre-hypertensive and 4.2% were hypertensive. The research concluded that higher BMI and cigarette smoking resulted in significantly increasing blood pressure across the group and particularly for the higher blood pressure groups. Again, the pre-hypertensive and hypertensive groups had worse CVDs outcomes by early middle life (Reremoana et al., 2015).

A school-based HEROES (Healthy, Energy, Ready, Outstanding, Energetic, Schools) initiative was conducted by Kim et al. (2014) to prevent obesity which was based on a CDC (Centers for Disease Control and Prevention) school health programme to prevent blood pressure and the establishment of long term predictors of systolic and diastolic blood pressures. The other objective of HEROES was to improve healthy lifestyles among students, their families, and teachers. The intervention at the school focused primarily on physical activity and healthy eating. The intervention was analyzed at baseline, 6, 12, and 18 months. A total of 847 students from three high schools participated in the study. The results of the study showed a significant decrease in the prevalence of hypertension from baseline (17.1%) to 6 months (12.8%; *p* <

0.001), to 12 months (12.0%; *p* < 0.001), and 18 months (15.0%; *p* = 0.0024) in the intervention group when compared to the control group. High and frequent consumption of chips and fast foods predicted systolic blood pressure changes alone without diastolic blood pressure.

A systematic review and meta-analysis was conducted on the effect of childhood obesity prevention on blood pressure. Database were searched up to April 2013, which included randomized control trials, quasi experimental studies and natural experiments. The results showed a pooled effect of -1.64 mmHg (95% CI: -2.56, -

0.71; *p* = 0.001) for systolic blood pressure and -1.44 (95% CI: -2.28, - 0.60; *p*= 0.001)

for diastolic blood pressure. Also both physical activity and diet interventions showed

a greater significant reduction in both systolic and diastolic blood pressures than either physical activity or diet intervention alone (Cai et al., 2014).

Studies have also shown an association between dietary sodium intake and hypertension among children and adolescents. A study was conducted among 6235 students of 8-18 years old from the NHANES study between 2003-2008 to find the association of dietary sodium intake and hypertension. Multiple 24-hour dietary recalls were used to estimate sodium intake. The results showed at the end of the study that participants consumed an average of 3387 mg/day of sodium. An increased standard deviation score of 0.097mmHg systolic blood pressure was associated with a

1000 mg/day intake of sodium (Yang et al., 2012).

**2.7 Factors Associated with CVD Risk Factors**

**2.7.1 Age**

The aging process is a major determinant of developing CVD and is associated with deterioration in both structure and function of the heart and the vascular system (Sung

& Dyck, 2012). Age is a risk factor for CVD (Dhingra & Vasan, 2012) and the risk of developing CVD increases as one ages (Muka et al., 2016). It is an important and independent risk factor for CHD (Soler & Ruiz, 2010). The risk of getting stroke doubles at every decade after the age of 55 years (WHF, 2015). In the course of aging, large arteries dilate and their walls become thickened and the media exhibits collagen increase and frayed elastin (Jakovljevic, 2017). A population based health survey was carried out in Uganda among 1656 individuals with ages between 35-60 years. The prevalence of overweight and hypertension among the study population was 18% and

20.5% respectively and the study found out that increasing age was associated with being overweight (OR 1.8; 95% CI 1.12–2.79) and hypertensive (OR 4.5; 95% CI

2.94–6.96) (Mayega et al., 2012). Another study in Turkey among 1000 children between the ages of 6-13 years reported that 8.5% of the children were hypertensive while 11.2% were obese. The study further reported that BMI and blood pressure also significantly increased with increasing age (Demirci et al., 2013).

**2.7.2 Gender**

Gender is a non-modifiable risk factor for CVD. There is also a positive association between gender and CHD (Tabei, Senemar, Saffari, Ahmadi, & Haqparast, 2014). For some time now CHD has been regarded as a disease affecting mostly men and this led to women not being included in clinical diagnostic, prognostic, and therapeutic studies (Ruijter & Pasterkamp, 2015). Men are at greater risk of developing CVD than women at pre-menopausal stage than men but once women get past this stage they become similar with men which is due to loss of female sex hormones at the time of menopause (Barrett-Connor, 2013; Pérez-López, Larrad-Mur, Kallen, Chedraui, & Taylor, 2011). Maas & Appelman (2010) reported that CVD develops between 7-10 years later in females than in males and it may be as a result of perceived protection of estrogen in

reproductive life which delays the onset of arteriosclerosis in women. Psychosocial variables which are risk factors of CVD includes stress, depression, abuse, and domestic violence and post traumatic disorders. However, these depression disorders affect women heavily as compared to men (Wenger, Ouyang, Miller, & Merz, 2016). A study was conducted to measure calcium volume levels in different chest beds in elderly men and women with severe aortic stenosis. The results showed a significant gender total mean difference of calcium on coronary artery with men having the greatest levels of calcification. The same was for aortic valves calcium with men having the significant higher levels (Liyanage et al., 2016).

**2.7.3 Ethnicity/Race**

There are some variations when it comes to CVD in terms of ethnicity or race. For example, in the United States of America (USA) in 2013, the black race were 30% higher to die from CVDs than the white race and 113% higher mortality as compared to Asians and Pacific Islanders (Singh, Siahpush, Azuine, & Williams, 2015). Studies have also shown disparities in terms of CVD risk factors and ethnicity (Muncan,

2018). For instance, South Asians are predisposed to developing diabetes, while Ghanaians have lower prevalence of smoking, but have higher prevalence of hypertension compared with other ethnic groups (Snijder et al., 2017; Agyemang et al., 2016; Shrivastava & Misra, 2015). A study conducted by NHANES between 2009 and 2010 found out that Hispanic males have higher levels of cholesterol as compared to blacks and whites (Mozaffarian et al., 2015). Again higher levels of HDL are more likely to be found in blacks than in white ethnic groups (Yu, Castillo, Courville, & Sumner, 2012).

A study reported that ethnic minorities were less likely to engage in healthy behaviors like exercise and dietary practices than European-Americans especially in the middle age (August & Sorkin, 2011). Another study found that prevalence of obesity was higher in African-Americans than Asian-Americans and that African-Americans and Hispanics were more likely to engage in sedentary behaviors than European- Americans (Mozaffarian et al., 2015; Owen, Healy, Matthews, & Dunstan, 2012). Again relationship between race and smoking varies by country and gender (Mozaffarian et al., 2015). For example Bangladeshi men are more likely to smoke everyday than white English and white women are more likely to smoke than all other women of ethnic groups (Jamal et al., 2015).

**2.7.4 Family History of Cardiovascular Diseases**

There is association between family history and CVD. It has been reported that family history of CVD is an independent risk factor for CVDs (Nielsen et al., 2013). An individual is at a high risk of developing a heart attack and stroke if a parent or sibling has suffered a heart attack or stroke in their middle age life and the same goes for family history of hypertension, abnormal blood lipids and type-2 diabetes (WHF,

2015). Siblings of patients with CVDs have a 40% risk, whereas offspring of parents with premature CVD also have between 60%-75% risk increase (Kolber, 2014).

**2.8 School-Based Health Intervention Programmes**

The school health interventions are effective in addressing chronic disease prevention and more especially when they target multiple behavioral and environmental components with a complementary community strategy. Such schools are known globally as an all-round approach to support prevention and sustainability of lifestyle behaviors like physical activity, healthy eating, and smoking (Mcisaac, Hernandez, Kirk, & Curran, 2016). The adolescents phase presents opportunities for health and this pattern determines the future (adulthood) health status of the individual (Sawyer et al., 2012). Schools therefore can play a major role in improving the health status of students and adolescents and the adults they will become. Students are in school throughout the academic year and spend generally five days a week in school. This is because at this crucial adolescent age, students learn certain habits that influence behaviors such as physical activities, tobacco smoking, eating habits and alcohol use that could lead to chronic diseases like cardiovascular diseases, cancers, and diabetes which are now the leading causes of global death (Franks et al., 2015).

Domitrovich et al. (2016) reported in their study that school-based behavior and social learning interventions have positive impacts on students’ outcomes such as on substance use. The WHO (2016) has indicated that school health interventions have the benefit of improving health and preventing health related risk among the youth. Chen et al. (2015) reported that school-based intervention studies provide access to a large number of school children, who are often from different socioeconomic backgrounds and serves as opportunities to establish programs in communities.

Studies have also shown that CVDs affects people between the ages of 30-70 years but the risk factors begin in childhood and carried through adulthood (Eaton, Kann, Kinchen, Shanklin, & Ross, 2010; Skinner & Skelton, 2014). School-based NCD prevention interventions are important since they are key settings to improve health (Moore, Littlecott, Fletcher, Hewitt, & Murphy, 2016). Studies have also shown that school-based interventions are effective on CVDs especially in improving and sustaining physical activity, decreasing the consumption of unhealthy diets, and preventing obesity. Again, it has been reported that students could impact CVD knowledge to their parents and the whole family (Gunawardena, Kurotani, Indrawansa, Nonaka, & Mizoue, 2016).

**2.9 The Use of the IMB as the Theoretical Framework to Develop the**

**Intervention**

Theories are useful tools that help researchers accomplish many important outcomes and objectives in both academic and in the research field of study.

Theories help to:

1. Organize thoughts and ideas about the world

2. Generate and explain relationships and interrelationships among individuals, groups, and entities

3. Improve predictions and expectations about people, group, and organizations, and

4. Achieve better understanding of the world (Hambrick, 2007).

Whetten (1989) had indicated that a good theory should contain four essential elements which are: 1. What 2. How 3. Why and 4 Who, Where and When. A number of theories have been developed over the past centuries to understand and modify behavior. Some of these include the Trans theoretical Model, AIDS risk reduction model, Social Cognitive Theory, Theory of Planned Behavior, Theory of Reasoned Action and the Information-Motivation-Behavior Skills model.

The Information-Motivation-Behavioral Skills Model (IMB) is a well validated and comprehensive social psychological model for understanding and promoting health- related behavior. The IMB model was originally developed to predict HIV risk and preventive behavior. The constructs selected were based on two factors which were:

1. Critical review and integration of other social and health psychological theories, and

2. Analysis of different HIV interventions

The model was afterwards used in other health research which included diabetes self- care, motorcycle safety gear utilization, performance of breast self-examinations (Fisher et al., 2003), and tuberculosis infection control (Kanjee et al., 2012).

The IMB was based on a critical review and integration of constructs of behavioral theories, which contain key factors that are needed for modification of a wide range of health-related behaviors. Information, motivation and behavioral skills are factors that are associated with health-related behavior and are often addressed separately in health promotion interventions. The model however, postulates a causal relationship among the three factors which includes procedures that can be used to translate the model into health promotion interventions (Fisher et al., 2003). The model explains that complex behaviors require that individuals are well-informed, well-motivated, and have the necessary objective and perceived skills to engage in the complex behaviors (Fisher & Fisher, 1992; Fisher et al., 2003).

According to the IMB model, information and motivation constructs can be either independent or interrelated. Individuals who are well informed may not be motivated to perform a health-related behavioral change. Again, highly motivated individuals

may not be well informed about health promotion practices. Figure 2:1 describes the information, motivation and behavioral skills constructs. According to the IMB model, information and motivation may have direct effects on behavioral change if complicated or new skills are not required to perform the desired behavior. For example, pregnant women who are HIV positive might be willing to adhere to an anti- retroviral medication simply by learning this medication can prevent HIV transmission to their babies. Another example is an individual maintaining a sexually abstinent behavior because he or she is highly motivated (Fisher et al., 2003).

The IMB also assumes behavior is influenced by information and motivation through behavioral skills. In other words, the initiation and maintenance of behavioral change happen when health promotion behavioral skills are added to the health promotion information and motivation components. For example, individuals who are well informed about HIV facts, have personal and social motivation to perform HIV preventive behaviors, and apply necessary behavioral skills, are expected to initiate and maintain HIV preventive behaviors (Fisher et al., 2003).

**Figure 2.1 : The Information-Motivation-Behavioral Skills Model**


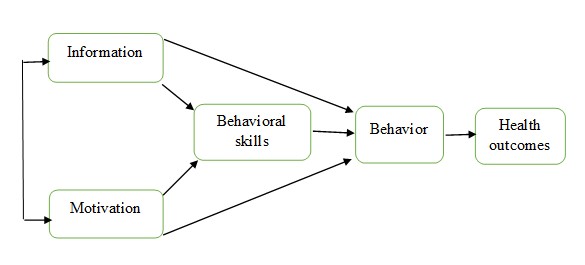


(Fisher and Fisher, 1992)

**2.9.1 Assumptions**

The IMB model assumes that health-related information, motivation, and behavioral skills are necessary to adopt health behaviors. Individuals who are well informed, motivated to act, and have the fundamental skills to perform a behavior, are very likely to adopt healthy behaviors and obtain beneficial health outcomes. On the other hand, individuals who are not well informed, are not motivated to act, and do not possess the skills needed to perform a behavior, are very likely to engage in risky behaviors and thus experience unfavorable health outcomes (Fisher & Fisher, 1992; Fisher et al.,

2003).

**2.9.2 Information**

Facts, heuristics, and implicit theories are different sources of information that could influence the performance of health behavior. According to the IMB model, information that is directly relevant to the performance of behavior and can reasonably be accomplished within an individual’s social ecology, is necessary to perform health behaviors (Fisher et al., 2003). For example, to promote HIV prevention, an intervention program would include: 1) the fact that condoms prevent HIV transmission, 2) the heuristic information about monogamous sex being safe, and 3) the implicit theory that “known and trusted people who dress and act reasonably and who possess a variety of normative characteristics are safe partners” (Fisher et al.,

2003). The three pieces of information mentioned above have been found to produce a powerful effect in the adoption of HIV preventive behaviors (Hammer, Fisher, Fiztgerald, & Fisher, 1996; Misovich, Fisher, & Fisher, 1996; Williams et al., 1992).

**2.9.3 Motivation**

Motivation determines the performance of a behavior by influencing individuals’ willingness to comply with the health promotion information given. According to the IMB model, personal and social motivations are two critical elements that influence the performance of health-related behaviors. Personal motivation is the attitude of an individual towards the health-related behaviors. Social motivation happens when there is social support that facilitates the performance of health-related behaviors. For example, personal attitudes towards condom use and perceptions of social support strongly influence whether individuals use condoms or not (Albarracin, Johnson, Fishbein, & Muellerleile, 2001). Similarly, behaviors in other health areas such as adherence to medications and performing breast self-examinations, can also be predicted by social support and personal attitudes (Champion, 1990; Fisher, Sand, Lewis, & Boroditsky, 2000; Lierman, Kasprzyk, & Benoliel, 1991; Misovich, Martinez, Fisher, Bryan, & Catapano, 2003).

**2.9.4 Behavioral skills**

In addition to information and motivation, behavioral skills provide the capability for individuals to perform health-related behaviors. This construct of the IMB emphasizes objective abilities and perceived self-efficacy associated with performing the desired behaviors. Again in HIV, the likelihood of using condoms is higher when an individual knows how to negotiate with a partner about condom usage. Many studies support the essentiality of self-efficacy to perform diverse health-related behaviors. For example, smoking cessation, breast and testicular self-examination, and medication adherence (Fisher et al., 2003; Glanz, Rimer, & Viswanath, 2008).

The constructs of the IMB model and their relationships are considered “highly” generalizable across populations and diverse health domains. However, this model assumes the content of each construct will be specific to relevant population’s health-

related behaviors. The content of constructs for an HIV prevention program will vary depending on the unique characteristics of individuals; for example, men or women and heterosexual or homosexual. According to this model, not every construct or causal pathway will have the same level of influence on health behaviors of diverse populations. The strength of each construct and causal pathways in influencing health behaviors will depend on the target population and the particular health behavior. The IMB model provides procedures that can be followed to identify constructs and causal pathways that are particularly influential for certain populations and health behaviors; this identification is crucial before developing targeted health promotion programs (Fisher et al., 2003).

**2.10 Sustainability of the School-Based Intervention Program in Reducing**

**Cardiovascular Risk Factors**

The sustainability of intervention programs over a prolonged period of time is a crucial aspect to ensure clinically relevant changes that can be maintained beyond the intervention period (Walugembe, Sibbald, Ber, & Kothari, 2019). A systematic review on health interventions indicated that most of the studies employed the use of theories in their research as one of the key tools to impact positively on participants and for its sustainability (Iwelunmor et al., 2016).

This study used the IMB module. The IMB is a very powerful behavioral change tool that has been used over the years to improve and to sustain healthy lifestyle behaviors (Chang et al., 2014). The model had the potential in reducing CVD risk factors because is it composed of critical elements that were needed to adopt and maintain healthy behaviors. The constructs are based on social and health psychology theories that was developed to address limitations in social and health psychology theories such as the description of relationship among constructs, predictive validity of constructs, and the inclusion of constructs that are needed for understanding, changing, and sustaining healthy behaviors.

Some school-based interventions programs trained students (Gómez-Pardo et al.,

2016; Kaya, Blake, & Chan, 2015) to act as change agents or as peer educators to ensure sustainability of the programs while some interventions have also had the involvement of teachers and school authorities (Sharma, Kim, & Nam, 2018; Tarp et al., 2018; Mukamana & Johri, 2016).

In this study, students were not only trained to reduce CVD risk factors and protect themselves from CVD in adulthood, but were trained to act as change agents to their peers, home, and community to ensure sustainability. The study on the other hand also had the involvement of teachers and headmasters who were fully involved in the study and were trained on the module to also ensure sustainability of the intervention. This is because projects with training components are more likely to be sustained than those without. Those trained would continue to provide benefits, train others and form a constituency in support of the program (Sonnino, 2016; Simane & Zaitchik, 2014).

Some studies have also reported that school-based interventions programs with support and cooperation from school administrators’, principals and teachers alike are good grounds for success and the sustainability of such intervention programs (Shahid& Bishop, 2019; Hout, Foley, McCormack, & Tardif, 2012). This is crucial for the sustainability of school-based intervention programs as this would ensure the smooth implementation of the health intervention before, during and after the intervention program to ensure sustainability of the program afterwards. Finally, having a more comprehensive structure in the health program and implementation process, from

training to materials may also be beneficial and lead to sustainment after the intervention.

**2.11 Conceptual Framework of the Study**

Figure 2.2 shows the conceptual framework of this study. The study therefore hypothesized that the behavioral modification intervention to reduce CVD risk factors using the IMB model would improve CVD knowledge, motivation, behavioral skills, physical activity, healthy eating, and decrease smoking, alcohol (primary outcomes), body weight, BMI, diastolic and systolic BP (secondary outcomes), and eventually would lead to positive and sustainable health behavior outcomes as explained by the IMB model to prevent the disease from occurring during adulthood. Other confounding factors (socio-demographic and family factors) affecting both primary and secondary outcomes were also acknowledged and adjusted for in the analysis.

**OTHER FACTORS AFFECTING PRIMARY AND SECONDARY OUTCOME**

 Socio-demographic factors

 Family demographic factors

**INTERVENTION**

BEHAVIORAL MODIFICATION INTERVENTION TO REDUCE CVD RISK FACTORS

USING THE INFORMATION MOTIVATION AND BEHAVIORAL SKILLS (IMB)

**PRIMARY OUTCOME**

 Knowledge

 Motivation

 Behavioral skills

**PRIMARY OUTCOME**


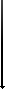

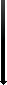

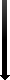


 Physical activity

**SECONDARY OUTCOMES**

 Weight

 BMI

**Blood pressure**

|  | Smoking |  | Diastolic  BP |
| --- | --- | --- | --- |
|  | Alcohol |  | Systolic  BP |
|  | Diet |  |  |

**Figure 2.2 : Conceptual Framework**

**CHAPTER 3**

**METHODOLOGY**

**3.1 Study Location**

Ghana is located on the West Coast of Africa and has a total land area of 238, 537 square kilometer. Ghana has a population of 24,658,823 (Ghana Statistical Service (GSS), 2012). The official language is English. Akan, Ga, Ewe and Dagbani are the most widely spoken local languages in the country. The country is divided into ten administrative regions namely Greater Accra, Ashanti, Brong Ahafo, Central, Eastern, Northern, Upper East, Upper West, Volta, and Western in Figure 3.1.

Located in mid-Ghana, Brong Ahafo (BA) region has 16.6% and 9.4% (males: 49.6% and female: 50.4%) of the total land area and population of Ghana respectively. The region has an average growth rate of 2.2% and a tropical climate with temperatures averaging 24oC and a double pattern rainfall. The region has two main vegetation which are the moist semi-deciduous forest and the savanna woodland. The region serves as a transit point for travelers from the south to northern part of the country. Agriculture is the main economic activity in the region. Adult literacy rate in the region is 44.6% (55.4% illiterate) while the youth is 61.5% and the predominant ethnic group in the region is Akan out of over ten ethnic groups. There are about 57 secondary schools in the region which are spread across 29 districts. Two (2) districts were selected for this study and each of the districts had two public secondary schools and all were included in this study. These were Kintampo North Municipality and Nkoranza North District. The Kintampo North has 4.1% of the total population of the region. Males constitute 49.6% as compared to females who are 50.4%. The population in the municipality is mainly youthful as 42.4% are under 15 years and 60 years and above are only 6.1% (GSS, 2013). The Nkoranza North district on the other hand has 2.9% of the region’s population. The males (50.5%) in the district are slightly higher than their female counterparts (49.5%). Likewise in Kintampo North, a higher percentage (41.3%) of the population are under 15 years whereas a smaller number,

7.1% are 60 years and above. The age range of secondary school students in Ghana is from 14 to 19 years olD.

**3.2 Study Design**

This study is a two arm single blind parallel cluster randomized controlled trial involving four public secondary school students that will be recruited into the study from two selected districts in Brong Ahafo region and will be guided by the CONSORT statement (Moher et al., 2010). Schools will be the unit of randomization, intervention, and analysis. Respondents in the intervention arm will receive CVD behavioral modification intervention based on the IMB model, whereas the control group will not be exposed to the intervention but will go on with their normal school curricula. Students in the control group will be wait listed and will receive the intervention program at the end of the study. Baseline measurements of CVD knowledge and its risk factors, motivation, behavioural skills, physical activity, smoking, alcohol consumption, dietary intake, body weight, height, diastolic and systolic BP will be taken for both the intervention and control groups. A post intervention assessment will then be carried out within two weeks immediately after six months to evaluate the effectiveness of the intervention.

**3.3 Study Duration**

The study will be conducted from 7th September, 2015 to 31st January, 2019. This will be from the development of the behavioral modification intervention module to reduce cardiovascular disease risk factors among public secondary school students in Kintampo North and Nkoranza North districts, Brong Ahafo region, implementation of module, evaluation, and submission of thesis.

**3.4 Sampling**

**3.4.1 Study Population**

The study population will be all the public secondary school students who will be enrolled in the 2018/2019 academic year in Kintampo-North and Nkoranza-North districts, Brong Ahafo region.

**3.4.2 Sampling Population**

Students attending secondary school will be recruited into the study. Students who will be in the public secondary schools in the districts will constitute the sampling frame. Students will be selected into the study based on the inclusion and exclusion criteria.

**3.4.2.1 Inclusion Criteria**

The inclusion criteria into this study will be all public secondary school students in Kintampo North and Nkoranza North districts, Brong Ahafo region.

**3.4.2.2 Exclusion Criteria**

The exclusion from this study will be students who are physically challenged such as students on wheelchair and could not accurately measure body weight and height or suffering from serious medical conditions such as heart diseases, asthma, or respiratory conditions or advised by a medical professional to restrict physical activities/exercise.

**3.5 Sampling Technique**

**3.5.1 Sampling Frame**

The sampling frame will be the list of all public secondary schools in Kintampo North and Nkoranza North districts, Brong Ahafo region.

**3.5.2 Sampling Method**

Duration of time of recruitment to first follow-up visit in the study will be one month (four weeks), therefore participants will be recruited within a period of four weeks using the sampling frame (list of all secondary schools within the selected area). Systematic random sampling technique will be used to select students into the study from the list of sampling frame given by headmasters of the schools. Using table of random numbers an initial list of 950 students will be selected across the four schools for possible inclusion into the study. Selection will be done separately for each school; each school will be taken as a cluster. The list of selected students will therefore be compiled and will be given serial numbers based on schools. Random allocation of schools (clusters) to groups will be done after baseline data collections.

**3.6 Sample Size Estimation**

The minimum sample size required to test the study hypotheses was calculated using the two proportions formula (Lemeshow, Hosmer, Klar, & Lwanga, 1990). The formula gave the required number (n) for each group.

2

{√2p̌ (1 − p̌) + Z1−β√p1(1 − p1) + p2(1 − p2)}

n =

(p1 − p2)2

Parameters from different intervention studies were substituted into the formula to calculate sample size as shown in Appendix H. However, a study among students by Mohammed et al. (2016) was adopted to calculate the sample size for this study.

P1 = 0.032 (proportion of students who initiated smoking among students in the intervention group after six months intervention.

P2 = 0.088 (proportion of students who initiated smoking among students in the control group after six months.

P = proportion of all subjects receiving smoking intervention after six months ((P1 +

P2)/2).

P = (0.032 + 0.088)/2 = 0.06

With Z1-α/2 = 1.96 at 95% CI and Z1-β = 1.28, at 90% power gave a minimum sample

size (n0) of 281

Adjustments were made for estimated response rates (Aday & Cornelius, 2006) thus:

i) Adjustment for estimated response rate, where the estimated attrition rate

is 20% = 337.2

This gave a sample size of 337.2 students in each group. Cluster randomized control trials are measured by design effect given as (1+ (M-1) X ICC) = 1.085.

ICC – Intra Cluster Correlation = 0.01

M – Average Cluster Size

337.2 x 1.085 (effect size of cluster random sampling) = 365.9

This gave a sample size of 366 students per group, which was then multiplied by two since the study is in two arms. Therefore, 732 is the sample required for this study.

**3.7 Randomization**

Randomization technique in the ratio 1:1 for intervention and control groups respectively will be carried out. Public secondary schools (clusters) will be the unit of randomization. Schools (students) will be randomly assigned to either the intervention or control group of the study on the same day participants will be selected for this study. It will however be performed after baseline data has been collected.

**3.7.1 Sequence Generation**

A biostatistician who will not be involved in the trial will generate the allocation sequence. Using a block randomization (1:1 ratio) of two digit blocks A and B (each containing one intervention and one control, to ensure equal distributions in the two groups will be used), two schools each will be allocated to intervention and control groups.

**3.7.2 Allocation Concealment Mechanism**

Allocation concealment will be achieved with sequentially numbered opaque sealed envelopes by an education officer containing treatment allocation cards (intervention and control). The envelopes will then be serially numbered from the outside.

**3.7.3 I**m**plementation**

An education officer who knows nothing about the research and not affiliated to any of the schools will be responsible for opening the envelopes in sequence and assigned schools to the groups. A second education officer will then document the group to which each school belong on a piece of paper and announce again to the school representatives.

**3.8 Blinding**

This study is single-blinded. Health staff who will take measurements of students’ anthropometry and blood pressure measurements will be blinded to participants groups and will not take anthropometry and blood pressure measurements at the post-test intervention. Researchers and physical education health instructor who that will facilitate the health education and the physical activity modules respectively will not be blinded as such. Contamination of the study will be minimized since there will be intervention and control schools and that schools are far apart each other.

**3.9 Development of the School CVD Risk Factor Reduction Intervention**

**Module for Secondary School Students**

Figure 3.2 shows a schematic diagram for the development of the school centered CVD risk factor reduction among public secondary school students module. The module will be developed through the process of consultations with group of experts. These will include experts from Universiti Putra Malaysia, the Ghana Health Service and expert teachers from Kintampo North and Nkoranza North districts. Their expertise will include; the area of public health, behavioral intervention, non-communicable diseases, and health education and promotion.

The training module will be developed for students which will serve as a guide for CVD risk factor reduction. CVD risk factor reduction module will be developed based on the Information-Motivation-Behavior Skills (IMB) model by Fisher and Fisher (1992) and divided into three sections. Table 3.1 shows how the IMB concepts will be addressed by the module. Session one is the general information phase. This includes the general overview of the knowledge of CVDs, its risk factors, types of CVD risk factors; modifiable (physical inactivity, smoking, alcohol, and unhealthy diet) and non- modifiable (such as age, gender, race, and ethnic background), how intermediary risk factors cause CVDs, how CVDs develop, and its prevention in terms of modifiable risk factors in the intervention schools. This will be carried out in the form of talks, workshops, and discussion sessions.

The second session will be the motivational phase. Students will be encouraged to perform the required physical activity on a daily basis, eat healthy foods, stop/not initiate smoking and alcohol and that healthy lifestyles would lead to being smart, healthy and free from obesity, diabetes, and being hypertensive. Students will be motivated that they would also be free from other diseases. They will also be encouraged to educate their peers. Poster, sport and dancing competitions, group work and interactions and role plays will also be carried out to motivate students.

Session three will be the behavioral skills phase. In this session students will be taught to demonstrate the ability to select their foods, to reduce sugar, and salt intake, to choose the appropriate fruits and vegetables, and the skills to perform the various forms of physical activities such as aerobic and anaerobic exercises, stop/not initiate smoking and alcohol consumption.

**Step 1:**

Consultation from experts in the area of public health, behavioral intervention, non-

communicable diseases, and health education and promotion

**Step 2:**

Development of the CVD risk factor reduction module among public secondary

school students based on the IMB Model

**Step 3:**

Presentation of the CVD risk factor reduction among public secondary school

student module draft to the experts

**Step 4:**

Improvement and modification to the CVD risk factor reduction among public

secondary school students module made

**Step 5:**

Final CVD risk factor reduction module among public secondary school students

produced

**Figure 3.2 : Steps Involve in the Development of the Intervention Module**

**Table 3.1 : Application of the Information-Motivation-Behavioral Skills Model for school based CVD risk factor reduction intervention module**

**IMB MODEL SECONDARY SCHOOL CVD RISK FACTOR**

**REDUCTION MODULE INFORMATION** Knowledge of cardiovascular disease and its risk

factors:

-Students will be informed how modifiable risk factors translates into intermediary risk factors and into CVDs

Prevention of CVDs through modifiable risk factors: Students will be taught on reduction of modifiable risk

factors of CVDs. These will include:

Physical Activity: Doing at least 60 minutes moderate to vigorous physical activity daily.

Diet: Consumption of healthy diet devoid of too much

salt, too much sugar, too much fat, too much fried foods, and red meat. Consumption of fruits and vegetables, drinking enough plain water instead of coloured (sugar) water, fish, whole grains instead of polished grains.

Smoking: Information on tobacco, stopping smoking (for student smokers) and not initiating smoking for non-smokers

Alcohol: Same with alcohol consumption as with smoking.

**MOTIVATION** Students will be encouraged that if they perform the required physical activity on daily basis and eat healthy foods they would become smart and healthy and would be free from overweight, obesity, diabetes, and being hypertensive. They would also be free from other diseases.

They will further be encouraged and taught that not initiating or stopping smoking and alcohol would make their lungs and heart function properly, strong, and free from heart diseases.

Peer groups to educate fellow students to serve as a source of motivation.

Poster, sport and dancing competitions, group work

and interactions, and role plays to motivate students.

**BEHAVIOURAL SKILLS** Students will be taught to demonstrate the ability to select their foods, to choose drinks with reduced sugar content, to reduce salt intake, to choose the appropriate fruits and vegetables, to know the skill to perform the various forms of physical activities (such as jumping jacks, hamstring, push-ups, squat, sit ups, and other aerobic exercises), stop/not initiate smoking and alcohol consumption.

**3.9.1 Validation of the Module**

The module will be reviewed by a panel of experts in public health, behavioral intervention, non-communicable diseases, and health education and promotion. The experts will give their consensus that the sections of the module will be valid in content, teaching methods and learning objectives.

**3.10 Training**

There will be two clinicians who will be involve in this study with specialty in public health. Also included will be twelve qualified health staff with Master’s degree qualification, and a physical education health instructor (with Diploma in Physical Education) who will implement the physical activity module of the intervention. All these staff are with the Kintampo Health Research Centre (Ghana Health Service). Clinicians, health staff, and PE health instructor will be selected if they had been involved previously in any of the Ghana Health Service field research. Health staff will be trained by the researcher and clinicians to take anthropometry (body weight and height) and blood pressure (diastolic and systolic BP) measurements. Health staffs (six) who will be involve in the baseline measurements will not take part in the post intervention measurements. Another set of six health staff will do the post intervention measurements to avoid observers’ bias. The objectives of the study will be explained to staff involved in the study. A week’s training will be organized for health staff on anthropometric and blood pressure measurements. The training of health staff will among other things to equip them to avoid inter and intra observer measurements biases.

The study has the involvement of school authorities. A week’s training will be organized for PE and other teachers in the intervention schools by the research team (researcher, clinicians, PE health instructor, and health staff). This will be done to ensure sustainability after the intervention to train new students. The study protocol including all measurements will be reviewed with the clinicians, health staff, physical education health instructor, and teachers before the commencement of the study.

**3.11 Intervention**

At this stage permissions and consent would had been sought from the Ministry of Education, District Directors of Education, Chairmen of the Parent Teacher Associations, parents/guardians, and students to carry out the study. All materials for the study will be ready at this stage. The objectives of the study and the eligibility criteria to participate in this study will be explained to all the students’ involved. Students will go through medical check-up which will be carried out by the two clinicians in order to pass eligibility criteria to be enrolled into the study.

The self-administered questionnaire will be given to students to complete. Health staff will measure students anthropometric and blood pressure measurements. Confidentiality and anonymity will be assured at all stages of the study. The module will be implemented in the intervention schools for a period of six months but not in the control schools. Then at the end of six months the same questionnaire will be given to students in both the intervention and the control schools. Health staff who will not take part during the baseline data collection will take anthropometric and blood pressure measurements of students at post intervention study. A total of 848 students will participate in the study; intervention (424) and the control (424) schools. Contamination of the intervention will be prevented by having intervention and control schools with distances between them.

**3.12 Data Collection**

Data will be collected using validated and pre-tested questionnaire. Data will be collected at baseline and at six months post intervention where socio-demographic data, physical activity score, smoking, alcohol consumption, dietary intake, height, body weight, diastolic and systolic BP, CVD knowledge, motivation and behavioral skills scores will be collected.

**3.12.1 Study Instruments**

A self-administered questionnaire, which will be validated and pre-tested will be used to collect data. Weight and height scales will be used to collect data on body weight and height measurements of students respectively. Then a diastolic and systolic apparatus will be used to measure blood pressure of participants. Body weight, height, diastolic, and systolic blood pressure measurements will be taken by the health staff.

**3.12.2 Questionnaire**

The questionnaire will be pre-tested and assisted self-administered questionnaire in English language as in Appendix. The questionnaire will be divided into ten sections namely, socio demography, dietary intake, smoking, alcohol consumption, physical activity, CVD knowledge, motivation, behavioral skills, BMI (weight and height), and blood pressure (diastolic and systolic). The questionnaires will be administered in a classroom setting in all the selected schools.

Section one of the questionnaire will be used to access students’ age, gender, grade, ethnicity, monthly pocket money, and family history. Data on age will be collected in the form of open ended question and students will fill in the blank space provided. The rest (ethnicity, gender, religion, and family history) are in the form of closed ended questions and students are to choose the answers that will pertained to them.

Section two is a seven-day dietary recall instrument which are foods items mostly consumed in Ghana, the Ghana Demographic and Health Survey, (GDHS), (GSS, 2009; Nti, 2008) to assess dietary consumption. Amounts of each food intake will be stated with the use of handy measure tables (food weights). This are based on the weights of foods with their equivalent handy measure tables which are commonly used

in Ghana (Owusu, Ankrah, Boateng, & Anteh, 1995). Then frequency of consumption of certain foods will be scored based on one (1) for never dietary consumption, two (2) for 2-3 times per week, three (3) for 4-5 times per week, four (4) for 6 times per week, and five (5) for daily intake of a particular food item.

Section three and four are the modified smoking and alcohol questionnaires, Global Youth Tobacco Survey (GYTS) developed by WHO and Centre for Disease Control and Prevention (CDC) (CDC, 2008). It is a school-based adolescents’ self- administered questionnaire which comprised of a set of core questions that will be used to evaluate tobacco and alcohol control and prevention programmes among secondary school students. Students would be classified as never, ever, and current smokers and alcohol consumption. Strict confidentiality will be ensured among researchers to avoid disclosure of smoking and alcohol statuses to school authorities, teachers, or parents.

Section five consists of physical activity questionnaire (PAQ-A) for secondary school students (Kowalski, Crocker, & Donen, 2004). This is a 7-day recall instrument which will be administered in a classroom setting to assess general levels of physical activity among secondary school children. Students will be informed before this section is administered that is not a test but a tool for assessing actual physical activity during the past seven (7) days. Each physical activity item will be scored on a five (5) point scale. The value from one (1) to five (5) for each of the items used in the physical activity composite score, then the mean of the items which resulted in the final PAQ-A activity summary score.

Section six will be the modified CVD knowledge assessment questionnaire which comprise of meaning of CVDs, risk factors and preventive strategies of CVDs to assess CVD knowledge score among respondents. The school-based instrument was developed by George et al. (2013) to assess CVD knowledge levels among students. The scoring of the instrument is in the form of true or false questions and unsure (don’t know) response for each question. Respondents are to choose either true, false or ‘’don’t know’’ by circling in the provided box. Each correct answer will be awarded one score. The maximum score is 26 and the minimum score is 0.

Section seven and eight are the Motivation and Behavior skills respectively, which are the modified versions of the LifeWindows Information-Motivation-Behavioral skills questionnaires (The LifeWindows Team, 2006) which constructs were originally developed by (Fisher, Fisher, Amico, & Herman, 2006). Motivation constructs assess questions on personal and social motivation relating to how motivated or encouraged participants are to perform a CVD risk factor reduction lifestyle. Scoring of responses are based on the likert scale from ‘I strongly disagree to I strongly agree’. Each of the items are scored based on the responses from 0 to 3;

with strongly disagree as 0 to strongly agree as 3. All reverse items will be scored asuch before the final scores are determined.

Behavioral skills questionnaire will test students’ ability to reduce CVD risk factors. The questions will assess their behavior skills including how hard or how easy is to apply their behavioral skills. The responses are ‘very hard to very easy’ with a scale from 0 to 3. Very hard is awarded a score of 0 while very easy has a score of 3. Then the mean scores will be determined.

**3.12.3 Anthropometry Measurements**

Section nine is the anthropometric measurements that consist of weight and height. Height will be measured using a SECA Body Meter Model 208 (Rampal, Rampal, Azhar, & Rahman, 2008; Rampal et al., 2009) to the nearest 0.1cm. The respondent will be asked to stand and look straight, barefooted with heels resting together. Height measurement which appears in the read-off area will be recorded. Weight of student will be measured using digital bathroom scale, TANITA Model HD 309 (Rampal et al., 2008; Rampal et al., 2009) to the nearest 0.1kg. The student will be requested to remove slippers or shoe and anything in the pocket. He/she will then be asked to stand upright on the scale and the measurement will be taken. Height and weight measurements will be used to assess students’ body mass index (BMI) as a proxy to their nutritional status in terms of underweight, normal, overweight, and obesity. Height and weight will be measured by qualified health staff. Measuring instruments will be standardized on a daily basis before they are used.

**3.12.4 Blood Pressure Measurements**

Section ten is the blood pressure measurements. Two blood pressure readings will be taken with an Omron HBP-1100 automated BP monitor. This will be measured by qualified health staff with the student comfortably seated for five minutes with the legs not crossed and the back and the arm supported before measurements will be taken. A minute’s rest interval will be allowed after the first reading before the second measurement will be taken (Pickering et al., 2005). The average of the two will be used to classify students.

**3.13 Quality Control of Study Instruments**

**3.13.1 Validity of Questionnaire**

Generally, the validity of an instrument refers to how accurately the instrument measures what it is actually intended to measure. The study instruments in this research will be face validity and content validity.

**3.13.2 Face Validity**

Face validity establishes whether the measure under study seems to be assessing the intended construct under study. The questionnaire will be face validated among a convenient sample of 50 students. They will be asked to assess each of the questionnaire in order of questions (good, average or poor), clarity (clear, average or confusing) and its appropriateness (good, average or poor).

**3.13.3 Content Validity**

A content validity will be executed to determine the validity of the questionnaire. Content validity involves verifying all questions in order to systematically examine the test content to know if it is representative of the sample to be measured. In other words, it is the content of the instrument measuring the intended variables of the research. The content validity will be determined by the supervisory committee from the Community Health Department, Universiti Putra, Malaysia, who will check on the content of the questionnaire. They will then make suggestions to improve these measures. They will check the extent to which the items will be representative concept being measured. The content validity of questionnaire will then be evaluated by specialist in the field of epidemiology, cardiology, and community health.

**3.13.4 Validity of the Anthropometric Measurement**

Anthropometric measurements in this study are weight, height, and blood pressure. All students in the study will be measured equally with the same weight, height, and blood pressure instruments. The study protocol procedures (including measurements of weight, height and blood pressure) will be reviewed with the study team through training sessions before the study will commence. The study instruments will be calibrated daily before use.

**3.13.5 Reliability of Questionnaire**

Reliability refers to the extent to which a questionnaire, test or observation or any measurement procedures yields the same result on repeated trials. It can also be said to be the strength, consistency or reproducibility of scores over time. Training sessions by the researcher and the two clinicians will be conducted for qualified health staff who will take anthropometry and blood pressure measurements to reduce inter and intra observer variability in the measurements in order to increase the reliability of instruments and develop the skill of measurements in order to collect accurate data.

**3.14 Implementation of Intervention**

The CVD risk factor reduction intervention module is basically divided into two sections and will be guided by the IMB model. These are the Health Education and the Physical Activity Modules. The interactive health education sections, the researcher will do education in the schools on CVDs, its risk factors, causes, development and prevention among others in the intervention schools. The intervention schools will be visited three times in a week for a period of six months. Each section of the health education will last for about an hour with a break, and would be followed by questions, answers and discussions.

The physical activity module which consist of aerobics and anaerobic exercises will be delivered by a physical education health instructor in Kintampo health research center (Ghana health service). The instructor will teach students first on the various physical activities and then take them to the school field to undertake the activities. Same exercises will be carried out in the intervention schools. Each physical activity session that will be given to respondents in the intervention group will last for about 25-30 minutes. A summary of the intervention is shown in Table 3.4.

**Table 3.4 : Summary of intervention module**

**MODULE COMPONENTS FORM OF**

**ESTIMATED**

**DELIVERY TIME**

ur per

| **Health**  **Education**  **Module** |  | Module introduction |  Lectures   Discussions | One ho  session |
| --- | --- | --- | --- | --- |
|  |  | CVD introduction |  | |
|  |  | CVD risk factor introduction |  |  |
|  |  | Harmful effects of smoking quitting smoking |  |  |
|  |  | Barriers to quitting smoking |  |  |
|  |  | Health benefits of quitting/not initiating smoking |  |  |
|  |  | Physical activity introduction |  |  |
|  |  | Simple physical activities/exercises |  |  |
|  |  | Fruits and vegetables introduction |  |  |
|  |  | Fruits and vegetables intake/selection |  |  |
|  |  | Introduction to the types of fats |  |  |
|  |  | Outcomes of high fat intake on  health |  |  |
|  |  | Harmful effects of obesity |  |  |
|  |  | Harmful effects of excessive sugar intake |  |  |
|  |  | Promotion of frequent water  intake |  |  |
|  |  | Harmful effects of excessive salt intake |  |  |
|  |  | Harmful effects of alcohol |  |  |
|  |  | Quitting alcohol |  |  |
|  |  | Barriers to quitting alcohol |  |  |
|  |  | Health benefits of quitting/not initiating alcohol |  |  |

**Physical**

**Activity Module**

 Aerobics

Anaerobic:

 Lunge with twist

 Jumping jacks

 Abdominal crunch

 Hamstring stretch

 Wall sit

 Side arm and leg raise

 Push ups

 Sit and reach

 Sit ups

 Knee to chest

 Leg raise

 Squat

Hands on (Field exercise)

25-30 minutes per session

**3.15 Variables**

**3.15.1 Independent Variable**

The independent variable of this study is the behavioral modification intervention program to reduce cardiovascular disease risk factors based on the IMB model among secondary school students in two districts of Brong Ahafo, Ghana.

**3.15.2 Dependent Variables**

The dependent variables of this study are sub divided into primary and secondary outcomes. There were;

i. The primary outcomes are CVD knowledge and its risk factors, motivation, behavioral skills, physical activity, smoking, alcohol consumption, and dietary intake.

ii. The secondary outcomes are body weight, BMI, diastolic and systolic blood pressures.

**3.15.3 Confounders**

The confounders in this study are age, gender, ethnicity, monthly pocket money,

family history of obesity, parent’s education and their occupation.

**3.16 Operational Definition of Terms**

**3.16.1 Body Mass Index**

Weight in kilograms (kg) and height in meters (cm) were measured to determine BMI calculated as weight in kilograms divided by the square of height in meters (kg/m2). Subjects will be classified based on mean BMI for age and gender for children and adolescents. Adolescents were categorized into four groups which were underweight, normal weight, overweight, and obese with cut off points of <5th, 5th to < 85th, 85th to < 95th and ≥95th percentiles of the WHO standard respectively (Barlow, 2007; WHO, 2007).

**3.16.2 Hypertension**

Hypertension, also referred to as high or raised blood pressure is defined as a condition in which the blood vessels have persistently raised pressure thereby putting the blood vessels under stress. Diastolic and systolic BP will be measured in millimeter mercury (mmHg). According to the fourth report on the diagnosis, evaluation, and treatment of

high blood pressure in children and adolescents (National Institute of Health, 2005), blood pressure (DBP or SBP) is classified into normal (≥ 60th and < 90th) pre- hypertension (≥ 90th and < 95th), hypertension stage 1 (≥ 95th and < 99th) plus 5 mmHg, and hypertension stage 2 (≥ 99th) plus 5 mmHg.

**3.16.3 Physical Activity**

Physical activity refers to the total number of questions in the PA that were answered by participants.

**3.16.4 Smoking**

Smoking will be categorized into ex-smokers, current smokers, and never smoker. Ever smokers were students who had ever tried smoking before but had stopped. Current smokers are those who are currently smoking tobacco. Never-smokers referred to as students who has never smoked tobacco.

**3.16.5 Alcohol**

This refers to the consumption of alcoholic beverages. These include but not limited to drinks such as beers, wine, ‘’pito’’ (made from fermented cereal such as millet), spirits, and ‘’akpeteshie’’ (made from distilling sugar cane).

**3.16.6 Diet Practices**

This referred to the total frequency in scores and amount of diet for each food item. Consumption of foods such as fruits, vegetables, seafood, and water were considered as healthy foods while the consumption of foods such as fried foods, fried eggs, fried chicken, fats and oils, plain sugar, sweet snacks, salted fish, and carbonated drinks are considered as unhealthy foods.

**3.16.7 CVD Risk Factor Knowledge**

CVD knowledge are the risk factors and preventive strategies of CVDs. This refers to the total sum of knowledge scores for all items of the CVD knowledge.

**3.16.8 Motivation**

Motivation refers to the total number of questions in the motivation questions that are answered correctly by participants.

**3.16.9 Behavioral Skills**

This refers to the total sum of behavioral skills scores for all items in this section of the questionnaire.

**3.17 Data Analysis**

Data collected will be collated, checked, cleaned, and analyzed using IBM Statistical Package for Social Sciences (SPSS) version 22. The data analysis will be divided into descriptive and inferential statistics. The level of significance for all the statistical tests is set at 0.05. For mean estimations, 95% confidence intervals (CI) will be computed.

**3.17.1 Normality Tests**

Normality tests will be performed for age, CVD knowledge, motivation, behavioral skills, physical activity, body weight, height, BMI, diastolic, and systolic BP. Because sample size will be more than 300, histogram and total values of not more than 2 in the case of skewness and 7 on kurtosis (Kim, 2013) and also not more than a total value of 1 (Tabachnick & Fidell, 2007), will be used to determine the requirements of this assumption.

**3.17.2 Descriptive Statistics**

For descriptive statistics, means and standard deviations will be used as measure of central and dispersion to summarize continuous variables. Frequencies and percentages will be used to summarize categorical variables.

**3.17.3 Inferential Statistics**

Chi-square tests will be performed to compare baseline characteristics of groups by their socio-demographic factors, family factors, CVD knowledge, motivation, behavioral skills, physical activity, smoking status, alcohol intake, BMI, systolic and diastolic BP. Independent t-tests will be performed to determine the between groups differences of total knowledge, motivation, behavioral skills, physical activities, dietary intake, BMI, diastolic and systolic BP. At six months post intervention, the independent and paired sample t-tests would be performed to determine the between and within group differences of variables respectively. Chi-square and the McNemar tests will also be performed on smoking status and alcohol intake at six months to determine the difference between and within groups since the variables are categorical variables.

A mixed design repeated measures analysis of variance (ANOVA) will be performed after checking that there is no substantial non-normality of residuals to determine the between and within group differences and its interactions on time differences on CVD knowledge, motivation, behavioral skills, physical activity, dietary intake, body weight, BMI, diastolic and systolic BP. Intention-to- treat (ITT) analysis will be the method that will be used to handle missing data in the analysis. Replacement of data will then be conducted for each missing variable using the multiple imputation method, following which Generalized Linear Mixed Model (GLMM) analysis will be performed to determine the overall effectiveness of the intervention study.

**3.17.4 Effect Size**

The two measures of effect size that will be used in this study are the partial eta squared and the coefficient of fixed effect. Partial eta squared, which is the proportion of the total variance in a dependent variable which is due to the intervention, will be utilized in the mixed design repeated measures ANOVA analysis. Its classification is based on small (0.01), medium (0.06) and large (0.14) (Cohen, 1988). The magnitude, also referred to as coefficient of fixed effects will be used in the GLMM analysis.

**3.17.5 Sensitivity Analysis**

Sensitivity analysis will be performed to determine the effect of drop outs in the study. The GLMM analysis will be performed and this time without replacing the missing values. The percentage differences between the coefficients will be obtained after replacement of missing data will then be calculated.

**3.18 Ethical Consideration**

Ethical approval will be sought from the UPM Ethics Committee for Human Research before the study will be conducted. Ethical clearance will also be obtained from the Kintampo Health Research Centre Institutional Ethics Committee. Permissions will be sought from the Ministry of Education, headmasters/headmistresses, and Chairpersons of Parent Teacher Association (PTA) of selected schools. The study will be registered with the Pan African Clinical Trials Registry. A written and signed informed consent from all participating students and their parents or guardians will be obtained and forms will be kept under lock and key at the office. Students will be informed that they could withdraw from participating in the study at any time. To ensure confidentiality, a code number will be given to each student in both the intervention and control groups and they would be asked to use the codes on their completed questionnaires.

**REFERENCES**

Abdulle, A., Al-junaibi, A., & Nagelkerke, N. (2014). High blood pressure and its association with body weight among children and adolescents in the United Arab Emirates. *PloS ONE*, *9*(1), 1–5. doi:10.1371/journal.pone.0085129.

Abraham, S., Noriega, B., & Shin, J. (2018). College students eating habits and knowledge of nutritional requirements. *Journal of Nutrition and Human Health*,

*2*(1), 12–17. doi:[10.35841/nutrition-human-health.2.1.13-17.](https://doi.org/10.35841/nutrition-human-health.2.1.13-17)

Adab, P., Pallan, M. J., Lancashire, E. R., Hemming, K., Frew, E., Griffin, T., & Cheng, K. K. (2015). A cluster-randomised controlled trial to assess the effectiveness and cost-effectiveness of a childhood obesity prevention programme delivered through schools, targeting 6-7 year old children: the WAVES study protocol. *BMC Public Health*, *15*(1), 1-10. doi:10.1186/s12889-

015-1800-8.

Aday, L. A., & Cornelius, L. J. (2006). Designing and conducting health surveys: a comprehensive guide, edition (3rd ed.). San Francisco, CA: John Wiley & Sons Ltd. Page 20-51.

Agyei-Mensah, S., & de-Graft Aikins, A. (2010). Epidemiological transition and the double burden of disease in Accra, Ghana. *Journal of Urban Health*, *87*(5), 879–

897. doi:10.1007/s11524-010-9492-y.

Agyemang, C., Snijder, M., Adjei, D., Van den Born, B., Modesti, P., Peters, R., & Vogt, L. (2016). Ethnic disparities in CKD in the Netherlands: The Healthy Life in an Urban Setting (HELIUS) Study. *American Journal of Kidney Disease*,

*67*(3), 391–399. doi:10.1053/j.ajkd.2015.07.023.

Alaimo, K., Carlson, J. J., Pfeiffer, K. A., Eisenmann, J. C., Paek, H., Betz, H. H., & Norman, G. J. (2015). Project FIT: a school, community and social marketing intervention improves healthy eating among low-income elementary school children. *Journal of Community Health*, *40*(4), 815–826. doi:10.1007/s10900-

015-0005-5.

Alangea, D. O., Aryeetey, R. N., Gray, H. L., Laar, A. K., & Adanu, R. M. K. (2018).

Dietary patterns and associated risk factors among school age children in urban

Ghana. *BMC Nutrition*, *4*(22), 1–9. doi:10.1186/s40795-018-0230-2.

Albarracin, D., Johnson, B., Fishbein, M., & Muellerleile, P. (2001). Theories of reasoned action and planned behavior as models of condom use: a meta-analysis. *Psychological Bulletin*, *127*(1), 142–161. doi:10.1037/0033-2909.127.1.142.

Archer, E., & Blair, S. N. (2011). Physical activity and the prevention of cardiovascular disease: from evolution to epidemiology. *Progress in Cardiovascular Diseases*, *53*(6), 387–396. doi: 10.1016/j.pcad.2011.02.006.

Aryeetey, R., Lartey, A., Marquis, G., Nti, H., Colecraft, E., & Brown, P. (2017).

Prevalence and predictors of overweight and obesity among school-aged children in urban Ghana. *BMC Obesity*, *4*(1), 1–8. doi:10.1186/s40608-017-0174-0

August, K. J., & Sorkin, D. H. (2011). Racial/ethnic disparities in exercise and dietary behaviors of middle-aged and older adults. *Journal of General Internal Medicine*,

*26*(3), 245–250. doi:10.1007/s11606-010-1514-7.

Awuah, R. B., Anarfi, J. K., Agyemang, C., Ogedegbe, G., & Aikins, A. de-G. (2014).

Prevalence, awareness, treatment and control of hypertension in urban poor communities in Accra, Ghana. *Journal of Hypertension*, *32*(6), 1203–1210. doi:

10.1097/HJH.00000000000000165.

Baer, D., Jones, R. M., Mcclish, D., Westerberg, A. L., & Danish, S. (2012). Fruit and vegetable intake among rural youth following a school-based randomized controlled trial. *Journal of Preventive Medicine*, *54*(2), 150–156. doi:10.1016/j.ypmed.2011.11.005.

Balagopal, P., de Ferranti, S. D., Cook, S., Daniels, S. R., Gidding, S. S., Hayman, L.

L., & Steinberger, J. (2011). Nontraditional risk factors and biomarkers for cardiovascular disease: mechanistic, research, and clinical considerations for youth: a scientific statement from the American Heart Association. *Journal of Circulation*, *123*(23), 2749–2769. doi:10.1161/CIR.0b013e31821c7c64.

Barlow, S. (2007). Expert committee recommendations regarding the prevention, assessment, and treatment of child and adolescent overweight and obesity: summary report. *Paediatrics*, *120*(Suppl 4), s164–s165. doi:10.1542/peds.2007-

2329C.

Barrett-Connor, E. (2013). Menopause, atherosclerosis, and coronary artery disease.

*Current Opinion in Pharmacology*, *13*(2), 186–191. doi:10.1016/j.coph.2013.01.005.

Bastien, M., Poirier, P., Lemieux, I., & Després, J. (2014). Overview of epidemiology and contribution of obesity to cardiovascular disease. *Progress in Cardiovascular Diseases*, *56*(4), 369–381. doi:10.1016/j.pcad.2013.10.016.

Bauer, U. E., Briss, P. A., Goodman, R. A., & Bowman, B. A. (2014). Prevention of chronic disease in the 21st century: Elimination of the leading preventable causes of premature death and disability in the USA. *The Lancet*, *384*(9937), 45–52. doi:10.1016/S0140-6736(14)60648-6.

Black, J. A., Park, M., Gregson, J., Falconer, C. L., & White, B. (2015). Child obesity cut-offs as derived from parental perceptions: *British Journal of General Practice*, *65*(633), 234–239. doi:10.3399/bjgp15X684385.

Boateng, D., Wekesah, F., Browne, J. L., Agyemang, C., Agyei-baffour, P., Aikins, A. de-G., & Klipstein-grobusch, K. (2017). Knowledge and awareness of and perception towards cardiovascular disease risk in sub-Saharan Africa: a systematic review. *PLoS ONE*, *12*(12), 1–21. doi:10.1371/ journal.pone.0189264.

Boateng, G. O., Luginaah, I. N., & Taabazuing, M. M. (2015). Examining the risk factors associated with hypertension among the elderly in Ghana. *Journal of Aging and Health*, *27*(7), 1-23. doi:10.1177/0898264315577588.

Boeing, H., Bechthold, A., Bub, A., Ellinger, S., Haller, D., Kroke, A., et al. (2012).

Critical review: vegetables and fruit in the prevention of chronic diseases.

*European Journal of Nutrition*, *51*, 637–663. doi:10.1007/s00394-012-0380-y.

Bosu, W. (2013). Accelerating the control and prevention of non-communicable diseases in Ghana: the key issues. *Postgraduate Medical Journal*, *2*(1), 32–40.

Bosu, W. K. (2010). Epidemic of hypertension in Ghana: a systematic review. *BMC Public Health*, *10*(1), 1-14. doi:10.1186/1471-2458-10-418.

Brady, C. (2016). Decreasing obesity and obesity stigma: socio-demographic differences in beliefs about causes of and responsibility for obesity. *Journal of Social Sciences*, *5*(12), 1–10. doi:10.3390/socsci5010012.

Brown, I. J., Dyer, A. R., Chan, Q., Cogswell, M. E., Ueshima, H., Stamler, J., & Elliott, P. (2013). Estimating 24-hour urinary sodium excretion from casual urinary sodium concentrations in western populations. the INTERSALT Study. *American Journal of Epidemiology*, *177*(11), 1180–1192. doi:10.1093/aje/kwt066.

Browne, S., Minozzi, S., Bellisario, C., Sweeney, M. R., & Susta, D. (2019).

Effectiveness of interventions aimed at improving dietary behaviours among people at higher risk of or with chronic non-communicable diseases: an overview of systematic reviews. *European Journal of Clinical Nutrition*, *73*(1), 7–23. doi:10.1038/s41430-018-0327-3.

Brustio, P. R., Moise, P., Marasso, D., Alossa, D., Miglio, F., Mulasso, A., & Boccia, G. (2018). Participation in a school-based walking intervention changes the motivation to undertake physical activity in middle-school students. *PLoS ONE*,

*13*(9), 1–13. doi:10.1371/journal.pone.0204098.

Buxton, C. N. A. (2014). Ghanaian junior high school adolescents dietary practices and food preferences: implications for public health concern. *Journal of Nutrition*

*& Food Sciences*, *4*(5), 1–9. doi:10.4172/2155-9600.1000297.

Carter, B. D., Abnet, C. C., Feskanich, D., Freedman, N. D., Hartge, P., Lewis, C. E.,

& Jacobs, E. J. (2015). Smoking and mortality-beyond established causes. *The New England Journal of Medicine*, *372*(7), 631–640. doi:10.1056/nejmsa1407211.

Cawley, J., Meyerhoefer, C., Biener, A., Hammer, M., & Wintfeld, N. (2015). Savings in medical expenditures associated with reductions in body mass index among us adults with obesity, by diabetes status. *Journal of PharmacoEconomics*, *33*, 707–

722. doi:10.1007/s40273-014-0230-2.

Centers for Disease Control and Prevention (CDC). Global youth tobacco surveillance. surveillance summaries, 2002-2007. (2008). (Vol.57). Atlanta. http//[:www.cdc.gov/mmwr](http://www.cdc.gov/mmwr) (accessed 21 Nov. 2015).

Champion, V. (1990). Breast self-examination in women 35 and older: a prospective study. *Journal of Behavioral Science*, *13*(6), 523–538. doi:10.1007/BF00844733.

Chang, S. J., Choi, S., Kim, S., & Song, M. (2014). Intervention strategies based on information-motivation-behavioral skills model for health behavior change: a systematic review. *Asian Journal of Nursing Research*, *8*(3), 172–181. doi:10.1016/j.anr.2014.08.002.

Chen, Y., Ma, L., Ma, Y., Wang, H., Luo, J., Zhang, X., & Wang, H. (2015). A national school-based health lifestyles interventions among Chinese children and adolescents against obesity: rationale, design and methodology of a randomized controlled trial in China. *BMC Public Health*, *15*(210), 1–10. doi:10.1186/s12889-015-1516-9.

Chiolero, A., Bovet, P., & Paradis, G. (2013). Screening for elevated blood pressure in children and adolescents. *JAMA Pediatrics*, *167*(3), 266–273. doi:10.1001/jamapediatrics.2013.438

Chomistek, A. K., Manson, J. E., Stefanick, M. L., Lu, B., Sands-Lincoln, M., Going, S. B., & Eaton, C. B. (2013). Relationship of sedentary behavior and physical activity to incident cardiovascular disease: results from the women’s health initiative. *Journal of the American College of Cardiology*, *61*(23), 2346–2354. doi:10.1016/j.jacc.2013.03.031.

Chrostowska, M., Szyndler, A., Hoffmann, M., & Narkiewicz, K. (2013). Impact of obesity on cardiovascular health. *Best Practice & Research Clinical Endocrinology & Metabolism*, *27*, 147–156. doi:10.1016/j.beem.2013.01.004.

Cohen, J. (1988). Statistical power analysis for thr behavioral sciences (2nd ed.). New

York: Academic Press, Inc. Pages 22-90.

Colley, R. C., Garriguet, D., Janssen, I., Craig, C. L., Clarke, J., & Tremblay, M. S. (2011). Physical activity of Canadian children and youth: accelerometer results from the 2007-2009 Canadian health measures survey. *Journal of Health Reports, 22*(1), 15-23.

Cunha, D. B., de Souza, B. da S. N., Pereira, R. A., & Sichieri, R. (2013). Effectiveness of a randomized school-based intervention involving families and teachers to prevent excessive weight gain among adolescents in Brazil. *PLoS ONE*, *8*(2), 1–

8. doi:10.1371/journal.pone.0057498.

DeBoer, M. D. (2013). Obesity, systemic inflammation, and increased risk for cardiovascular disease and diabetes among adolescents: a need for screening tools to target interventions. *Journal of Nutrition*, *29*(2), 379–386. doi:10.1016/j.nut.2012.07.003.

Demirci, H., Nuhoglu, C., Ursavas, I. S., Isildak, S., Basaran, E. O., & Kılıc, M. Y. (2013). Obesity and asymptomatic hypertension among children aged 6–13 years living in Bursa, Turkey. *Family Practice*, *30*, 629–633. doi:10.1093/fampra/cmt048.

Dhingra, R., & Vasan, R. (2012). Age as a cardiovascular risk factor. *Journal of Medical Clinics in North America*, *96*(1), 87–91. doi:10.1016/j.mcna.2011.11.003.Age.

Dewar, D. L., Morgan, P. J., Plotnikoff, R. C., Okely, A. D., Batterham, M., & Lubans, D. R. (2014). Exploring changes in physical activity, sedentary behaviors and hypothesized mediators in the NEAT girls group randomized controlled trial. *Journal of Science and Medicine in Sport*, *17*(1), 39–46. doi:10.1016/j.jsams.2013.02.003.

Doegah, P. T., & Amoateng, A. Y. (2018). Water intake among Ghanaian youth aged

15–34 years: quantitative and qualitative evidence. *Journal of Health, Population and Nutrition*, *37*(3), 1–10. doi:10.1186/s41043-018-0135-3.

Doku, D., Koivusilta, L., Raisamo, S., & Rimpelä, A. (2013). Socio-economic differences in adolescents’ breakfast eating, fruit and vegetable consumption and physical activity in Ghana. *Public Health Nutrition Journal*, *16*(5), 364–872. doi:10.1017/S136898001100276X.

Domitrovich, C. E., Bradshaw, C. P., Berg, J. K., Pas, E. T., Becker, K. D., Musci, R.,

& Ialongo, N. (2016). How do school-based prevention programs impact teachers? findings from a randomized trial of an integrated classroom management and social-emotional program. *Journal of Preventive Science*, *17*,

325–337. doi:10.1007/s11121-015-0618-z.

Drapeau, V., Savard, M., Gallant, A., Nadeau, L., & Gagnon, J. (2016). The effectiveness of a school-based nutrition intervention on children’s fruit, vegetables, and dairy product intake. *Journal of School Health*, *86*(5), 353–362. doi: 10.1111/josh.12385.

Dumith, S. C., Gigante, D. P., Domingues, M. R., & Kohl, H. W. (2011). Physical activity change during adolescence: a systematic review and a pooled analysis. *International Journal of Epidemiology*, *40*(3), 685–698. doi:10.1093/ije/dyq272.

Eather, N., Morgan, P. J., & Lubans, D. R. (2013). Improving the fitness and physical activity levels of primary school children: results of the Fit-4-Fun group randomized controlled trial. *Journal of Preventive Medicine*, *56*(1), 12–19. doi:10.1016/j.ypmed.2012.10.019.

Eather, N., Morgan, P. J., & Lubans, D. R. (2016). Improving health-related fitness in adolescents: the CrossFit Teens randomised controlled trial. *Journal of Sports Sciences*, *34*(3), 209–223. doi:10.1080/02640414.2015.1045925.

Eaton, D. K., Kann, L., Kinchen, S., Shanklin, S., & Ross, J. (2010). Youth risk behavior surveillance-United States, 2009 (Vol. 59). Atlanta.<http://www.cdc.gov/mmwr/pdf/ss/ss5905.pdf> (accessed 20 Dec. 2015).

Elisaus, P., Williams, G., Bourke, M., Clough, G., Harrison, A., & Verma, A. (2015).

Factors associated with the prevalence of adolescent binge drinking in the urban areas of Greater Manchester. *European Journal of Public Health*, *28*(1), 49–54. doi:10.1093/eurpub/ckv115.

Engelen, L., Bundy, A. C., Naughton, G., Simpson, J. M., Bauman, A., Ragen, J., & van der Ploeg, H. P. (2013). Increasing physical activity in young primary school children - it’s child’s play: a cluster randomised controlled trial. *Journal of Preventive Medicine*, *56*(5), 319–325. doi:10.1016/j.ypmed.2013.02.007.

Falaschetti, E., Mindell, J., Knott, C., & Poulter, N. (2014). Hypertension management in England: a serial cross-sectional study from 1994 to 2011. *The Lancet*, *383*,

1912–1919. doi:10.1016/S0140-6736(14)60688-7.

Filion, K. B., & Luepker, R. V. (2013). Cigarette smoking and cardiovascular disease: lessons from Framingham. *Journal of Global Heart*, *8*(1), 35–41. doi:10.1016/j.gheart.2012.12.005.

Fisher, J. D., & Fisher, W. A. (1992). Changing AIDS-risk behavior.<http://digitalcommons.uconn.edu/chip_docs/2> (accessed 21 Nov. 2015).

Fisher, J. D., & Fisher, W. A. (2000). Theoretical approaches to individual-level change in HIV risk behavior theoretical approaches to individual level change in HIV risk behavior. <http://digitalcommons.uconn.edu/chip_docs/4> (accessed 10

Nov. 2016).

Fisher, J. D., Fisher, W. A., Amico, K. R., & Herman, J. J. (2006). An information- motivation-behavioral Skills model of adherence to antiretroviral therapy*. Journal of Health Psychology. 25,* 462-473.

Fisher, W., Fisher, J., & Harman, J. (2003). The information-motivation-behavioral skill model: a general social psychological approach to understanding promoting health behavior. (Blackwell, Malden, Eds.). John Wiley & Sons Ltd. Page 82-

100.

Fisher, W., Sand, M., Lewis, W., & Boroditsky, R. (2000). Canadian menopause study-I: nderstanding women’s intentions to utilise hormone replacement therapy. *The European Menopause Journal*, *37*, 1–14. doi:10.1016/S0378-

5122(00)00163-8.

Fonseca, G., Bertolin, M., Gubert, M. B., & Da Silva, E. F. (2019). Effects of a nutritional intervention using pictorial representations for promoting knowledge and practices of healthy eating among Brazilian adolescents. *PLoS ONE*, *14*(3),

1–12. doi:10.1371/journal.pone.0213277

Fortuna, R. J., Nagel, A. K., Rose, E., Mccann, R., Teeters, J. C., Quigley, D. D., & Rocco, T. A. (2015). Effectiveness of a multidisciplinary intervention to improve hypertension control in an urban underserved practice. *Journal of the American Society of Hypertension*, *9*(12), 966–974. doi:10.1016/j.jash.2015.10.004.

Franks, A. L., Kelder, S. H., Dino, G. A., Horn, K. A., Gortmaker, S. L., Wiecha, J.

L., & Simoes, E. J. (2015). School nutrition and activity impact on well-being. (A. Hassan, Ed.). Ontario: Apple Academic Press. Page 147-162.

Gaal, L. F. Van, & Maggioni, A. P. (2013). Overweight, obesity, and outcomes: fat mass and beyond. *The Lancet*, *383*(9921), 935–936. doi:10.1016/S0140-

6736(13)62076-0.

Gabrhelik, R., Duncan, A., Miovsky, M., Furr-Holden, C. D. M., Stastna, L., & Jurystova, L. (2012). “Unplugged”: a school-based randomized control trial to prevent and reduce adolescent substance use in the Czech Republic. *Drug and Alcohol Dependence*, *124*(1–2), 79–87. doi:10.1016/j.drugalcdep.2011.12.010.

Gebremariam, M., Henjum, S., Terragni, L., & Torheim, L. (2016). Correlates of fruit, vegetable, soft drink, and snack intake among adolescents: the ESSENS study. *Food & Nutrition Research*, *60*, 1–9. doi:10.3402/fnr.v60.32512.

Gellert, C., Schoottker, B., Muller, H., Holleczek, B., & Brenner, H. (2013). Impact of smoking and quitting on cardiovascular outcomes and risk advancement periods among older adults. *European Journal of Epidemiology*, *28*(8), 649–658. doi:10.1007/s10654-013-9776-0.

George, G. M., Sharma, K. K., Ramakrishnan, S., & Gupta, S. K. (2013). A study of cardiovascular risk factors and its knowledge among school children of Delhi. *Indian Heart Journal*, *66*(3), 263–271. doi:10.1016/j.ihj.2014.03.003.

Ghana Health Service (GHS). (2015). 2014 annual report. Accra. [http://www.ghanahealthservice.org/ghs-category.php.cid=5](http://www.ghanahealthservice.org/ghs-category.php.cid) (accessed 21 Nov.

2015).

Ghana Health Service (GHS). (2017b). 2016 annual report. Accra.<http://ghanahealthservice.org/downloads/GHS_ANNUAL_REPORT_2016_n.p> df (accessed 21 Nov. 2018).

Ghana Health Service (GHS) (2017a). The health sector in Ghana: facts and figures

2017. <http://ghanahealthservice.org/downloads/FACTS+FIGURES_2017.pdf>

(accessed 21 Nov. 2018).

Ghana Statistical Service (GSS) (2009). Ghana demographic and health survey 2008. https:/[/www.dhsprogram.com/pubs/pdf/FR221/FR221.pdf](http://www.dhsprogram.com/pubs/pdf/FR221/FR221.pdf) (accessed 21 Nov.

2015).

Ghana Statistical Service (GSS). (2013). 2010 population & housing census: regional analytical report, Brong Ahafo Region. Accra.<http://www.statsghana.gov.gh/docfiles/2010phc/2010_PHC_Regional_Analytic> al_Reports_Brong_Ahafo_Region .pdf (accessed 21 Nov. 2015).

Ghana Statistical Service (2012). 2010 population & housing census: summary report of final results. Accra.<http://www.statsghana.gov.gh/docfiles/2010phc/Census2010_Summary_report_> of_final_results.pdf (accessed 21 Nov. 2015).

Glanz, K., Rimer, B., & Viswanath, K. (2008). Health behavior and health education:

theory, research, and practice (4th ed.). San Francisco: Jossey Bass. Page 11-50.

Gómez-Pardo, E., Fernández-Alvira, J. M., Vilanova, M., Haro, D., Martínez, R., Carvajal, I., et al. (2016). A comprehensive lifestyle peer group–based intervention on cardiovascular risk factors. *Journal of American College of Cardiology*, *67*(5), 479–485. doi:10.1016/j.jacc.2015.10.033

Gooding, H. C., Mcginty, S., Richmond, T. K., Gillman, M. W., & Field, A. E. (2014).

Hypertension awareness and control among young adults in the national longitudinal study of adolescent health. *Journal of General Internal Medicine*,

*29*(8), 1098–1104. doi:10.1007/s11606-014-2809-x.

Gorini, G., Carreras, G., Bosi, S., Tamelli, M., Monti, C., Storani, S., & Faggiano, F. (2014). Effectiveness of a school-based multi-component smoking prevention intervention: The LdP cluster randomized controlled trial. *Journal of Preventive Medicine*, *61*, 6–13. doi:10.1016/j.ypmed.2014.01.004.

Guerra, P. H., Nobre, M. R. C., Da Silveira, J. A. C., & Taddei, J. A. De A. C. (2013).

The effect of school-based physical activity interventions on body mass index: a meta-analysis of randomized trials. *Journal of Clinics, 68*(9), 1263–73. doi:10.6061/clinics/2013(09)14.

Guo, J., Lee, T., Liao, J., & Huang, C.-M. (2015). Prevention of illicit drug use through a school-based program: results of a longitudinal, cluster-randomized controlled trial. *Journal of Adolescent Health*, *56*(3), 314–322. doi:10.1016/j.jadohealth.2014.12.003.

Guthold, R., Cowan, M. J., Autenrieth, C. S., Kann, L., & Riley, L. M. (2010).

Physical activity and sedentary behavior among schoolchildren: a 34-country comparison. *Journal of Pediatrics*, *157*(1), 43–49.e1. doi:10.1016/j.jpeds.2010.01.019.

Hallal, P. C., Andersen, L. B., Bull, F. C., Guthold, R., Haskell, W., & Ekelund, U. (2012). Global physical activity levels: surveillance progress, pitfalls and prospects. *The Lancet*, *380*(9838), 247–257. doi:10.1016/S0140-6736(12)60646-

1.

Hambrick, D. (2007). The field of management’s devotion to theory: too much of a good thing? *Academy of Management Journal*, *50*(6), 1346–1352. doi:10.5465/AMJ.2007.28166119

Hammer, J., Fisher, J., Fiztgerald, P., & Fisher, W. (1996). When two heads aren’t better than one: AIDS risk behavior in college-age couples. *Journal of Applied Social Psychology*, *26*(5), 375–397. doi:10.1111/j.1559-1816.1996.tb01855.x.

Harder-lauridsen, N. M., Birk, N. M., Ried-larsen, M., Juul, A., & Andersen, L. B. (2014). A randomized controlled trial on a multicomponent intervention for overweight. *BMC Journal of Pediatrics*, *24*(273), 1–14.

He, F. J., Pombo-rodrigues, S., & Macgregor, G. A. (2014). Salt reduction in England from 2003 to 2011: its relationship to blood pressure, stroke and ischaemic heart disease mortality. *British Medical Journal*, *4*, 1–8. doi:10.1136/bmjopen-2013-

004549.

He, F. J., Wu, Y., Feng, X.-X., Ma, J., Ma, Y., Wang, H., & MacGregor, G. A. (2015).

School based education programme to reduce salt intake in children and their families (School-EduSalt): cluster randomised controlled trial. *British Medical Journal*, *350*, 1-8. doi:10.1136/bmj.h770.

Herouvi, D., Karanasios, E., Karayianni, C., & Karavanaki, K. (2013). Cardiovascular disease in childhood: the role of obesity. *European Journal of Pediatrics*, *172*(6),

721–732. doi:10.1007/s00431-013-1932-8.

Hiemstra, M., Ringlever, L., Otten, R., van Schayck, O. C. P., Jackson, C., & Engels, R. C. M. E. (2014). Long-term effects of a home-based smoking prevention program on smoking initiation: a cluster randomized controlled trial. *Journal of Preventive Medicine*, *60*, 65-70. doi:10.1016/j.ypmed.2013.12.012.

Holmes, M. V., Dale, C. E., Zuccolo, L., Silverwood, R. J., Guo, Y., Ye, Z., & Casas, J. P. (2014). Association between alcohol and cardiovascular disease: mendelian randomisation analysis based on individual participant data. *British Medical Journal*, *349*, 1-16. doi:10.1136/bmj.g4164.

Hormenu, T., Hagan Jnr, J., & Schack, T. (2018). Predictors of alcohol consumption among in- school adolescents in the Central Region of Ghana: a baseline information for developing cognitive-behavioural interventions. *PLoS ONE*,

*13*(11), 1–16. doi:10.1371/journal.pone.0207093.

Hout, M. C. V., Foley, M., McCormack, A., & Tardif, E. (2012). Teachers’ perspectives on their role in school-based alcohol and cannabis prevention. *International Journal of Health Promotion & Education*, *50*(6), 328–341. doi:10.1080/14635240.2012.735388.

Huang, C., Huang, J., Tian, Y., Yang, X., & Gu, D. (2014). Sugar sweetened beverages consumption and risk of coronary heart disease: a meta-analysis of prospective studies. *Journal of Atherosclerosis*, *234*(1), 11–16. doi:10.1016/j.atherosclerosis.2014.01.037.

Huang, C., Lee, S. O., Chang, E., Pang, H., & Chang, C. (2016). Androgen Receptor

(AR) in cardiovascular diseases. *Journal of Endocrinology*, *229*(1), 1–35.

Hung, L., Tidwell, D. K., Hall, M. E., Lee, M. L., Briley, C. A., & Hunt, B. P. (2015).

A meta-analysis of school-based obesity prevention programs demonstrates limited efficacy of decreasing childhood obesity. *Journal of Nutrition Research*,

*35*(3), 229–240. doi:10.1016/j.nutres.2015.01.002.

Hunter, D. J., & Reddy, K. S. (2013). Noncommunicable diseases. *New England*

*Journal of Medicine*, *369*(14), 1336–1343. doi:10.1056/NEJMra1109345.

Ikehara, S., Iso, H., Yamagishi, K., Kokubo, Y., Saito, I., Yatsuya, H., & Tsugane, S. (2013). Alcohol consumption and risk of stroke and coronary heart disease among Japanese women: the Japan public health center-based prospective study. *Journal of Preventive Medicine*, *57*(5), 505–510. doi:10.1016/j.ypmed.2013.07.003.

Institute of Medicine. (2010). Promoting cardiovascular health in the developing world: a critical challenge to achieve global health. Washington, DC: The national academic press.Page 100-137.

Isensee, B., Hansen, J., Maruska, K., & Hanewinkel, R. (2014). Effects of a school- based prevention programme on smoking in early adolescence: a 6-month follow-up of the “Eigenstandig Werden” cluster randomised trial. *British Medical Journal*, *4*(1), 1-8. doi:/10.1136/bmjopen-2013-004422.

Iwelunmor, J., Blackstone, S., Veira, D., Nwaozuru, U., Airhihenbuwa, C., Munodawafa, D., et al. (2016). Toward the sustainability of health interventions implemented in sub-Saharan Africa: a systematic review and conceptual framework. *Journal of Implementation Science*, *11*(43), 1–27. doi:10.1186/s13012-016-0392-8.

Jakovljevic, D. G. (2018). Physical activity and cardiovascular aging: physiological and molecular insights. *Journal of Experimental Gerontology*, *109*, 67–74. doi:10.1016/j.exger.2017.05.016.

Jamal, A., Homa, D. M., O’Connor, E., Babb, S. D., Caraballo, R. S., Singh, T., & King, B. A. (2015). Current cigarette smoking among adults-United States*, 2005-*

*2014* (Vol. 64), 1233-1259. https:/[/www.cdc.gov/mmwr/preview/mmwrhtml/mm6444a2.htm](http://www.cdc.gov/mmwr/preview/mmwrhtml/mm6444a2.htm) (accessed 22

Nov. 2016).

Juonala, M., Viikari, J. S. A., Kahonen, M., Taittonen, L., Laitinen, T., Hutri-Kahonen, N., & Raitakari, O. T. (2010). Life-time risk factors and progression of carotid atherosclerosis in young adults: the cardiovascular risk in Young Finns study. *European Heart Journal*, *31*(14), 1745–1751. doi:10.1093/eurheartj/ehq141.

Kain, J., Concha, F., Moreno, L., & Leyton, B. (2014). School-based obesity prevention intervention in chilean children: effective in controlling, but not reducing obesity. *Journal of Obesity*, *2014*, 1–8. doi:10.1155/2014/618293.

Kanjee, Z., Amico, K. R., Li, F., Mbolekwa, K., Moll, A. P., & Friedland, G. (2012).

Tuberculosis infection control in a high drug-resistance setting in rural South Africa: information, motivation, and behavioral skills. *Journal of Infection and Public Health*, *5*(1), 67–81. doi:10.1016/j.jiph.2011.10.008.

Katsiki, N., Papadopoulou, S. K., Fachantidou, A. I., & Mikhailidis, D. P. (2013).

Smoking and vascular risk: are all forms of smoking harmful to all types of vascular disease? *Public Health Journal*, *127*(5), 435–441. doi:10.1016/j.puhe.2012.12.021

Kaya, C., Blake, J., & Chan, F. (2015). Peer-mediated interventions with elementary and secondary school students with emotional and behavioural disorders: a literature review. *Journal of Research and Special Educational Needs*, *15*(2),

120–129. doi:10.1111/1471-3802.12029.

Kelly, A. S., Barlow, S. E., Rao, G., Inge, T. H., Hayman, L. L., Steinberger, J., & Daniels, S. R. (2013). Severe obesity in children and adolescents: identification, associated health risks, and treatment approaches. *Journal of Circulation*,

*2013*(128), 1689-1712. doi:10.1161/CIR.0b013e3182a5cfb3.

Khambalia, A. Z., Dickinson, S., Hardy, L. L., Gill, T., & Baur, L. A. (2012). A synthesis of existing systematic reviews and meta-analyses of school-based behavioural interventions for controlling and preventing obesity. *Obesity Reviews Journal*, *13*, 214–233. doi:10.1111/j.1467-789X.2011.00947.x.

Khumros, W., Vorayingyong, A., Suppapitiporn, S., Rattananupong, T., & Lohsoonthorn, V. (2019). Effectiveness of modified health belief model-based intervention to reduce body mass index for age in overweight junior high school students in Thailand. *Journal of Health Research*, *33*(2), 162–172. doi:10.1108/JHR-08-2018-0065.

Kim, H. (2013). Statistical notes for clinical researchers: assessing normal distribution

(2) using skewness and kurtosis. *Journal of Restorative Dentistry & Endodontics*,

*38*(1), 52–54. doi:10.5395/rde.2013.38.1.52.

Kolber, M. R. (2014). Family history of cardiovascular disease. *Journal of Canadian*

*Family Physician*, *60*(11), 1016.

Kowalski, K. C., Crocker, P. R. E., & Donen, R. M. (2004). The physical activity questionnaire for older children (PAQ-C) and adolescents (PAQ-A) manual. College of Kinesiology, University of Saskatchewan. https:/[/www.researchgate.net/publication/228441462](http://www.researchgate.net/publication/228441462) (accessed 21 Nov. 2015).

Kuciene, R., & Dulskiene, V. (2014). Associations of short sleep duration with prehypertension and hypertension among Lithuanian children and adolescents: a cross-sectional study. *BMC Public Health*, *14*(255), 1–8. doi:10.1186/1471-

2458-14-255.

Kumah, D. B., Akuffo, K. O., Abaka-Cann, J. E., Affram, D. E., & Osae, E. A. (2015).

Prevalence of overweight and obesity among students in the Kumasi Metropolis.

*Journal of Nutrition and Metabolism*, *2015*, 1–4. doi:10.1155/2015/613207.

Labarthe, D. R. (2011). Epidemiology and prevention of cardiovascular diseases: a global challenge (2nd ed). Sudbury, Massachusetts: Jones and Bartlett publishers. Page 155-201.

Lammers, J., Goossens, F., Conrod, P., Engels, R., Wiers, R. W., & Kleinjan, M. (2015). Effectiveness of a selective intervention program targeting personality risk factors for alcohol misuse among young adolescents: results of a cluster randomized controlled trial. *Society for the Study of Addiction*, *110*, 1101–1109. doi:10.1111/add.12952.

Lao, C., Chan, Y., Tong, H. H., & Chan, A. (2015). Prevalence, awareness and control of cardiovascular risk factors in a low-income population in Macao, China. *Journal of Public Health Research, 5*(2), 50–57. doi:10.5923/j.phr.20150502.02.

Lavelle, H. V., MacKay, D. F., & Pell, J. P. (2012). Systematic review and meta- analysis of school-based interventions to reduce body mass index. *Journal of Public Health*, *34*(3), 360–369. doi:10.1093/pubmed/fdr116.

Lemeshow, S., Hosmer, D. W. J., Klar, J., & Lwanga, S. K. (1990). Adequacy of sample size in health studies. John Wiley & Sons Ltd. Page 99-147.

Li, J., & Siegrist, J. (2012). Physical activity and risk of cardiovascular disease-a meta- analysis of prospective cohort studies. *International Journal of Environmental Research and Public Health*, *9*(2), 391–407. doi:10.3390/ijerph9020391.

Li, K., Yao, C., Di, X., Yang, X., Dong, L., Xu, L., & Zheng, M. (2016). Smoking and risk of all-cause deaths in younger and older adults. *Journal of Medicine*, *95*(3),

1-15. doi:10.1097/MD.0000000000002438.

Lierman, L., Kasprzyk, D., & Benoliel, J. (1991). Understanding adherence of breast self-examination in older women. *Western Journal of Nursing Research*, *13*(1),

46–66. doi:10.1177/019394599101300104.

Liyanage, L., Lee, N. J., Cook, T., Herrmann, H. C., Jagasia, D., & Han, Y. (2016).

The impact of gender on cardiovascular system calcification in very elderly patients with severe aortic stenosis. *International Journal of Cardiovascular Imaging*, *32*, 173–179. doi:10.1007/s10554-015-0752-5.

Lonsdale, C., Rosenkranz, R. R., Peralta, L. R., Bennie, A., Fahey, P., & Lubans, D.

R. (2013a). A systematic review and meta-analysis of interventions designed to increase moderate-to-vigorous physical activity in school physical education lessons. *Journal of Preventive Medicine*, *56*(2), 152–161. doi:10.1016/j.ypmed.2012.12.004.

Lonsdale, C., Rosenkranz, R. R., Sanders, T., Peralta, L. R., Bennie, A., Jackson, B.,

& Lubans, D. R. (2013b). A cluster randomized controlled trial of strategies to increase adolescents’ physical activity and motivation in physical education: results of the Motivating Active Learning in Physical Education (MALP) trial. *Journal of Preventive Medicine*, *57*(5), 696–702. doi:10.1016/j.ypmed.2013.09.003.

Lu, Y., Hajifathalian, K., Ezzati, M., Woodland, M., Rimm, E. B., & Danaei, G. (2014). Metabolic mediators of the effects of body-mass index, overweight, and obesity on coronary heart disease and stroke: a pooled analysis of 97 prospective cohorts with 1·8 million participants. *The Lancet*, *383*, 970–983. doi:10.1016/S0140-6736(13)61836-X.

Maas, A. H. E. M., & Appelman, Y. E. A. (2010). Gender differences in coronary heart disease. *Netherlands Heart Journal*, *18*(12), 598–603. doi:10.1007/s12471-

010-0841-y.

Mahmood, S. S., Levy, D., Vasan, R. S., & Wang, T. J. (2014). The framingham heart study and the epidemiology of cardiovascular disease: a historical perspective. *The Lancet*, *383*(9921), 999–1008. doi:10.1016/S0140-6736(13)61752-3.

Mamudu, H., & Veeranki, S. (2013). Tobacco use among school-going adolescents (11–17 years) in Ghana. *Nicotine and Tobacco Research*, *15*(8), 1355–1364. doi:10.1093/ntr/nts269.

Mayega, R. W., Makumbi, F., Rutebemberwa, E., Peterson, S., Ostensen, C.-G., Tomson, G., & Guwatudde, D. (2012). Modifiable socio-behavioural factors associated with overweight and hypertension among persons aged 35 to 60 years in Eastern Uganda. *PLoS ONE*, *7*(10), 1–9. doi:10.1371/journal.pone.0047632.

Mcisaac, J. D., Hernandez, K. J., Kirk, S. F. L., & Curran, J. A. (2016). Interventions to support system-level implementation of health promoting schools: a scoping review. *International Journal of Environmental Research and Public Health*,

*13*(200), 1–24. doi:10.3390/ijerph13020200.

Mei, H., Xiong, Y., Xie, S., Guo, S., Li, Y., Guo, B., & Zhang, J. (2016). The impact of long-term school-based physical activity interventions on body mass index of primary school children –a meta-analysis of randomized controlled trials. *BMC Public Health*, *16*(205), 1–12. doi:10.1186/s12889-016-2829-z.

Mendis, S., Lindholm, L. H., Anderson, S. G., Alwan, A., Koju, R., Onwubere, B. J.

C., & Heagerty, A. (2011). Total cardiovascular risk approach to improve efficiency of cardiovascular prevention in resource constrain settings. *Journal of Clinical Epidemiology*, *64*(12), 1451–1462. doi:10.1016/j.jclinepi.2011.02.001.

Mendonca, R. D. D., Marc, A., Gea, A., Fuente-arrillaga, C. De, Martinez-gonzalez, M. A., Cristine, A., & Bes-rastrollo, M. (2016). Ultraprocessed food consumption and risk of overweight and obesity: The University of Navarra Follow-Up (SUN) cohort study. *American Journal of Clinical Nutrition*, *104*(5), 1433–1440. doi:10.3945/ajcn.116.135004.

Midford, R., Mitchell, J., Lester, L., Cahill, H., Foxcroft, D., Ramsden, R., & Pose, M. (2014). Preventing alcohol harm: early results from a cluster randomise, controlled trial in Victoria, Australia of comprehensive harm minimisation school drug education. *International Journal of Drug Policy*, *25*(1), 142–150. doi:10.1016/j.drugpo.2013.05.012.

Ministry of Education Ghana (MOE). (2019). Education system in Ghana. [http://moe.gov.gh/index.php/abou](http://moe.gov.gh/index.php/)t-us/ (accessed 9 Aug. 2019).

Ministry of Health Ghana (MOH). (2012). Strategy for the management, prevention and control of chronic non-communicable diseases in Ghana 2012-2016. Accra. https:/[/www.mindbank.info/item/1933](http://www.mindbank.info/item/1933) (accessed 21 Nov. 2015).

Misovich, S., Fisher, J., & Fisher, W. (1996). The perceived AIDS-preventive utility of knowing one’s partner well: a public health dictum and individual’s risky sexual behavior. *Canadian Journal of Human Sexuality*, *5*(2), 83–90.

Misovich, S., Martinez, T., Fisher, J., Bryan, A., & Catapano, N. (2003). Predicting breast self-examination: a test of the Information-Motivation-Behavioral Skills model. *Journal of Applied Social Psychology*, *33*(4), 775–790. doi:10.1111/j.1559-1816.2003.tb01924.x.

Mogre, V., Aneyire, E. S., & Gyamfi, E. K. (2013). Physical activity and BMI status of school-age children in Tamale, Northern Ghana. *Pakistan Journal of Nutrition*,

*12*(5), 484–490.

Mohammed, M., Matthijs, S., Alotaiby, F. F., de Vries, N., & de Vries, H. (2016).

Effects of a randomized controlled trial to assess the six-months effects of a school based smoking prevention program in Saudi Arabia. *Journal of Preventive Medicine*, *90*, 100–106. doi:10.1016/j.ypmed.2016.06.032.

Moher, D., Hopewell, S., Schulz, K. F., Montori, V., Gøtzsche, P. C., Devereaux, P.

J., & Altman, D. G. (2010). CONSORT 2010 explanation and elaboration: updated guidelines for reporting parallel group randomised trials. *British Medical Journal*, *10*(1), 1-14. doi: 10.1136/bmj.c869.

Moore, G. F., Littlecott, H. J., Fletcher, A., Hewitt, G., & Murphy, S. (2016).

Variations in schools’ commitment to health and implementation of health improvement activities: a cross-sectional study of secondary schools in Wales. *BMC Public Health*, *16*(138), 1–11. doi:10.1186/s12889-016-2763-0.

Moran, A. E., Roth, G. A., Narula, J., & Mensah, G. A. (2014). 1990-2010 global cardiovascular disease atlas. *Global Heart*, *9*(1), 3–16. doi:10.1016/j.gheart.2014.03.1220.

Morano, M., Rutigliano, I., Rago, A., Pettoello-Mantovani, M., & Campanozzi, A. (2016). A multi-component school-initiated obesity intervention to promote healthy lifestyles in children. *Nutrition*, *32*(10), 1075–80. doi:10.1016/j.nut.2016.03.007.

Movva, R., & Figueredo, V. M. (2013). Alcohol and the heart: To abstain or not to abstain? *International Journal of Cardiology*, *164*(3), 267–276. doi:10.1016/j.ijcard.2012.01.030.

Mozaffarian, D., Benjamin, E. J., Go, A. S., Roger, V. L., Berry, J. D., Borden, W. B.,

& Turner, M. B. (2015). Executive summary: heart disease and stroke statistics-

-2015 update: a report from the American Heart Association.<http://circ.ahajournals.org/cgi/doi/10.1161/>CIR.0b013e318282ab8f (accessed

21 Nov. 2016).

Muka, T., Oliver-Williams, C., Kunutsor, S., Laven, J. S. E., Fauser, B. C. J. M., Chowdhury, R., & Franco, O. H. (2016). Association of age at onset of menopause and time since onset of menopause with cardiovascular outcomes, intermediate vascular traits, and all-cause mortality: a systematic review and meta-analysis. *JAMA Cardiology*, *1*(7), 767–776. doi:10.1001/jamacardio.2016.2415.

Mukamana, O., & Johri, M. (2016). What is known about school-based interventions for health promotion and their impact in developing countries? a scoping review of the literature. *Health Education Research*, *31*(5), 587–602. doi:10.1093/her/cyw040.

Muncan, B. (2018). Cardiovascular disease in racial/ethnic minority populations: illness burden and overview of community-based interventions. *Public Health Reviews*, *39*(32), 1–11. doi:10.1186/s40985-018-0109-4.

Myers, J., McAuley, P., Lavie, C. J., Despres, J. P., Arena, R., & Kokkinos, P. (2015).

Physical activity and cardiorespiratory fitness as major markers of cardiovascular risk: their independent and interwoven importance to health status. *Progress in Cardiovascular Diseases Journal*, *57*(4), 306–314. doi:10.1016/j.pcad.2014.09.011.

Nakamura, K., Fuster, J. J., & Walsh, K. (2014). Adipokines: a link between obesity and cardiovascular disease. *Journal of Cardiology*, *63*(4), 250–259. doi:10.1016/j.jjcc.2013.11.006.

National Health Service. (2015). Cardiovascular disease overview.<http://www.nhs.uk/Conditions/cardiovascular-disease/Pages/Introduction.aspx> (accessed 18 Dec. 2015).

National Institute of Health. (2005). Fourth report on diagnosis, evaluation, and treatment of high blood pressure in children and adolescents. [http://www.nhlbi.nih.gov.](http://www.nhlbi.nih.gov) (accessed 21 Nov. 2015).

Nielsen, M., Andersson, C., Gerds, T. A., Andersen, P. K., Jensen, T. B., Køber, L.,

& Torp-pedersen, C. (2013). Familial clustering of myocardial infarction in first- degree relatives: a nationwide study. *European Heart Journal*, *34*, 1198–1203. doi:0.1093/eurheartj/ehs475.

Nti, C. A. (2008). Household dietary practices and family nutritional status in rural Ghana. *Journal of Nutrition Research and Practice*, *2*(1), 35–40. doi:10.4162/nrp.2008.2.1.35.

Nyawornota, V. K., Aryeetey, R., Bosomprah, S., & Aikins, M. (2013). An exploratory study of physical activity and over-weight in two senior high schools in the Accra Metropolis. *Ghana Medical Journal*, *47*(4), 197–203.

Obirikorang, Y., Obirikorang, C., Anto, E. O., Acheampong, E., Dzah, N., Akosah, C.

N., & Nsenbah, E. B. (2016). Knowledge and lifestyle-associated prevalence of obesity among newly diagnosed type II diabetes mellitus patients attending diabetic clinic at komfo anokye teaching hospital, Kumasi, Ghana: a hospital- based cross-sectional study. *Journal of Diabetes Research*, *2016*, 1–10. doi:10.1155/2016/9759241.

Ofori-Asenso, R., & Garcia, D. (2015). Cardiovascular diseases in Ghana within the context of globalization. *Journal of Cardiovascular Diagnosis and Therapy,*

*6*(1)*,* 67–77. doi:10.3978/j.issn.2223-3652.2015.09.02.

Okubo, Y., Sairenchi, T., Irie, F., & Yamagishi, K. (2014). Association of alcohol consumption with incident hypertension among middle-aged and older japanese population: the Ibarakai Prefectural Health Study (IPHS). *Journal of American Heart Association*, *63*, 41–47. doi:10.1161/hypertensionaha.113.01585.

Oppong Asante, K., Meyer-Weitz, A., & Petersen, I. (2014). Substance use and risky sexual behaviours among street connected children and youth in Accra, Ghana. *Substance Abuse Treatment, Prevention, and Policy*, *9*(1), 1-9. doi:10.1186/1747-597x-9-45.

Oppong Asante, K., & Kugbey, N. (2019). Alcohol use by school-going adolescents in Ghana: prevalence and correlates. *Journal of Mental Health & Prevention*, *13*,

75–81. doi:10.1016/j.mhp.2019.01.009.

Owen, N., Healy, G. N., Matthews, C. E., & Dunstan, D. W. (2012). Too much sitting: the population-health science of sedentary behaviour. *Exercise and Sport Sciences Reviews Journal*, *38*(3), 105–113. doi:10.1097/JES.0b013e3181e373a2.

Owusu, A. A., Ankrah, M. Y., Boateng, G. P., & Anteh, G. (1995). Food weight/handy measures tables (Ghana). The DREYFUS Health Foundation ''problem-solving for better health'' programm, Accra, Ghana. Page 1-31 <http://www.dreyfus> foundation-Ghana.com.

Patnode, C. D., Connor, E. O., Whitlock, E. P., Perdue, L. A., Soh, C., & Hollis, J. (2013). Primary care-relevant interventions for tobacco use prevention and cessation in children and adolescents: a systematic evidence review for the US preventive services task force. *Annals of Internal Medicne Journal*, *158*(4), 253–

260. doi: 10.7326/0003-4819-158-4-201302190-00580.

Pearson, N., & Biddle, S. J. H. (2011). Sedentary behavior and dietary intake in children, adolescents, and adults: a systematic review. *American Journal of Preventive Medicine*, *41*(2), 178–188. doi:10.1016/j.amepre.2011.05.002.

Pérez-López, F., Larrad-Mur, L., Kallen, A., Chedraui, P., & Taylor, H. (2011).

Gender differences in cardiovascular disease: hormonal and biochemical influences. *Reproductive Science*, *17*(6), 511–531. doi:10.1177/1933719110367829.

Perk, J., Helmut, G., Graham, I., Reiner, Z., & Verschuren, W. M. M. (2012).

European guidelines on cardiovascular disease prevention in clinical practice.

*European Heart Journal*, *33*, 1635–1701. doi:10.1093/eurheartj/ehs092.

Pickering, T. G., Hall, J. E., Appel, L. J., Falkner, B. E., Graves, J., Hill, M. N., & Roccella, E. J. (2005). Recommendations for blood presure measurement in humans and experimental animals. part 1: blood pressure measurement in humans. *Hypertension Journal*, *45*, 142–161. doi:10.1161/01.HYP.0000150859.47929.8e.

Ploeg, K. A.V., Maximova, K., Mcgavock, J., Davis, W., & Veugelers, P. (2014). Do school-based physical activity interventions increase or reduce inequalities in health? *Social Science & Medicine*, *112*, 80–87. doi:10.1016/j.socscimed.2014.04.032.

Popkin, B. M., & Slining, M. M. (2013). New dynamics in global obesity facing low- and middle-income countries. *Obesity Reviews Journal*, *14*, 11–20. doi:10.1111/obr.12102.

Rampal, L., Rampal, S., Azhar, M. Z., & Rahman, A. R. (2008). Prevalence, awareness, treatment and control of hypertension in Malaysia: a national study of 16,440 subjects. *Journal of Public Health*, *122*(1), 11–18. doi:10.1016/j.puhe.2007.05.008.

Rampal, S., Rampal, L., Rahmat, R., Azhar Md Zain, Yee Guan Yap, Mohamed, M.,

& Taha, M. (2009). Variation in the prevalence, awareness, and control of diabetes in a multiethnic population: a nationwide population study in Malaysia. *Asia-Pacific Journal of Public Health*, *22*(2), 194–202. doi:10.1177/1010539509334816.

Rapsomaniki, E., Timmis, A., George, J., Pujades-rodriguez, M., Shah, A. D., Denaxas, S., & Caulfi, M. J. (2014). Blood pressure and incidence of twelve cardiovascular diseases: lifetime risks, healthy life-years lost, and age-specifi c associations in 1·25 million people. *The Lancet*, *383*, 1899–1911. doi:10.1016/S0140-6736(14)60685-1.

Reremoana F. T., Jonathan B. D., Ambler, A., Hogan, S., Sandhya W. C., Michael J.A. et al. (2015). Childhood to early-midlife systolic blood pressure trajectories early-life predictors, effect modifiers, and adult cardiovascular outcomes. *Epidermiology and Population Journal*, *66*(6), 1108-1115. doi:10.1161/Hypertensionaha.115.05831.

Robertson, W., Murphy, M., & Johnson, R. (2016). Evidence base for the prevention and management of child obesity. *Journal of Paediatrics and Child Health*, *12*,

1–7. doi:10.1016/j.paed.2015.12.009.

Ruijter, H. M. Den, & Pasterkamp, G. (2015). Gender and cardiovascular disease.

*PanVascular Medicine Journal, 10*, 1939–1951. doi:10.1007/ 978-3-642-37078-

6_244.

Sanuade, O., Anarfi, J., Aikins, A., & Koram, K. (2014). Patterns of cardiovascular disease mortality in Ghana: a 5-year review of autopsy cases at Korle-Bu Teaching Hospital. *European PMC Journal*, *24*(1), 55–59.

Sanuade, O., Boatemaa, S., & Kushitor, M. (2018). Hypertension prevalence, awareness, treatment and control in Ghanaian population: evidence from the Ghana demographic and health survey. *PLoS ONE*, *13*(11), 1–18. doi:10.1371/journal.pone.0205985.

Saraf, D. S., Gupta, S. K., Pandav, C. S., Nongkinrih, B., Kapoor, S. K., Pradhan, S.

K., & Krishnan, A. (2015). Effectiveness of a school based intervention for prevention of non-communicable diseases in middle school children of rural north india: a randomized controlled trial. *Indian Journal of Pediatrics*, *82*(4),

354–362. doi:10.1007/s12098-014-1562.

Sawyer, S. M., Afi, R. A., Bearinger, L. H., Blakemore, S., Dick, B., Ezeh, A. C., & Patton, G. C. (2012). Adolescence: a foundation for future health. *The Lancet*,

*379*, 1630–1640. doi:10.1016/S0140-6736(12)60072-5.

Scaglioni, S., De Cosmi, V., Ciappolino, V., Parazzini, F., Brambilla, P., & Agostoni,

C. (2018). Factors influencing children’s eating behaviours. *Journal of Nutrients*,

*10*, 1–17. doi:10.3390/nu10060706.

Schutter, A., Lavie, C. J., & Milani, R. V. (2014). The impact of obesity on risk factors and prevalence and prognosis of coronary heart disease—the obesity paradox. *Journal of Progress in Cardiovascular Diseases*, *56*(4), 401–408. doi:10.1016/j.pcad.2013.08.003.

Shahid, S. M., & Bishop, K. S. (2019). Comprehensive approaches to improving nutrition: future prospects. *Journal of Nutrients*, *11*(8), 1–19. doi:10.3390/nu11081760.

Shakeshaft, A., Doran, C., Petrie, D., Breen, C., Havard, A., Abudeen, A., & Sanson- Fisher, R. (2014). The effectiveness of community action in reducing risky alcohol consumption and harm: a cluster randomised controlled trial. *PLoS ONE*,

*11*(3), 1-14 doi:10.1371/journal.pmed.1001617.

Sharma, B., Kim, H. Y., & Nam, E. W. (2018). Effects of school-based health promotion intervention on health behaviors among school adolescents in north Lima and Callao, Peru. *Journal of Lifestyle Medicine*, *8*(2), 60–71. doi:10.15280/jlm.2018.8.2.60.

Shrestha, R., & Copenhaver, M. (2015). Long-term effects of childhood risk factors on cardiovascular health during adulthood. *Clinical Medicine Review of Vascular Health*, *7*, 1–5. doi:10.4137/CMRVH.S29964.

Shrivastava, U., & Misra, A. (2015). Need for ethnic-specific guidelines for prevention, diagnosis, and management of type 2 diabetes in South asians. *Diabetes Technology Therapy*, *17*(6), 435–439. doi:10.1089/dia.2014.0213.

Simane, B., & Zaitchik, B. F. (2014). The sustainability of community-based adaptation projects in the blue nile highlands of Ethiopia. *Journal of Sustainability*, *6*, 4308–4325. doi:10.3390/su6074308.

Singh, G. K., Siahpush, M., Azuine, R. E., & Williams, S. D. (2015). Widening socioeconomic and racial disparities in cardiovascular disease mortality in the United States, 1969-2013. *International Journal of MCH and AIDS*, *3*(2), 106–

118.

Skinner, A. C., & Skelton, J. A. (2014). Prevalence and trends in obesity and severe obesity among children in the United States, 1999-2012. *JAMA*, *168*(6), 561–

566. doi:10.1001/jamapediatrics.2014.21.

Smith, S. C., Collins, A., Ferrari, R., Holmes, D. R., Logstrup, S., McGhie, D. V., & Zoghbi, W. A. (2012). Our time: a call to save preventable death from cardiovascular disease (heart disease and stroke). *European Heart Journal*, *33*(23), 2910–2916. doi:10.1093/eurheartj/ehs313.

Snijder, M. B., Agyemang, C., Peters, R. J., Stronks, K., Ujcic-voortman, J. K., & Valkengoed, I. G. M. Van. (2017). Case finding and medical treatment of type 2 diabetes among different ethnic minority groups: the HELIUS Study. *Journal of Diabetes Research*, *2017*, 1–8. doi:10.1155/2017/9896849.

Soler, E. P., & Ruiz, V. C. (2010). Epidemiology and risk factors of cerebral ischemia and ischemic heart diseases: similarities and differencesitle. *Current Cardiology Reviews*, *6*(3), 138–149. doi:10.2174/157340310791658785.

Sonnino, R. (2016). Health care leadership development and training: progress and pitfalls. *Journal of Healthcare Leadership*, *8*, 19–29.

Spagnolo, A., Giussani, M., Ambruzzi, A. M., Bianchetti, M., Maringhini, S., Matteucci, M. C., & Strambi, M. (2013). Focus on prevention, diagnosis and treatment of hypertension in children and adolescents. *Italian Journal of Pediatrics*, *39*(20), 1–18. doi:10.1186/1824-7288-39-20.

Strøm, H. K., Adolfsen, F., Fossum, S., Kaiser, S., & Martinussen, M. (2014).

Effectiveness of school-based preventive interventions on adolescent alcohol use: a meta-analysis of randomized controlled trials. *Substance Abuse Treatment, Prevention, and Policy*, *9*(48) 1–11. doi: 0.1186/1747-597x-9-48.

Suchert, V., Isensee, B., Sargent, J., Weisser, B., & Hanewinkel, R. (2015).

Prospective effects of pedometer use and class competitions on physical activity in youth: a cluster-randomized controlled trial. *Preventive Medicine*, *81*, 399–

404. doi:1016/j.ypmed.2015.10.002.

Sultana, N. (2017). Nutritional awareness among the parents of primary school going children. *Saudi Journal of Humanities and Social Sciences*, *2*, 708–725. doi.org/10.21276/sjhss.

Sung, M. M. Y., & Dyck, J. R. B. (2012). Age-related cardiovascular disease and the beneficial effects of calorie restriction. *Heart Failure Reviews*, *17*, 707–719. doi:10.1007/s10741-011-9293-8.

Sutherland, R., Campbell, E., Lubans, D. R., Morgan, P. J., Okely, A. D., Nathan, N.,

& Wiggers, J. (2015). ‘Physical Activity 4 Everyone’ school-based intervention to prevent decline in adolescent physical activity levels: 12 month (mid- intervention) report on a cluster randomised trial. *British Journal of Sports Medicine*, *50* (8), 1–10. doi:10.1136/bjsports-2014-094523.

Tabachnick, B. G., & Fidell, L. S. (2007). Using multivariate statistics. (S. Hartman

& T. Felser, Eds.) (5th ed.). Boston, USA: Pearson Education, Inc. Page 33-61.

Tabei, S. M. B., Senemar, S., Saffari, B., Ahmadi, Z., & Haqparast, S. (2014). Non- modifiable factors of coronary artery stenosis in late onset patients with coronary artery disease in southern Iranian population. *Journal of Cardiovascular and Thoracic Research*, *6*(1), 51–55. doi:10.5681/jcvtr.2014.010.

Tarp, J., Jespersen, E., Møller, N. C., Klakk, H., Wessner, B., Wedderkopp, N., & Bugge, A. (2018). Long-term follow-up on biological risk factors, adiposity, and cardiorespiratory fitness development in a physical education intervention: a natural experiment (CHAMPS-study DK). *BMC Public Health*, *18*(605), 1–14. doi:10.1186/s12889-018-5524-4 research.

Tavakol, M., & Dennick, R. (2011). Making sense of Cronbach’s alpha. *International*

*Journal of Medical Education*, *2*, 53–55. doi:10.5116/ijme.4dfb.8dfd.

Thapa, S., Martinez, P., & Clausen, T. (2014). Depression and its correlates in South Africa and ghana among people aged 50 and above: findings from the WHO study on global ageing and adult health. *Journal of Psychiatry*, *17*(6), 10–15. doi:10.4172/1994-8220.1000167.

The LifeWindows Team. (2006). The LifeWindows motivation-information- behavioral skills art adherence questionnaire (LW-IMB-AAQ). Centre for health, intervention, and prevention. university of Connecticut.<http://chipcontent.chip.uconn.edu/chipweb/documents/Research/F_LWIMBAR> TQuestionnaire.pdf (accessed 21 Nov. 2015).

The tobacco atlas country fact sheet, Ghana. (2015).<http://www.tobaccoatlas.org/country-data/ghana/> (accessed 21 Nov. 2016).

Thomas, R. E., Baker, P., & Thomas, B. C. (2016). Family-based interventions in preventing children and adolescents from using tobacco: a systematic review and meta-analysis. *Journal of Academic Pediatrics, 16(5),* 419-426. doi:10.1016/j.acap.2015.12.006.

Thompson, M., Dana, T., Bougatsos, C., Blazina, I., & Norris, S. L. (2013). Screening for hypertension in children and adolescents to prevent cardiovascular disease. *American Academy of Padiatrics*, *131*(3), 490–525. doi:10.1542/peds.2012-

3523.

Tripodi, S. J., Bender, K., Litschge, C., & Vaughn, M. G. (2010). Interventions for reducing adolescent alcohol abuse. *Journal of American Medical Association*,

*164*(1), 85–91. doi:10.1001/archpediatrics.2009.235.

Verschuren, W. M. M. (2012). Diet and cardiovascular disease. *Journal of Current*

*Cardiology Reports*, *14*(6), 701–708. doi:10.1007/s11886-012-0318-2.

Walugembe, D. R., Sibbald, S., Ber, M. J. L., & Kothari, A. (2019). Sustainability of public health interventions: where are the gaps? *Health Research Policy and Systems Journal*, *17*(8), 1–7. doi:10.1186/s12961-018-0405-y.

Wang, M., & Wang, L. (2018). Teaching games for understanding intervention to promote physical activity among secondary school students. *BioMed Research International*, *2018*, 1–11. doi:10.1155/2018/3737595.

Wenger, N. K., Ouyang, P., Miller, V. M., & Merz, N. B. (2016). Strategies and methods for clinical scientists to study sex-specific cardiovascular health and disease in women. *Journal of American College of Cardiology*, *67*(18), 18–20. doi:10.1016/j.jacc.2016.03.504.

Whelton, P. K. (2015). The elusiveness of population-wide high blood pressure control. *Annual Review of Public Health Journal*, *36*, 109–130. doi:10.1146/annurev-publhealth-031914-122949.

Whetten, D. (1989). What constitutes a theoretical contribution? *Academy of*

*Management Review*, *14*(4), 490–495. doi:10.5465/AMR.1989.4308371.

Williams, S., Kimble, D., Covell, N., Weiss, L., Newton, K., Fisher, J., & Fisher, W. (1992). College students use implicit personality theory instead of safer sex. *Journal of Applied Social Psychology*, *22*(12), 921–933.

Woodgate, R. L., & Sigurdson, C. M. (2015). Building school-based cardiovascular health promotion capacity in youth: a mixed methods study. *BMC Public Health*,

*15*(421), 1–11. doi:10.1186/s12889-015-1759-5.

World Health Organization. (2007). BMI-for-Age (15-19years).<http://www.who.int/growthref/who2007_bmi_for_age/en/> (accessed 20 Nov.

2016).

World Health Organization. (2010). Global recommendations on physical activity for healt[h.http://apps.who.int/iris/bitstream/10665/44399/1/9789241599979_eng.pd](http://apps.who.int/iris/bitstream/10665/44399/1/9789241599979_eng.pd) f (accessed 21 Nov. 2015).

World Health Organization. (2011b). A prioritized research agenda for prevention and control of noncommunicable diseases, World Health Organization, Geneva.<http://apps.who.int/iris/bitstream/10665/44569/1/9789241564205_eng.pdf> (accessed 21 Nov. 2015).

World Health Organization. (2011a). Global status report on noncommunicable diseases 2010, Geneva.<http://whqlibdoc.who.int/publications/2011/9789240686458_eng.pdf> (accessed

21 Nov. 2015).

World Health Organization. (2014). Global status report on noncommunicable diseases 2014 “Attaining the nine global noncommunicable diseases targets; a shared responsibility.” Geneva. Retrieved fr[om www.who.int](http://www.who.int) (accessed 21 Nov.

2015).

World Health Organization. (2015b). Health topics: cardiovascular diseases.<http://www.who.int/topics/cardiovascular_diseases/en/> (accessed 21 Nov. 2015).

World Health Organization. (2015d). Media centre: cardiovascular (CVDs) key facts.<http://www.who.int/mediacentre/factsheets/fs317/en/> (accessed 21 Nov. 2015).

World Health Organization. (2015c). NCDs Ghana statistic. [http://www.who.int/nmh/countries/gha_en.pd](http://www.who.int/nmh/countries/gha_en.pdf)f?ua=1 (accessed 21 Nov. 2015).

World Health Organization. (2015a). Noncommunicable diseases.<http://www.who.int/mediacentre/factsheets/fs355/en/> (accessed 21 Nov. 2015).

World Health Organiztion. (2016). School health and youth health promotion.<http://www.who.int/school_youth_health/en/> (accessed 19 Apr. 2016).

World Health Rankings: Health Profile Ghana. (2014).<http://www.worldlifeexpectancy.com/country-health-profile/ghana> (accessed 26

Jan. 2016).

World Health Organization. (2018a). Global Health Observatory (GHO) Data. World Health Statistics 2018.<http://www.who.int/gho/publications/world_health_statistics/2018/EN_WHS20>

18_AnnexA.pdf?ua=1 (accessed 26 Nov. 2018).

World Health Organization. (2018b). Global status report on alcohol and health. https:/[/www.who.int/substance_abuse/publications/global_alcohol_report/en/](http://www.who.int/substance_abuse/publications/global_alcohol_report/en/) (accessed 1 Aug. 2019).

World Heart Federation (WHF). (2017). Cardiovascular disease risk factors. https:/[/www.world-heart-federation.org/resources/risk-factors/](http://www.world-heart-federation.org/resources/risk-factors/) (accessed 1 Aug.

2019).

World Heart Federation (WHF). (2015). Heart fact sheet on cardiovascular diseases.<http://www.world-heart-federation.org/heart-facts/fact-sheets/cardiovascular-> disease-terms. (accessed 21 Nov. 2015).

Xu, F., Ware, R. S., Leslie, E., Tse, L. A., Wang, Z., Li, J., & Wang, Y. (2015b).

Effectiveness of a randomized controlled lifestyle intervention to prevent obesity among Chinese primary school students: Click-obesity study. *PLoS ONE*, *10*(10),

1–12. doi:10.1371/journal.pone.0141421.

Xu, X. L., Zhu, R., Sharma, M., Deng, S., Liu, S., Liu, D.-Y., & Zhao, Y. (2015a).

Smoking attitudes between smokers and non-smoker secondary school students in three geographic areas of China: a cross-sectional survey based on social cognitive theory. *The Lancet*, *386*, S78. doi:10.1016/S0140-6736(15)00659-5.

Yang, Q., Zhang, Z., Gregg, E. W., Flanders, W. D., Merritt, R., & Hu, F. B. (2014).

Added sugar intake and cardiovascular diseases mortality among US adults. *Jama Internal Medicine*, *174*(4), 516–524. doi:10.1001/jamainternmed.2013.13563.

Yang, Q., Zhang, Z., Kuklina, E. V., Fang, J., Ayala, C., Hong, Y., & Merritt, R. (2012). Sodium intake and blood pressure among US children and adolescents. *Paediatrics*, *130*(4), 611–619. doi:10.1542/peds.2011-3870.

Ylimaki, E. L., Kanste, O., Heikkinen, H., Bloigu, R., & Kyngas, H. (2015). The effects of a counselling intervention on lifestyle change in people at risk of cardiovascular disease. *European Journal of Cardiovascular Nursing*, *14*(2),

153–161. doi:10.1177/1474515114521725.

Yu, S. S. K., Castillo, D. C., Courville, A. B., & Sumner., A. E. (2012). The triglyceride paradox in people of African descent. *Metabolic Syndrome and Related Disorders*, *10*(2), 77–82. doi:10.1089/met.2011.0108.

Yusuf, S., Wood, D., Ralston, J., & Reddy, K. S. (2015). The world heart federation’s

vision for worldwide cardiovascular disease prevention. *The Lancet*, *386*(9991),

399–402. doi:10.1016/S0140-6736(15)60265-3.

Zaborskis, A., Lagunaite, R., Busha, R., & Lubiene, J. (2012). Trend in eating habits among Lithuanian school-aged children in context of social inequality: three cross-sectional surveys 2002, 2006 and 2010. *BMC Public Health*, *19*, 1–12. doi:10.1186/1471-2458-12-52.

**APPENDICES**

**Appendix A**

**QUESTIONNAIRE**

**EFFECTIVENESS OF BEHAVIORAL MODIFICATION INTERVENTION TO REDUCE CARDIOVASCULAR DISEASE RISK FACTORS AMONG PUBLIC SECONDARY SCHOOL STUDENTS IN BRONG AHAFO, GHANA.**

**Instructions (To be read out to students)**

The purpose of this questionnaire is to evaluate the effectiveness of a behavioral

modification intervention module on reducing cardiovascular disease risk factors among secondary school students in Brong Ahafo Region. Truthful responses are required. Information is strictly for research purposes and confidentiality is assured.

**SECTION 1: BACKGROUND INFORMATION** School code: Student code: District:

**Please read the questions clearly and circle the appropriate response**

**1*.* Date of birth (DD MM YYY)**

**DOB**

**2. Age (in years)**

**AGE**

**3. Gender** 1. Male 2. Female **GENDER**

**4. In what grade are you?** 1. SHS

1

2. SHS

2

3. SHS

3

**GRADE**

**5. Ethnicity ETHNIC**

Other

| 11. Akan | 12. Banda | 13. Basare | 14. Bawule | | 15. Bimoba |
| --- | --- | --- | --- | --- | --- |
| 16. Chokosi | 17. Degarte | 18. Dagomba | 19. Ewe | | 20. Frafra |
| 21. Fulani | 22. Ga-Adangbe | 23. Gonja | 24. Grusi/ | | 25.  Konkomba |
|  |  |  | Kasina/Namkana | |  |
| 26. Kusasi | 27. Mamprusi | 28. Mo | 29. Pantra | | 30. Sefwi/  Wassa/Aowin |
|  |  |  |  |  |  |
| 31. Sisala | 32. Wala | 33. Wangara/ | 34. |  | |
|  |  | Dwula/ | (specify) |  |  |
|  |  | Zambraba |  |  |  |

**6. Which region are you from? REGION**

| 1. Ashanti | 2. Brong Ahafo | 3. Central | 4. Eastern |
| --- | --- | --- | --- |
| 5.Greater  Accra | 6. Northern | 7. Upper East | 8. Upper West |
| 9. Volta | 10. Western | 11. Other (specify) |  |

**7. Monthly pocket money POCMONEY**

| 1. < 50 | 2. 50-99 | 3. 100-149 | 4. 150-  199 | 5. 200-249 | 6. >250 |
| --- | --- | --- | --- | --- | --- |

**8. Father’s Education FAEDU**

| 1. None | 2. Primary school | 3. JHS | 4. SSS |
| --- | --- | --- | --- |
| 5. Diploma | 6. HND | 7. Degree | 8. Master and higher |

**9. Father’s Employment FAEMPLOY**

| 1. | Not 2. Clerical/Secretarial | | 3. Trader | 4. Farmer | |
| --- | --- | --- | --- | --- | --- |
| employed |  |  |  |  |  |
| 5. | | 6. Professional (teacher, banker,  health worker, administrative, etc) | 7.Labourer/ | 8. | Other |
| Tailor/Barber | |  | Domestic | (Specify) |  |
|  | |  | worker |  |  |

**10. Mother’s Education MOEDU**

| 1. None | 2. Primary school | 3. JHS | 4. SSS |
| --- | --- | --- | --- |
| 5. Diploma | 6. HND | 7. Degree | 8. Master and higher |

**11. Mother’s Employment MOEMPLOY**

| 1. | Not 2. Clerical/Secretarial | | 3. Trader/ | 4. Farmer | |
| --- | --- | --- | --- | --- | --- |
| employed |  |  | Food seller |  |  |
| 5. Seamstress/ | | 6. Professional (teacher, banker,  health worker, administrative, etc) | 7.Labourer/ | 8. | Other |
| Hairdresser | |  | Domestic | (Specify) |  |
|  | |  | worker |  |  |

**12. Who do you live with? LIVEWITH**

| 1. Both parents | 2. Father | 3. Mother |
| --- | --- | --- |
| 4. Aunt/Uncle | 5. Grandmother/father | 6. Other (Specify) |

**13. How many people are living with in your home household, including you**

**LIVEHOME**

| 1. One (If you are the only | 2. Two | 3. | 4. Four | 5. Five | 6. | 7. |
| --- | --- | --- | --- | --- | --- | --- |
| one) |  | Three |  |  | Six | Seven |

**14. Is there family history of obesity?**

1. Yes 2. No **FHBESITY**

**15. Is there family history of hypertension?** 1. Yes 2. No **HISBP**

**16. Is Cardiovascular Disease a major public health problem of today? PHCVD**

1. Yes 2. No 3. I don’t know

**17. How do you describe your health? HEALTH**

| 1. Excellent | 2.Very good | 3. Good | 4. Fair | 5. Poor |
| --- | --- | --- | --- | --- |

**SECTION 2 : DIET ASSESSMENT**

This refers to your diet over the past 7 days (one week). Please write the number of each food item consumed and circle the corresponding frequency of consumption

| **18. Fruits &**  **Vegetables** | **Handy measure** | **Number**  **consumed** | **(1) Never** | **(2) 2-3**  **Times per week** | **(3) 4-5**  **Times per week** | **(4) 6 Times**  **per week** | **(5) Daily** |
| --- | --- | --- | --- | --- | --- | --- | --- |
| Banana | 3 Middle finger Size |  | 1 | 2 | 3 | 4 | 5 |
| Mango | Orange average size |  | 1 | 2 | 3 | 4 | 5 |
| Pawpaw | Empty sardine tin size |  | 1 | 2 | 3 | 4 | 5 |
| Pineapple | Empty sardine tin size |  | 1 | 2 | 3 | 4 | 5 |
| Orange | Orange average size |  | 1 | 2 | 3 | 4 | 5 |
| Pear | Large orange size |  | 1 | 2 | 3 | 4 | 5 |
| Tangerine | Empty milk tin size |  | 1 | 2 | 3 | 4 | 5 |
| Watermelon | Empty sardine tin size |  | 1 | 2 | 3 | 4 | 5 |
| Cabbage | Stewing spoon level |  | 1 | 2 | 3 | 4 | 5 |
| Carrots | Stewing spoon level |  | 1 | 2 | 3 | 4 | 5 |
| Cassava  leaves | Stewing spoon level |  | 1 | 2 | 3 | 4 | 5 |
| Kontomire  (green leafy vegetable) | Stewing spoon level |  | 1 | 2 | 3 | 4 | 5 |
| Cucumber | Empty small tomato tin  size |  | 1 | 2 | 3 | 4 | 5 |
| Okro/okra | Stewing spoon level |  | 1 | 2 | 3 | 4 | 5 |
| Onions | Table spoon/big spoon  level |  | 1 | 2 | 3 | 4 | 5 |

**FAV1**

**FAV2**

**FAV3**

**FAV4**

**FAV5**

**FAV6**

**FAV7**

**FAV8**

**FAV9**

**FAV10**

**FAV11**

**FAV12**

**FAV13**

**FAV14**

**FAV15**

**FAV16**

| Pepper | | Small finger size |  | 1 | 2 | 3 | 4 | 5 |
| --- | --- | --- | --- | --- | --- | --- | --- | --- |
| Tomatoes | | Empty milk tin size |  | 1 | 2 | 3 | 4 | 5 |
| **19. Tubers** | | **Handy measure** | **Number** | **(1) Never** | **(2) 2-3** | **(3) 4-5** | **(4) 6 Times** | **(5) Daily** |
| **and Plantain** | |  | **consumed** |  | **Times** | **Times** | **per week** |  |
|  | |  |  |  | **per week** | **per week** |  |  |
| Cassava | | Empty sardine tin size |  | 1 | 2 | 3 | 4 | 5 |
| Plantain | | Medium size |  | 1 | 2 | 3 | 4 | 5 |
| Cocoyam | | Standard egg size |  | 1 | 2 | 3 | 4 | 5 |
| Sweet potato | | Standard egg size |  | 1 | 2 | 3 | 4 | 5 |
| White potato | | Standard egg size |  | 1 | 2 | 3 | 4 | 5 |
| Yam | | Empty sardine tin size |  | 1 | 2 | 3 | 4 | 5 |
| **20. Cereals** | | **Handy measure** | **Number** | **(1) Never** | **(2) 2-3** | **(3) 4-5** | **(4) 6 Times** | **(5) Daily** |
| **& Grains** | |  | **consumed** |  | **Times** | **Times** | **per week** |  |
|  | |  |  |  | **per week** | **per week** |  |  |
| Maize | | Empty milk tin size |  | 1 | 2 | 3 | 4 | 5 |
| Millet | | Empty milk tin size |  | 1 | 2 | 3 | 4 | 5 |
| Oats | | Soup spoon/ladle |  | 1 | 2 | 3 | 4 | 5 |
| Rice (white) | | Soup spoon/ladle |  | 1 | 2 | 3 | 4 | 5 |
| Rice (brown) | | Soup spoon/ladle |  | 1 | 2 | 3 | 4 | 5 |
| Sorghum |  | Soup spoon/ladle |  | 1 | 2 | 3 | 4 | 5 |
| (similar | to |  |  |  |  |  |  |  |
| corn) |  |  |  |  |  |  |  |  |
| Wheat | | Soup spoon/ladle |  | 1 | 2 | 3 | 4 | 5 |
| **21. Legumes** | | **Handy measure** | **Number** | **(1) Never** | **(2) 2-3** | **(3) 4-5** | **(4) 6 Times** | **(5) Daily** |
|  |  |  | **consumed** |  | **Times** | **Times** | **per week** |  |
|  |  |  |  |  | **per week** | **per week** |  |  |
| Agushie | | Stewing spoon level |  | 1 | 2 | 3 | 4 | 5 |

**FAV17**

**TUP1**

**TUP2**

**TUP3**

**TUP4**

**TUP5**

**TUP6**

**CEG1**

**CEG2**

**CEG3**

**CEG4**

**CEG5**

**CEG6**

**CEG7**

**LEG1**

**LEG2**

| Bambara | | Stewing spoon level |  | 1 | 2 | 3 | 4 | 5 |
| --- | --- | --- | --- | --- | --- | --- | --- | --- |
| beans | |  |  |  |  |  |  |  |
| Cowpea | | Stewing spoon level |  | 1 | 2 | 3 | 4 | 5 |
| Groundnut | | Stewing spoon level |  | 1 | 2 | 3 | 4 | 5 |
| Palm nut | | Empty milk tin size |  | 1 | 2 | 3 | 4 | 5 |
| Soyabeans |  | Stewing spoon level |  | 1 | 2 | 3 | 4 | 5 |
| (similar | to |  |  |  |  |  |  |  |
| and used | as |  |  |  |  |  |  |  |
| substitute | to |  |  |  |  |  |  |  |
| corn) |  |  |  |  |  |  |  |  |
| Tiger nuts | | Stewing spoon level |  | 1 | 2 | 3 | 4 | 5 |
| **22. Animal** | | **Handy measure** | **Number** | **(1) Never** | **(2) 2-3** | **(3) 4-5** | **(4) 6 Times** | **(5) Daily** |
| **Products** | |  | **consumed** |  | **Times** | **Times** | **per week** |  |
|  | |  |  |  | **per week** | **per week** |  |  |
| Eggs | | Egg size |  | 1 | 2 | 3 | 4 | 5 |
| Fish | | Empty sardine tin size |  | 1 | 2 | 3 | 4 | 5 |
| Milk | | Small tin size |  | 1 | 2 | 3 | 4 | 5 |
| Milk powder | | Tablespoon level |  | 1 | 2 | 3 | 4 | 5 |
| Chicken | | Match box size |  | 1 | 2 | 3 | 4 | 5 |
| Red | | Standard egg size |  | 1 | 2 | 3 | 4 | 5 |
| meat/Beef | |  |  |  |  |  |  |  |
| Salted fish | | Match box size |  | 1 | 2 | 3 | 4 | 5 |
| (such as | |  |  |  |  |  |  |  |
| **koobi**-salted | |  |  |  |  |  |  |  |
| tilapia, | |  |  |  |  |  |  |  |
| **momoni**,- | |  |  |  |  |  |  |  |
| salted fish | |  |  |  |  |  |  |  |
| left to | |  |  |  |  |  |  |  |

**LEG3**

**LEG4**

**LEG5**

**LEG6**

**LEG7**

**ANP1**

**ANP2**

**ANP3**

**ANP4**

**ANP5**

**ANP6**

**ANP7**

**ANP8**

**ANP9**

**FAL1**

**FAL2**

**FAL3**

**FAL4**

**FAL5**

**FAL6**

**FAL7**

**FAL8**

**SDS1**

**SDS2**

| ferment, | | |  |  |  |  |  |  |  |
| --- | --- | --- | --- | --- | --- | --- | --- | --- | --- |
| **kako**-salted | | |  |  |  |  |  |  |  |
| herring) | | |  |  |  |  |  |  |  |
| Seafood | |  | Small finger size |  | 1 | 2 | 3 | 4 | 5 |
| (such |  | as |  |  |  |  |  |  |  |
| shrimp, |  |  |  |  |  |  |  |  |  |
| lobster, | etc) |  |  |  |  |  |  |  |  |
| Snail | | | Small tomato tin size |  | 1 | 2 | 3 | 4 | 5 |
| **23. Fats and** | | | **Handy measure** | **Number** | **(1) Never** | **(2) 2-3** | **(3) 4-5** | **(4) 6 Times** | **(5) Daily** |
| **oils** | | |  | **consumed** |  | **Times** | **Times** | **per week** |  |
|  | | |  |  |  | **per week** | **per week** |  |  |
| Coconut oil | | | Stewing spoon level |  | 1 | 2 | 3 | 4 | 5 |
| Groundnut | | | Stewing spoon level |  | 1 | 2 | 3 | 4 | 5 |
| oil | | |  |  |  |  |  |  |  |
| margarine | | | Stewing spoon level |  | 1 | 2 | 3 | 4 | 5 |
| Palm | kernel | | Stewing spoon level |  | 1 | 2 | 3 | 4 | 5 |
| oil |  |  |  |  |  |  |  |  |  |
| Palm oil | | | Stewing spoon level |  | 1 | 2 | 3 | 4 | 5 |
| Refined | | | Stewing spoon level |  | 1 | 2 | 3 | 4 | 5 |
| vegetable oil | | |  |  |  |  |  |  |  |
| Sheabutter | | | Stewing spoon level |  | 1 | 2 | 3 | 4 | 5 |
| oil | | |  |  |  |  |  |  |  |
| Soya oil | | | Stewing spoon level |  | 1 | 2 | 3 | 4 | 5 |
| **24.** | **Soft** | | **Handy measure** | **Number** | **(1) Never** | **(2) 2-3** | **(3) 4-5** | **(4) 6 Times** | **(5) Daily** |
| **Drinks** |  | **&** |  | **consumed** |  | **Times** | **Times** | **per week** |  |
| **Sweets** |  |  |  |  |  | **per week** | **per week** |  |  |
| Chocolate | | | 1 segment size |  | 1 | 2 | 3 | 4 | 5 |
| Energy drink | | | 350 mls pack size |  | 1 | 2 | 3 | 4 | 5 |

**SDS3**

| Fruit juice | 250 mls pack size | |  | 1 | 2 | 3 | 4 | | 5 |
| --- | --- | --- | --- | --- | --- | --- | --- | --- | --- |
| Malt drink | 330 mls bottle size | |  | 1 | 2 | 3 | 4 | | 5 |
| Minerals | 300 mls bottle size | |  | 1 | 2 | 3 | 4 | | 5 |
| (coke, fanta, |  |  |  |  |  |  |  |  |  |
| sprite, etc) |  |  |  |  |  |  |  |  |  |
| Toffees | 1 average toffee size | |  | 1 | 2 | 3 | 4 | | 5 |
| Sugar (you | Teaspoon size | |  | 1 | 2 | 3 | 4 | | 5 |
| add to food) |  |  |  |  |  |  |  |  |  |
| **25. Snacks** | **Handy measure** | | **Number** | **(1) Never** | **(2) 2-3** | **(3) 4-5** | **(4) 6 Times** | | **(5) Daily** |
|  |  |  | **consumed** |  | **Times** | **Times** | **per week** | |  |
|  |  |  |  |  | **per week** | **per week** |  | |  |
| Biscuit | **1 biscuit** | **(cream** |  | **1** | **2** | **3** | **4** | | **5** |
|  | **crackers size)** |  |  |  |  |  |  |  |  |
| Cakes | Orange size | |  | 1 | 2 | 3 | 4 | | 5 |
| Chips | Empty sardine tin size | |  | 1 | 2 | 3 | 4 | | 5 |
| Doughnuts | Large egg size | |  | 1 | 2 | 3 | 4 | | 5 |
| Fried chicken | Match box size | |  | 1 | 2 | 3 | 4 | | 5 |
| Fried egg | Standard egg size | |  | 1 | 2 | 3 | 4 | | 5 |
| Ice cream | Empty small tomato tin  size | |  | 1 | 2 | 3 | 4 | | 5 |
| Meat/sausage | Empty small tomato tin  size | |  | 1 | 2 | 3 | 4 | | 5 |
| khebab |  |  |  |  |  |  |  |  |  |
| Popcorn | Empty milk tin size | |  | 1 | 2 | 3 | 4 | | 5 |
|  |  |  |  |  |  |  |  |  |  |
| **26. Water** | **Handy measure** | | **Number** | **(1)** | **(2) 1 per** | **(3) 2-3** | **(4)** | **4-5** | **(5) 6 or** |
| **consumption** |  |  | **consumed** | **Rarely** | **day** | **Times** | **Times** | **per** | **more times** |
|  |  |  |  |  |  | **per day** | **day** |  | **per day** |
| Water (daily) | 500 mls (sachet water) | |  | 1 | 2 | 3 | 4 | | 5 |

**SDS4**

**SDS5**

**SDS6**

**SDS7**

**SKS1**

**SKS2**

**SKS3**

**SKS4**

**SKS5**

**SKS6**

**SKS7**

**SKS8**

**SKS9**

**WATER**

**27. How often do you normally add salt to your already prepared/cooked meal? SALTINT**

| 1. Never | 2. Rarely | 3. Occasionally | 4. Very often |
| --- | --- | --- | --- |

**28. Please circle the one you prefer to eat at any time. Circle one response per row**

| 1. Banana | **Or** | 2. Biscuit |
| --- | --- | --- |
| 1. Ice cream | **Or** | 2. Orange |
| 1. Watermelon | **Or** | 2. Soft drink |
| 1. Baked chicken | **Or** | 2. Fried chicken |
| 1. White bread | **Or** | 2. Wheat bread |
| 1. Chips | **Or** | 2. Corn |

**BANBS ICEOR**

**WATSOF**

**BAKED WHITEB CHIPCON**

**29. Circle the one you think is good for your health. Circle one response per row**

| 1. Banana | **Or** | 2. Biscuit |
| --- | --- | --- |
| 1. Ice cream | **Or** | 2. Orange |
| 1. Watermelon | **Or** | 2. Soft drink |
| 1. Baked chicken | **Or** | 2. Fried chicken |
| 1. White bread | **Or** | 2. Wheat bread |
| 1. Chips | **Or** | 2. Corn |

**BANBS2**

**ICEOR2**

**WATSOF2**

**BAKED2**

**WHITEB2**

**CHIPCON**

**SECTIONS 3 AND 4**

**SMOKING AND ALCOHOL**

We want to find out your knowledge, attitude and behavior relating to lifestyle and health on smoking and alcohol. Please do not write your name on the questionnaire. Confidentiality is assured. Please answer honestly to all questions as they appear. This is not a test, there are no right or wrong answers.

**SECTION 3: SMOKING**

**The following questions ask about your use of tobacco.**

**30. Have you ever tried cigarette smoking, even one or two puffs? TRIEDSMO**

1. Yes 2. No

**31. Do you still smoke?** 1. Yes 2. No **STILLSMO**

**32. How old were you when you first tried a cigarette?**

3. NA **AGESMO**

**33. During the past 30 days (one month) on, how many days did you smoke cigarettes?**

**DAYSM**

99. NA Circle 99 if not applicable

**34. During the past 30 days (one month), on the days you smoked, how many cigarettes did you usually smoke?**

99. NA Circle 99 if not applicable

**NUMSMO**

**35. Where do you smoke most often? (SELECT ONLY ONE RESPONSE) WHERESMO**

| 1. At home | 2. At school (inside  premises) | | 3. At school | 4. At friends’  place |
| --- | --- | --- | --- | --- |
|  |  |  | (outside premises) |  |
| 5. At social | 6. In public | places 7.Other (specify)  town, shops, | | 99. NA |
| events (e.g. fun | (e.g. in |  |  |  |
| fairs, games, etc) | streets, near |  |  |  |
|  | etc) |  |  |  |

**36. Do your parents (or stepparents/guardians who stay at your home)**

**smoke?**

**PASMOKE**

| 1. None | | | 2. Both | | 3. Father or stepfather only |
| --- | --- | --- | --- | --- | --- |
| 4. Mother | or | stepmother | 5. I | don’t 6. Other (Specify) | |
| only |  |  | know |  |  |

**37. If one of your best friends offered you a cigarette, would you smoke it? OFFERCIG**

| 1. Definitely not | 2. Probably not | 3. Probably yes | 4. Definitely yes |
| --- | --- | --- | --- |

**38. Do you think you will smoke a cigarette at any time during the next 12 months?**

**SMOKETM**

| 1. Definitely not | 2. Probably not | 3. Probably yes | 4. Definitely yes |
| --- | --- | --- | --- |

**39. Do you think you will be smoking cigarettes 5 years from now? SMOKEFY**

| 1. Definitely not | 2. Probably not | 3. Probably yes | 4. Definitely yes |
| --- | --- | --- | --- |

**40. Do you think it would be difficult to quit once someone has started smoking?**

**QUITSMO**

| 1. Definitely not | 2. Probably not | 3. Probably yes | 4. Definitely yes |
| --- | --- | --- | --- |

**41. Does smoking cigarette help people feel more or less comfortable at celebrations, parties, or in other social gatherings?**

**MLCOMF**

| 1. Definitely not | 2. Probably not | 3. Probably yes | 4. Definitely yes |
| --- | --- | --- | --- |

**42. Do you think smoking cigarettes makes boys look more or less attractive?**

**BATTRAC**

| 1. Definitely not | 2. Probably not | 3. Probably yes | 4. Definitely yes |
| --- | --- | --- | --- |

**43. Do you think smoking cigarettes makes girls look more or less attractive?**

**GATTRAC**

| 1. Definitely not | 2. Probably not | 3. Probably yes | 4. Definitely yes |
| --- | --- | --- | --- |

**44. Do you think cigarettes smoking is harmful to your health? SMOHARM**

| 1. Definitely not | 2. Probably not | 3. Probably yes | 4. Definitely yes |
| --- | --- | --- | --- |

**45. Do any of your closet friends smoke? FRISMOKE**

| 1. Definitely not | 2. Probably not | 3. Probably yes | 4. Definitely yes |
| --- | --- | --- | --- |

**46. Do you think it is safe to smoke for only a year or two as long as you quit after that?**

**SMOKEYR**

| 1. Definitely not | 2. Probably not | 3. Probably yes | 4. Definitely yes |
| --- | --- | --- | --- |

**47. Are you in favor of banning smoking in enclosed public places (such as in schools restaurants, buses, gyms and sports arenas)?**

**BANSMO**

1.Yes in all enclosed places without exceptions

2. Yes in all enclosed places, but allowing for designated areas for smokers

3. No

**SECTION 4: ALCOHOL**

The following questions ask about your use of alcohol. Alcohol includes beers, wine,

‘’pito’’, spirits, ‘’akpeteshie’’, and other alcoholic drinks.

**48. Have you ever taken alcohol before?**

1. Yes 2. No **EVERALCO**

**49. Do you still take alcohol?**

1. Yes

2. 99. No NA

Circle 99 if not applicable

**STILLALC**

**50. Please write the number of each alcohol consumed and circle the corresponding frequency of consumption**

| **50. Alcohol** | **Handy** | **Number** | **(1).** | **(2).** | **(3).** | **(4).** | **(5).** | **(6). 6** | **(7).** | **(99).** |
| --- | --- | --- | --- | --- | --- | --- | --- | --- | --- | --- |
|  | **measure** | **consumed** | **Never** | **once a** | **once** | **2-3** | **4-5** | **times** | **Daily** | **NA** |
|  |  |  |  | **month** | **a** | **times** | **times** | **per** |  |  |
|  |  |  |  |  | **week** | **per** | **per** | **week** |  |  |
|  |  |  |  |  |  | **week** | **week** |  |  |  |
| **Beers** | Large | 1 | | 2 | 3 | 4 | 5 | 6 | 7 | 99 |
|  | bottle |  |  |  |  |  |  |  |  |  |
| **Stout** | Small | 1 | | 2 | 3 | 4 | 5 | 6 | 7 | 99 |
| **(Guinness,** | bottle |  |  |  |  |  |  |  |  |  |
| **etc )** |  |  |  |  |  |  |  |  |  |  |
| **Spirits** | Tots | 1 | | 2 | 3 | 4 | 5 | 6 | 7 | 99 |
| **(akpeteshie,** |  |  |  |  |  |  |  |  |  |  |
| **etc)** |  |  |  |  |  |  |  |  |  |  |
| **Wine** | 1 | 1 | | 2 | 3 | 4 | 5 | 6 | 7 | 99 |
|  | medium |  |  |  |  |  |  |  |  |  |
|  | glass |  |  |  |  |  |  |  |  |  |
| **Pito** | 500mls | 1 | | 2 | 3 | 4 | 5 | 6 | 7 | 99 |
|  | (sachet |  |  |  |  |  |  |  |  |  |
|  | water) |  |  |  |  |  |  |  |  |  |
| **Palm wine** | 500mls | 1 | | 2 | 3 | 4 | 5 | 6 | 7 | 99 |
|  | (sachet |  |  |  |  |  |  |  |  |  |
|  | water) |  |  |  |  |  |  |  |  |  |

**BEERS**

**STOUT**

**SPIRITS**

**WINE**

**PITO**

**51. How old were you when you first tried alcohol?**

**99. NA AGEALC**

**52. Do your parents (or stepparents/guardians) who stay at your home take alcohol?**

**PASALC**

| 1. None | 2. Both | 3. Father or stepfather only |
| --- | --- | --- |
| 4. Mother or  stepmother only | 5. I don’t know | 6. Other (Specify) |

**53. Do any of your friends take alcohol?**

**1.Yes 2. No FRIALC**

**54. Have you ever had so much alcohol that you were drunk?**

**SOALC**

| 1. No, never | | 2. Yes, once | 3. Yes, | 2-3 |
| --- | --- | --- | --- | --- |
|  |  |  | times |  |
| 4. Yes, | 4-10 5. Yes, more than 10  times | | 99. NA | |
| times |  |  |  |  |

**55. Do think alcohol is harmful to one’s**

**health?**

**1.Yes 2. No HARMALC**

**SECTION 5: PHYSICAL ACTIVITY**

We want to find out about your physical activity level during the **past 7 days (in the last week).** This will include sports or dance that makes you sweat or games that makes you breathe hard like skipping, running, climbing, among others.

**Remember**

1. This is not a test, there are no right or wrong answers.

2. Kindly answer the questions as honestly and accurately as you can.

**56. Physical activity in your spare time: Have you done any of the following activities in thepast 7 days (last week)? If yes, how many times? (Please circle one response per row)**

|  | **1. No** | **2. 1-2** | **3. 3-4** | **4. 5-6** | **5. 7 or more** |
| --- | --- | --- | --- | --- | --- |
| 1. Skipping | 1 | 2 | 3 | 4 | 5 |
| 2.Walking for exercise | 1 | 2 | 3 | 4 | 5 |
| 3. Tag | 1 | 2 | 3 | 4 | 5 |
| 4. Bicycling | 1 | 2 | 3 | 4 | 5 |
| 5. Jogging/running | 1 | 2 | 3 | 4 | 5 |
| 6. Aerobics | 1 | 2 | 3 | 4 | 5 |
| 7. Dance | 1 | 2 | 3 | 4 | 5 |
| 8. Football/Netball | 1 | 2 | 3 | 4 | 5 |
| 9. Volleyball | 1 | 2 | 3 | 4 | 5 |

**PHY1**

**PHY2**

**PHY3**

**PHY4**

**PHY5**

**PHY6**

**PHY7**

**PHY8**

**PHY9**

**57. During the last 7 days, during your physical education (PE) classes, how often PEPA**

**were you very active (playing hard, running, jumping, throwing)? (Please circle one only)**

| 1. I don’t do PE | 2. Hardly ever | 3. Sometimes | 4. Quite often | 5. Always |
| --- | --- | --- | --- | --- |

**58. During the last 7 days, what did you normally do at lunch (besides eating lunch)? (Please circle one only) PELUNCH**

| 1. Sat down (talking, reading,  doing school work) | 2. Stood around or  walked around | 3. Ran or played a little  bit |
| --- | --- | --- |
| 4. Ran around and played  quite a bit | 5. Ran and played  hard most of the time |  |

**59. During the last 7 days on, how many days right after school, did you do sports, dance, or play games in which you were very active? (Please circle**

**one only) PEAFTERS**

| 1. None | 2. 1 time last week | 3. 2 or 3 times last  week |
| --- | --- | --- |
| 4. 4 times last week | 5. 5 times last week |  |

**60. During the last 7 days on, how many days evenings did you do sports, PEEVEN**

**dance, or play games in which you were very active? (Please circle one only)**

| 1. None | 2. 1 time last week | 3. 2 or 3 times last  week |
| --- | --- | --- |
| 4. 4 or 5 times last week | 5. 6 or 7 times last  week |  |

**61. On the last weekend, how many times did you do sports, dance, or play PEWKD**

**games in which you were very active? (Please circle one only).**

| 1. None | 2. 1 time | 3. | 2-3 | 4. | 4-5 | 5. 6 or more times |
| --- | --- | --- | --- | --- | --- | --- |
|  |  | times |  | times |  |  |

**62. Which one of the following best describes you in the last 7 days? Read all five statements before deciding on the one answer that describes you.**

| 1. All or most of my free time was spent doing things that involve little physical  effort |
| --- |
| 2. I sometimes (1 - 2 times last week) did physical things in my free time  (e.g. played sports, went running, bike riding, did aerobics) |
| 3. I often (3 - 4 times last week) did physical things in my free time. |
| 4. I quite often (5 - 6 times last week) did physical things in my free time |
| 5. I very often (7 or more times last week) did physical things in my free time |

**PESEVEN**

**63. Mark how often you did physical activity (like playing sports, games, doing dance, or any other physical activity) for each day last week.**

|  | **1. None** | **2. Little bit** | **3. Medium** | **4. Often** | **5. Very often** |
| --- | --- | --- | --- | --- | --- |
| Monday | 1 | 2 | 3 | 4 | 5 |
| Tuesday | 1 | 2 | 3 | 4 | 5 |
| Wednesday | 1 | 2 | 3 | 4 | 5 |
| Thursday | 1 | 2 | 3 | 4 | 5 |
| Friday | 1 | 2 | 3 | 4 | 5 |
| Saturday | 1 | 2 | 3 | 4 | 5 |
| Sunday | 1 | 2 | 3 | 4 | 5 |

**MON TUES WED THU FRI SAT**

**SUN**

**SECTION 6: CVD KNOWLEDGE**

This section is to test your knowledge on cardiovascular disease. Please

circle the appropriate answer.

**(DK = DON’T KNOW)**

**CVD1**

**CVD2**

**CVD3**

**CVD4**

**CVD5**

**CVD6**

**CVD7**

**CVD8**

**CVD9**

**CVD10**

**CVD11**

**CVD12**

**CVD13**

**CVD14**

**CVD15**

**CVD16**

**CVD17**

**CVD18**

**CVD19**

**CVD20**

|  | | **1. True** | **2. False** | **3. DK** |
| --- | --- | --- | --- | --- |
| 64 | Heart diseases/heart attacks occur in children | 1 | 2 | 3 |
| 65 | Men are more likely to develop heart disease  than women | 1 | 2 | 3 |
| 66 | Having a family member with heart disease  puts a person at a higher risk of developing heart disease | 1 | 2 | 3 |
| 67 | Exercise can cause heart disease | 1 | 2 | 3 |
| 68 | Hypertension can cause heart disease | 1 | 2 | 3 |
| 69 | Obesity can cause heart disease | 1 | 2 | 3 |
| 70 | Eating more fruits and vegetables can cause  heart disease | 1 | 2 | 3 |
| 71 | Smoking can cause heart disease | 1 | 2 | 3 |
| 72 | Alcohol can cause heart disease | 1 | 2 | 3 |
| 73 | Fatty foods can cause heart disease | 1 | 2 | 3 |
| 74 | Eating lots of fast foods can cause heart  disease | 1 | 2 | 3 |
| 75 | Heart diseases are preventable | 1 | 2 | 3 |
| 76 | Measures to prevent heart diseases should be  started right from childhood | 1 | 2 | 3 |
| 77 | **Heart disease can be prevented by:** | 1 | 2 | 3 |
| 77A | Maintaining a normal body weight | 1 | 2 | 3 |
| 77B | Exercising 1hour/per day | 1 | 2 | 3 |
| 77C | Limiting time for watching TV, computer  games, sitting activities to less than 2 hours/day | 1 | 2 | 3 |
| 77D | Avoiding smoking | 1 | 2 | 3 |
| 77E | Avoiding use of tobacco products | 1 | 2 | 3 |
| 77F | Avoiding alcohol | 1 | 2 | 3 |
| 77G | Only exercising in a gym | 1 | 2 | 3 |

**CVD21**

**CVD22**

**CVD23**

**CVD24**

**CVD25**

**CVD26**

| 78 | **The following type of diet can prevent**  **heart disease:** | 1 | 2 | 3 |
| --- | --- | --- | --- | --- |
| 78A | More fruits and vegetables | 1 | 2 | 3 |
| 78B | More salt in diet | 1 | 2 | 3 |
| 78C | Less of fried foods | 1 | 2 | 3 |
| 78D | More sweets/chocolate/ice cream | 1 | 2 | 3 |
| 78E | Less of fish | 1 | 2 | 3 |
| 78F | More of soft/carbonated drinks | 1 | 2 | 3 |

**SECTION 7: MOTIVATION**

**79. I am encouraged to live a general CVD risk factor reduction lifestyle GENCVD**

| 1. I strongly | 2. I | somewhat | 3. I somewhat agree | 4. I strongly  agree |
| --- | --- | --- | --- | --- |
| disagree | disagree |  |  |  |

**80. I am encouraged to live a CVD risk factor reduction lifestyle by my school**

**SCHCVD**

| 1. I strongly | 2. I | somewhat | 3. I somewhat agree | 4. I strongly  agree |
| --- | --- | --- | --- | --- |
| disagree | disagree |  |  |  |

**81. I am encouraged to live a CVD risk factor reduction lifestyle by my peers**

**PEERCVD**

| 1. I strongly | 2. I | somewhat | 3. I somewhat agree | 4. I strongly  agree |
| --- | --- | --- | --- | --- |
| disagree | disagree |  |  |  |

**82. I get frustrated living a CVD risk factor reduction lifestyle because I**

**have to plan it around my life.**

**PLANCVD**

| 1. I strongly | 2. I | somewhat | 3. I somewhat agree | 4. I strongly  agree |
| --- | --- | --- | --- | --- |
| disagree | disagree |  |  |  |

**83. I feel the general environment does not support me in living a CVD risk factor reduction lifestyle.**

**ENVTCVD**

| 1. I strongly | 2. I | somewhat | 3. I somewhat agree | 4. I strongly  agree |
| --- | --- | --- | --- | --- |
| disagree | disagree |  |  |  |

**84. It upsets me I have to do physical activity at least 60 minutes in a day. UPACVD**

| 1. I strongly | 2. I | somewhat | 3. I somewhat agree | 4. I strongly  agree |
| --- | --- | --- | --- | --- |
| disagree | disagree |  |  |  |

**85. It upsets me I have to stop eating unhealthy foods. UDIETCVD**

| 1. I strongly | 2. I | somewhat | 3. I somewhat agree | 4. I strongly  agree |
| --- | --- | --- | --- | --- |
| disagree | disagree |  |  |  |

**86. It upsets me I have to stop/not to initiate smoking. USMOCVD**

| 1. I strongly | 2. I | somewhat | 3. I somewhat agree | 4. I strongly  agree |
| --- | --- | --- | --- | --- |
| disagree | disagree |  |  |  |

**87. It upsets me I have to stop/not to initiate alcohol consumption.**

**UALOCVD**

| 1. I strongly | 2. I | somewhat | 3. I somewhat agree | 4. I strongly  agree |
| --- | --- | --- | --- | --- |
| disagree | disagree |  |  |  |

**88. I am worried I may not be able to live an entirely CVD risk factor reduction lifestyle all the time.**

**WRFCVD**

| 1. I strongly | 2. I | somewhat | 3. I somewhat agree | 4. I strongly  agree |
| --- | --- | --- | --- | --- |
| disagree | disagree |  |  |  |

**SECTION 8: BEHAVIOR SKILLS**

**89. How hard or easy is it for you to stay informed about CVD**

**prevention? HECVD**

| 1. Very hard | 2. Hard | 3. Easy | 4. Very easy |
| --- | --- | --- | --- |

**90. How hard or easy is it for you to get the support you need from others for reducing CVD risk factors (for example peer-friends and teachers)?**

**SUCVD**

| 1. Very hard | 2. Hard | 3. Easy | 4. Very easy |
| --- | --- | --- | --- |

**91. How hard or easy is it for you to understand living a healthy**

**lifestyles especially reducing CVD risk factors? RUCVD**

| 1. Very hard | 2. Hard | 3. Easy | 4. Very easy |
| --- | --- | --- | --- |

**92. How hard or easy is it for you to practice CVD risk factor reduction**

**lifestyle when you are extremely busy? PRACVD**

| 1. Very hard | 2. Hard | 3. Easy | 4. Very easy |
| --- | --- | --- | --- |

**93. How hard or easy is it for you to remember to apply your skills to**

**practice CVD risk factor reduction lifestyle? SKICVD**

| 1. Very hard | 2. Hard | 3. Easy | 4. Very easy |
| --- | --- | --- | --- |

**94. How hard or easy is it for you to make use your CVD risk factor reduction skills as part of your life daily lifestyle?**

**DACVD**

| 1. Very hard | 2. Hard | 3. Easy | 4. Very easy |
| --- | --- | --- | --- |

**95. How hard or easy is it for you to apply your CVD risk factor reduction skills when you are in a conducive environment?**

**CECVD**

| 1. Very hard | 2. Hard | 3. Easy | 4. Very easy |
| --- | --- | --- | --- |

**96. How hard or easy is it for you to apply your CVD risk factor reduction skills when you are NOT in a conducive environment?**

| 1. Very hard | 2. Hard | 3. Easy | 4. Very easy |
| --- | --- | --- | --- |

**NTCVD**

**97. How hard or easy is it for you to educate your peers about CVD risk factor reduction?**

**EDCVD**

| 1. Very hard | 2. Hard | 3. Easy | 4. Very easy |
| --- | --- | --- | --- |

**98. How hard or easy is it for you to practice this behavior change skills when you do not feel good emotionally (for example when you are sad, stressed out or depressed)?**

**GOCVD**

| 1. Very hard | 2. Hard | 3. Easy | 4. Very easy |
| --- | --- | --- | --- |

**SECTIONS 9 AND 10: ANTHROPOMETRIC AND BLOOD PRESSURE MEASUREMENTS TO BE TAKEN BY**

|  |  | **.** |  |
| --- | --- | --- | --- |

|  |  |  | **.** |  |
| --- | --- | --- | --- | --- |

| **FACILITATORS**  **99. Weight (kg)** |  |  |  |  | **WEIGHT** |
| --- | --- | --- | --- | --- | --- |
| **100. Height (cm)** |  |  |  |  | **HEIGHT** |
| **1ST READING** |  |  |  |  |  |
| **101a. Systolic (mmHg)** |  |  |  |  | **SYSTOLIC1** |
| **102a. Diastolic (mmHg)** |  |  |  |  | **DIASTOL1** |
| PLEASE WAIT FOR MEASUREMENT | ONE | MINUTE | BEFORE | TAKING | THE SECOND |
| **2ND READING** |  |  |  |  |  |
| **101b. Systolic (mmHg)** |  |  |  |  | **SYSTOLIC2** |
| **102b. Diastolic (mmHg)** |  |  |  |  | **DIASTOL2** |

**Thank you for taking your time to complete the questionnaire**
